# Tumor Suppressor Par-4 Regulates Complement Factor C3 and Obesity

**DOI:** 10.3389/fonc.2022.860446

**Published:** 2022-03-29

**Authors:** Nathalia Araujo, James Sledziona, Sunil K. Noothi, Ravshan Burikhanov, Nikhil Hebbar, Saptadwipa Ganguly, Tripti Shrestha-Bhattarai, Beibei Zhu, Wendy S. Katz, Yi Zhang, Barry S. Taylor, Jinze Liu, Li Chen, Heidi L. Weiss, Daheng He, Chi Wang, Andrew J. Morris, Lisa A. Cassis, Mariana Nikolova-Karakashian, Prabhakar R. Nagareddy, Olle Melander, B. Mark Evers, Philip A. Kern, Vivek M. Rangnekar

**Affiliations:** ^1^ Department of Toxicology and Cancer Biology, University of Kentucky, Lexington, KY, United States; ^2^ Department of Microbiology, Immunology and Molecular Genetics, University of Kentucky, Lexington, KY, United States; ^3^ Department of Radiation Medicine, University of Kentucky, Lexington, KY, United States; ^4^ Department of Epidemiology and Biostatistics, Memorial Sloan Kettering Cancer Center, New York, NY, United States; ^5^ Division of Internal Medicine, University of Kentucky, Lexington, KY, United States; ^6^ Barnstable Brown Diabetes and Obesity Center, University of Kentucky, Lexington, KY, United States; ^7^ Department of Pharmacology and Nutritional Sciences, University of Kentucky, Lexington, KY, United States; ^8^ Department of Computer Science, University of Kentucky, Lexington, KY, United States; ^9^ Markey Cancer Center, University of Kentucky, Lexington, KY, United States; ^10^ Department of Statistics, University of Kentucky, Lexington, KY, United States; ^11^ Department of Biostatistics, University of Kentucky, Lexington, KY, United States; ^12^ Department of Physiology, University of Kentucky, Lexington, KY, United States; ^13^ Division of Cardiac Surgery, The Ohio State University, Columbus, OH, United States; ^14^ Department of Clinical Sciences, Lund University, Malmö, Sweden; ^15^ Department of Internal Medicine, Skåne University Hospital, Malmö, Sweden; ^16^ Department of Surgery, University of Kentucky, Lexington, KY, United States

**Keywords:** hypertrophic obesity, adipocyte tissue storage, fat absorption, acylation stimulating protein, C3, Par-4

## Abstract

Prostate apoptosis response-4 (Par-4) is a tumor suppressor that induces apoptosis in cancer cells. However, the physiological function of Par-4 remains unknown. Here we show that conventional Par-4 knockout (Par-4^-/-^) mice and adipocyte-specific Par-4 knockout (AKO) mice, but not hepatocyte-specific Par-4 knockout mice, are obese with standard chow diet. Par-4^-/-^ and AKO mice exhibit increased absorption and storage of fat in adipocytes. Mechanistically, Par-4 loss is associated with *mdm2* downregulation and activation of p53. We identified complement factor *c3* as a p53-regulated gene linked to fat storage in adipocytes. *Par-4* re-expression in adipocytes or *c3* deletion reversed the obese mouse phenotype. Moreover, obese human subjects showed lower expression of Par-4 relative to lean subjects, and in longitudinal studies, low baseline Par-4 levels denoted an increased risk of developing obesity later in life. These findings indicate that Par-4 suppresses p53 and its target *c3* to regulate obesity.

## Introduction

Prostate apoptosis response-4 (Par-4, also known as PAWR) is a tumor suppressor that is ubiquitously expressed in various cell types and vertebrate tissues (1). Loss of Par-4 expression by diverse mechanisms, including methylation-dependent downregulation of the Par-4 promoter, inactivation of Par-4 protein by Akt-mediated phosphorylation, or spontaneous mutation, has been associated with diverse human cancers (2–5). Moreover, Par-4 loss in tumors is associated with increased resistance to treatment and decreased patient survival (6–8). Consistently, genetic knockout of *Par-4* in mice results in spontaneous tumors, as well as increased susceptibility to chemically- or hormone-inducible tumors in multiple tissues (9). In addition, overexpression of Par-4 induces apoptosis in cancer cell lines but not normal cells, and Par-4 transgenic mice exhibit a normal life span and cancer-free survival (10).

Par-4 is localized in multiple intracellular compartments, such as the nucleus, endoplasmic reticulum and the cytoplasm (11). Additionally, Par-4 protein is secreted by cells and can be detected in the conditioned medium of cell cultures and in mouse and human plasma (12, 13). Secreted Par-4 binds to GRP78 expressed on the surface of cancer cells and induces apoptosis (13). Par-4 is secreted by normal cells *via* the classical endoplasmic reticulum (ER)-Golgi secretory pathway, and extracellular Par-4 binds to GRP78 on the cancer cell surface to trigger apoptosis by activation of the FADD-caspase-8-caspase-3 pathway (13). Most normal cells, however, lack cell surface GRP78 and are resistant to apoptosis by secreted Par-4 (12, 13).

The key functional domains of the Par-4 protein are conserved across human, mouse and rat species and consist of a nuclear localization sequence (NLS2), a nuclear export sequence and a leucine zipper sequence at the carboxyl-terminus (5). The NLS2 domain of Par-4 permits entry of intracellular Par-4 into the nucleus (11, 14). Nuclear Par-4 functions as a transcriptional corepressor of the pro-survival gene *Bcl2* (11). However, the physiological significance of the transcriptional regulatory function of Par-4 is not well understood. As previous studies have reported crosstalk between the tumor suppressors Par-4 and p53 in secretion of Par-4 from normal cells (12), and as p53 plays diverse roles in normal tissues (15–18), we sought to determine the physiological role of Par-4 in normal tissues using an unbiased approach by generating several Par-4 knockout mouse models. Our studies indicate that Par-4 whole-body knockout mice, as well as adipocyte-specific Par-4 knockout mice develop obesity on chow diet. As Par-4 is a tumor suppressor and as obesity is linked with an increased risk of many cancers (19–23), we interrogated the obese phenotype associated with Par-4 loss in greater depth. We present evidence that Par-4 loss in adipocytes results in obesity that is associated with increased absorption of dietary fat into circulation and its storage in adipocytes to produce hypertrophic obesity in mice. The relevance of these murine results to human obesity was demonstrated in our cohort study which indicated that baseline levels of Par-4 are associated with obesity risk in lean individuals, and that Par-4 levels are lower in obese individuals relative to lean individuals. Our findings suggest an unexpected role for adipocytes in enhancing the expression of p53 and complement factor C3/acyl stimulating protein (ASP) following loss of Par-4 leading to obesity.

## Materials and Methods

### Animals

Par-4 floxed mice (Par-4^fl/fl^) were generated on a C57BL/6 background by Taconic Biosciences following the strategy described on **Figure S1A**. Par-4^fl/fl^ mice were crossed with Rosa26-Cre (from Taconic Biosciences) to generate Par-4 whole-body knockout (Par-4^-/-^) mice. Adipocyte-specific (AKO) and hepatocyte-specific Par-4 knockout (HKO) were generated by crossing Par-4^fl/fl^ mice with adiponectin-promoter-Cre mice and with albumin-promoter-Cre, respectively (in C57BL/6 background from Jackson Laboratory). Par-4/C3 double-knockout mice were generated by crossing Par-4^-/-^ with C3 whole-body knockout mouse (in C57BL/6 background from Jackson Laboratory). Par-4Ki^tg/tg^ mice with human Par-4 containing a Stop codon inserted in the Rosa26 locus were generated by Biocytogen LLC (Worcester, MA) using the targeting strategy described in **Figure S8A**. When Par-4Ki^tg/tg^ mice were crossed with AKO mice, which contained Adipoq-Cre for adipocyte-specific expression of Cre, the Stop sequence in Par-4Ki^tg/tg^ mice was removed, and expression of human Par-4 was driven in mature adipocytes.

For each mouse strain, F1 heterozygotes were crossed to each other to generate either homozygous or heterozygous offspring for the gene of interest. Both homozygous and heterozygous mice were crossed to each other to generate homozygous mice. All mice were subjected to genotyping that was performed on DNA prepared from tail snips digested with proteinase K (Sigma-Aldrich, catalog number P2308) using primer sets indicated in **Figures S1B, S3A, S6A, S8B** for Par-4^-/-^, C3^-/-^, Adiponectin-Cre, and primer sets described by Jackson Laboratory. Mice were fed standard chow diet that consists of 18% protein, 60% carbohydrates and 12% fat (Teklad, Envigo). Mouse phenotypes described were noted in each generation for over multiple generations.

Mouse body weight was measured weekly, and body composition was determined by Echo-MRI (EchoMRI-100, Echo Medical System, Houston, TX). The experiments performed on these mice were approved by the Institutional Animal Care and Use Committee of the University of Kentucky.

### Human Specimens

Human adipose tissue samples were obtained from biopsies of normal weight and obese subjects who were recruited as part of previous studies on the effects of exercise (24) or fish oils (25). All biopsies were performed before any interventions and all participants underwent an incisional abdominal adipose biopsy, under local anesthesia, for removal of adipose tissue. The subjects had no history of coronary disease, inflammatory disease, the chronic use of any anti-inflammatory medication or other medication likely to change adipocyte metabolism. The subjects were categorized as either lean (BMI < 25 kg/m^2^) or obese (BMI > 30 kg/m^2^). Plasma samples from lean and obese adults of both genders were provided by the Center for Clinical and Translational Sciences Biospecimen Core and the University of Kentucky Markey Cancer Center Biospecimen Procurement and Translational Pathology Shared Resource Facility. All patient information was de-identified and adhering to HIPAA guidelines. Additional plasma samples were obtained from The Malmö Diet and Cancer study (“Minisymposium: The Malmö Diet and Cancer Study. Design, Biological Bank and Biomarker Programme. 23 October 1991, Malmo, Sweden.” 1993). Fasted EDTA plasma samples at the baseline examination were available for Par-4 analyses by western blot analysis. Written informed consent was given by all participants and the study was approved by the Ethical Committee at Lund University, Lund, Sweden. All work was approved by the University of Kentucky Institutional Review Board.

### Cell Culture, Constructs, and Antibodies

Wild-type, Par-4^-/-^ and p53 MEFs (26) were maintained in Dulbecco’s Modification of Eagle’s Medium (DMEM) (Sigma #D6429) supplemented with 15% fetal bovine serum (FBS) (DMEM+15% FBS) for the first three passages and supplemented with 10% FBS onward.

The C3-luc construct was purchased from Addgene (#11358) and has been previously described (27). The p53 adenoviral constructs were obtained from Wafik el-Deiry (Brown University, Providence, RI) and have been previously described (28). Green Fluorescent Protein (GFP) adenovirus was obtained from Albert Baldwin (University of North Carolina, Chapel Hill, NC) and has also been described (14).

Antibodies for Par-4 N-terminal (R-334, sc-1807), GAPDH (G-9, sc-365062), leptin (sc-842, A-20), adiponectin (sc-17044R, N-20) and p21 (F-5, sc-6246) were from Santa Cruz Biotechnology. Two other Par-4 antibodies were used: a rabbit polyclonal (S4554-1) and a mouse monoclonal (TIAI2A9G9), both produced by Proteintech. The β-actin antibody (AC-74, A5316) was from Sigma-Aldrich. The C3a/ASP antibody (ab48581) was from Abcam; Apo48 (K23300R) was from Meridian Life Science; the LPL antibody (AF7197) was from R&D Systems; and the p53 antibody (1C12, 2524S) was from Cell Signaling Technologies. Mdm2 antibody was from Santa Cruz Biotechnology (sc-965). Secondary antibodies, anti-mouse-HRP (GENA931) and anti-rabbit-HRP (GENA934), were from Sigma-Aldrich; anti-chicken-HRP (A16054) was from Thermo Fisher Scientific; and anti-goat-HRP (HAF109) was from R&D Systems.

### Luciferase Reporter Assays

Luciferase reporter assays were performed as described (12) using a Steady Lite plus reporter gene assay kit (#6066751 Perkin Elmer, Waltham, MA). Briefly, cells were co-transfected with luc reporter constructs, β-gal and test driver constructs in a 96-well plate using Lipofectamine (#18324012) and Plus reagent (#11514015) from Invitrogen. At 24 h post transfection, cell lysates were collected in appropriate lysis buffer (Radio Immuno Precipitation Assay) with protease inhibitors, combined with Steady Lite reagent and analyzed using a Perkin Elmer TopCount plate reader. The signal was normalized to β-gal expression by addition of ortho-Nitrophenyl-β-galactoside substrate and analysis at 430 nm.

### Lipid Uptake in Cell Culture

Caco-2 cells (25,000 cells/300μL medium) in chamber slides were grown overnight and then treated with 50 μL of olive oil and mouse plasma (10% final concentration) for 24 h. The cells were washed three times with PBS and fixed with 10% buffered paraformaldehyde for 30 min. Slides were rinsed three times with PBS and once with sterile water. The cells were then incubated with 60% isopropanol for 5 min, the isopropanol was discarded, and the cells were then incubated with working solution of Oil Red O (ORO) for 5 min. Stock solution of ORO (Sigma) was prepared using 300 mg ORO in 100 mL of isopropanol (99%). Working solution of ORO was then prepared using 3 parts of ORO stock solution diluted in 2 parts double distilled water. After preparation, the working solution was filtrated. After ORO staining, the cells were washed with double distilled water and slides were mounted using Vectashield mounting media (Vector Labs). Pictures were taken using the NIS-Elements imaging platform at 20X magnification. For the quantitative study after ORO staining as mentioned above, cells were washed with double distilled water, the cells were dried and Oil Red O stain were extracted with 100% isopropanol for 5 min, with gentle rocking. Absorbance was read at 492 nm using Synergy HTX plate reader.

### Knockdown Assays

Wild-type MEFs were transfected 24 h after seeding, with either control siRNA (Dharmacon, D-001830-10-15), siPar-4 #1 (Dharmacon, J-063180-5) or siPar-4 #2 (Dhamarcon, J-063180-6) as previously described (13). Whole-cell lysates were collected 48h after transfection for protein and RNA extraction. RNA was subjected to RT-qPCR for the detection of complement C3 and protein lysates were subjected to Western Blot for detection of p53, mdm2 and Par-4.

### Next-Generation Sequencing and Data Analysis

Total RNA was extracted from visceral adipose tissue from three Par-4^+/+^, three Par-4^-/-^ and three AKO male mice that were 11-weeks old using the RNeasy lipid tissue mini kit (Qiagen, catalog number 74804). RNA-Seq libraries were prepared using TruSeq Stranded Total RNA Sample Prep Kit with Ribo-Zero ribosomal RNA depletion (Illumina). The manufacturer’s protocols were used to sequence ribosomal RNA-depleted libraries at 2×100-bp paired-end reads on an Illumina HiSeq 2500 in high-output mode, to an average depth of 40×10^6^ paired-end reads per sample.

For mapping, following data quality assessments, reads derived from residual rRNA were removed by aligning [Bowtie2 v2.1.0 (99)] against ribosomal RNA references derived from GENCODE/Ensembl v74 annotations. The RNA-Seq datasets were mapped to the target genome using STAR, which maps the reads genomically and resolves reads across splice junctions. We used the two-pass mapping method in which the reads are mapped twice. The first mapping pass used a list of known annotated junctions from Ensemble. Novel junctions found in the first pass were then added to the known junctions and a second mapping pass was done. After mapping we computed the expression count matrix from the mapped reads using HTSeq. The raw count matrix generated by HTSeq was then processed by normalizing the raw gene count based on the total reads for each sample, and the average gene count for each group was calculated for identifying up-regulated/down-regulated genes. Differentially expressed genes were identified at a threshold of log2 fold change is greater than 0.5.

### Real-Time Quantitative PCR Analyses

Total RNA from MEFs was prepared using the RNeasy mini kit as described in Next-Generation Sequencing and Data Analysis. A reverse transcription polymerase chain reaction (RT-PCR) was performed using the cDNA synthesis kit purchased from BioRad (#1708891, Hercules, CA). Quantitative RT-PCR was conducted using the SsoAdvanced Universal SYBR Green Supermix (#1725271) as the detection reagent on the BioRad CFX96 RT-PCR system according to manufacturer’s instructions. Data were analyzed using the ΔΔCt methods by normalized to the internal control of 18S RNA. Primers were synthesized by Integrated DNA Technologies (IDT, Caralville, IA) and their sequences are listed below (**Table 1**). A melting curve analysis of all qPCR products was conducted to confirm a single DNA duplex.

**Table 1:** Primers used in RT-qPCR reactions.

Gene	Forward Primer (5’-3’)	Reverse Primer (5’-3’)
Mouse Par-4	GCAGATCGAGAAGAGGAAGC	GTGTTTTGCTGGGTGATGG
Human Par-4	CTGCCGCAGAGTGCTTAGAT	TGCATCTTCTGCTTTCCGCT
Mouse c3	ATGCACCCGGTTCTATCATC	CCGGACATTCAGGTTGATCT
Mouse c3	AGAGGCAAGTGCTGACCAGT	CGTACTTGTGCCCCTCCTTA
Mouse factor B	ACTCGAACCTGCAGATCCAC	CCCCATTTTCAAAGTCCTG
Mouse adipsin	AAGTGAACGGCACACACG	CACCTGCACAGAGTCGTCA
Mouse p53	CCTCTGAGCCAGGAGACATT	CTTCACTTGGGCCTTCAAAA
Mouse mdm2	AGCGCAAAACGACACTTACA	ACACAATGTGCTGCTGCTTC
Human 18S	GTAACCCGTTGAACCCCATT	CCATCCAATCGGTAGTAGCG
Mouse 18S	CGCCGCTAGAGGTGAAATTCT	CGAACCTCCGACTTTCGTTCT

### Western Blot Analyses

Tissues were lysed in RIPA buffer plus protease inhibitor using a tissue grinder (Geno/Grinder^®^ 2010, Spex Sample Prep) for 30 sec at 1450 rpm. The lysates were centrifuged at 14,000 rpm for 10 min at 4°C, diluted to a concentration of 2 mg/mL using Laemmli buffer and boiled for 5 min. Proteins were resolved using SDS-PAGE, transferred to PVDF membranes and subjected to Western blot analysis. Western blots were imaged using the UVP ChemiDoc-It^®^ 810 Imager and quantified using the UVP Vision Works software.

### Chromatin Immunoprecipitation Sequencing (ChIP-Seq)

ChIP was performed utilizing a ChIP-IT High Sensitivity kit (Active Motif; Carlsbad, CA) according to the manufacturer’s instructions. Briefly, 3T3-L1 cultured cells (~80% confluency) were fixed in a formaldehyde-containing buffer that would also fix any DNA-binding protein complexes to the chromatin. These fixed cells were then lysed by repeated snap-freezing cycles and the chromatin sheared *via* sonication using a Bioruptor Pico device (Diagenode) until the resulting chromatin fragment size was approximately 400-500 bp. Aliquots of recovered sonicated chromatin (15-30 μg) were incubated with 4 μg of Par-4 antibody overnight at 4°C. The antibody-bound chromatin was pulled-down using G-protein agarose beads, the DNA was eluted and subjected to qPCR using the proprietary ChIP-verified negative and positive control primer sets obtained from Active Motif. Remaining DNA was subjected to ChIP-Seq at the University of Kentucky Markey Cancer Center Oncogenomics Shared Resource Facility. Library preparation was performed using the MicroPlex Library Preparation Ki v2 (Diagenode) according to manufacturer’s instructions. Libraries were sequenced on an Illumina HiSeq 2500 using rapid mode run with 50-bp single-reads.

For data analysis, Bowtie2 was used to map the reads. MACS2 was used for peak assignment and peak annotation was done using HOMER.

### Antibody Neutralization

To demonstrate specificity of Par-4 antibody, 2 ng of Par-4 mouse monoclonal antibody (Proteintech #TIAI2A9G9) was preincubated with either 4 ng His-Par-4 or 4 ng thioredoxin (TRX) control protein in 10 mL of nonfat dry milk for 30 minutes. Plasma of lean individuals who stayed lean (LL) or obese individuals who stayed obese (OO) individuals was subjected to SDS-PAGE, transferred to PVDF membrane and probed with Par-4 mouse monoclonal antibody + His-Par-4, Par-4 mouse monoclonal antibody + TRX control or anti-mouse secondary antibody.

### Glucose Tolerance and Insulin Test

Oral glucose tolerance test was conducted on mice fasted for 6 h before the test. An oral gavage of glucose (25%, dissolved in saline) was administered to mice (at 2 g/kg body weight) and blood glucose was measured at 0 min (before gavage), 15 min, 30 min, 60 min and 120 min post gavage using glucose test strips and TRUE result glucometer (Trividia Health; Ft. Lauderdale, FL). Insulin levels in plasma were determined by using the ultrasensitive ELISA kit (Alpco; Salem, NH) according to manufacturer’s instructions. ELISA plate readings of OD450 nm were obtained using a Spectramax M2 plate reader, running Softmax Pro 5.4.3 and standardized to a 5-parameter logistic curve.

### Studies on Lipid Uptake in Enterocytes and TG Absorption in Blood

Olive oil administration and Oil Red O (ORO) analysis was performed as previously described (29). Briefly, mice were fasted overnight, fed commercial olive oil (17 μL/g body weight) by oral gavage and euthanized either at 0 min (before gavage), or 30 min, 60 min and 120 min after gavage. The intestine was resected and washed with cold saline. The proximal segment was processed for frozen sectioning and ORO staining. ORO staining was quantified as follows: five representative regions of villi were selected from each animal for analysis of intracellular ORO staining using Halo software’s (Indica Labs) Multiplex IHC algorithm v1.2. Blood was collected in sodium citrate *via* cardiac puncture at the same time points and centrifuged at 10,000 rpm for 10 min and triglyceride levels were measured using a commercial kit purchased from Wako Diagnostics according to manufacturer instructions (# 992-02892 and #998-02992, Mountain View, CA). Whenever tyloxapol (Sigma Aldrich, T0307) was used to inhibit lipases, mice were fasted overnight and then injected i.p. with 15% w/v tyloxapol in saline at 500 mg/kg mouse body weight, 30 min prior to olive oil gavage. Blood was collected *via* tail-snip in sodium citrate at -0.5 h (before tyloxapol injection), 0 h (before olive oil dose), 0.5 h, 1 h, 2 h, 3 h and 4 h. Plasma was centrifuged at 10,000 rpm for 10 min and triglycerides were quantified as described above. Plasma was also subjected to Western blot analysis for ApoB48 to determine chylomicron secretion.

### Behavioral and Metabolic Studies

The TSE Labmaster indirect calorimetry system (TSE-systems Inc., Chesterfield, MO) was used to quantify energy intake and locomotor activity of mice housed in metabolic cages. Whole-body composition parameters, total body fat, lean body mass, body fluids and total body water were measured using an EchoMRI-100 Body Composition Analyzer unit (EchoMRI, Houston, TX). The metabolic cages and Echo-MRI equipment are located at the University of Kentucky Center for Research in Obesity and Cardiovascular Disease.

### Histologic Examination

Samples of mouse visceral adipose tissue or liver were collected and immediately fixed using 4% neutral buffered formalin for 3 days. Tissues were dehydrated and embedded in paraffin. A tissue sections were cut at 4 μm and hematoxylin and eosin staining or Par-4 immunocytochemical analysis was performed by Dana Napier in the University of Kentucky Markey Cancer Center’s Biospecimen Procurement and Translational Pathology Shared Resource Facility. The adipocyte size was quantified as follows. The tissue section was viewed at 20x magnification. Three slides from each group were included in quantification and five fields were randomly selected on each slide. For each field, 10-15 adipocytes were randomly selected and quantified using the NIS-Elements imaging platform purchased from Nikon Instruments Inc. (Melville, NY). For F4/80 staining, primary antibody from Abcam (ab100790) was used at 1:100 overnight at 4°C. Sides were imaged using NIS-Elements imaging platform and quantification of crown-like structures (CLS) was done as the ratio of CLS per total number of adipocytes in the field (10x magnification). For the frozen section and ORO staining, liver samples were immediately frozen using liquid nitrogen and stored at −80°C until use; intestine samples were fixed in 4% neutral buffered formalin for 48 h and transferred to 50% sucrose solution before freezing. Liver and intestine sections were cut at 8 μm using a Cryostat. ORO staining was performed using reagents purchased from Electron Microscopy Sciences (Hatfield, PA).

### Statistical Analysis

All experiments were performed independently at least three different times to verify the reproducibility of the findings. The data are expressed as mean ± SEM. Statistical analyses were carried out with GraphPad Prism, Microsoft Excel software or R software. P-values for comparing experimental groups were calculated using either the Student’s *t*-test with Bonferroni correction applied when appropriate or the ANOVA with Tukey’s HSD tests. The logistic regression model was used to examine the effect of Par-4 on the likelihood of being obesity-resistant adjusting for age, sex, physical activity, and energy intake.

## Results

### Par-4 Knockout Mice Exhibit Increased Fat Accumulation

**Figure 1:**
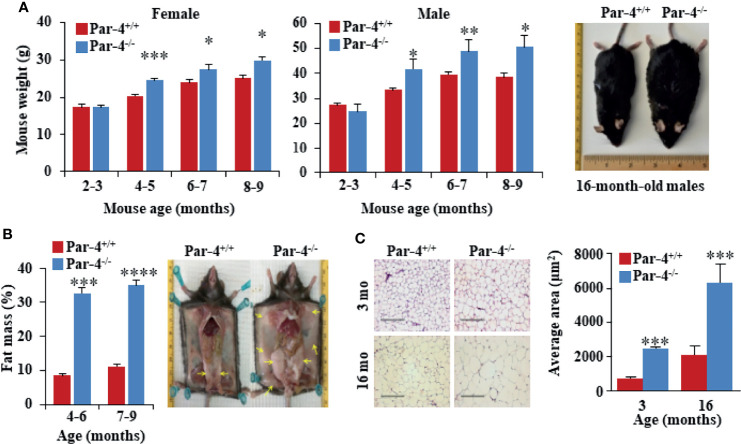
Par-4^-/-^ mice show increased fat mass and adipocyte hypertrophy. **(A)** Age- and sex-matched Par-4^-/-^ mice are significantly heavier than Par-4^+/+^ counterparts. Weight gain in female (n=8-13) and male mice (n=8) was determined over the indicated observation period; representative images of 16-month-old male mice are shown. **(B)** Par-4^-/-^ mice show increased fat mass. Echo-MRI analyses were carried out on 4-6-month-old (n=7-10) and 7-9-month-old mice male (n=14). Subcutaneous and abdominal fat (yellow arrows) in a pair of 16-month-old male mice are shown. **(C)** Hypertrophy of adipocytes in Par-4^-/-^ mice. Visceral adipose tissue from 3- or 16-month-old male mice was subjected to H&E staining and cell size was quantified (n=8 mice). Mean ± SEM, *P < 0.05, **P < 0.01, ***P < 0.001, ****P < 0.0001 by the Student’s *t*-test or with Bonferroni method adjustment for multiple comparisons. Also see **Figure S1**.

To determine the biological function of Par-4, we generated C57BL/6 Par-4 whole-body knockout (Par-4^-/-^) mice. Loss of Par-4 in these mice was confirmed by genotyping and western blot analyses (**Figures S1A–C**). Both male and female Par-4^-/-^ mice showed weight gain on normal chow diet beginning at 3-4 months of age when compared to age and gender-matched Par-4^+/+^ controls (**Figure 1A**).

Visual observation indicated subcutaneous and visceral fat accumulation in Par-4^-/-^ mice compared to Par-4^+/+^ mice. Echo-MRI analyses further verified quantitative increase in total fat in the Par-4^-/-^ mice (**Figure 1B**). Examination of the adipose tissues indicated adipocyte hypertrophy in Par-4^-/-^ mice starting after 3 months of age (**Figure 1C**). Together, these findings indicated that Par-4 loss was associated with weight gain, hypertrophic adipocytes, and fat mass accumulation in adult mice after 3 months of age on standard chow diet.

As obesity is often associated with hepatic steatosis, we examined the liver from Par-4^-/-^ mice at various age groups. Par-4^-/-^ mice did not exhibit any hepatic changes at 3 months of age, but beginning at about 6 months, the livers from Par-4^-/-^ mice had significantly larger weights and showed increased steatosis relative to the livers from Par-4^+/+^ mice (**Figure S1D**). As obesity is a risk factor for metabolic complications, such as type 2 diabetes (30, 31), we sought to assess glucose homeostasis before and after the onset of obesity. Oral glucose tolerance tests indicated that, at 3 months, glucose and insulin levels of Par-4 knockout mice were comparable to Par-4^+/+^ mice (**Figures S1E, F**). However, at 6 months, although glucose levels were controlled in Par-4^-/-^ mice, insulin levels were significantly higher in Par-4^-/-^ mice when compared to Par-4^+/+^ control mice (**Figures S1E, F**). These data suggest that more insulin is required to maintain normal glucose excursions in the Par-4^-/-^ mice after glucose challenge, suggesting insulin resistance after the mice became obese. Unlike the Par-4^-/-^ mice, the control mice (i.e., Par-4^+/+^ for wild-type C57BL6 mice, Par-4^fl/fl^, Par-4^kitg/tg^ and Par-4^fl/fl^ Par-4Ki^tg/tg^) did not show significant differences in body weight after 4 months (**Figure S1G**, and also see **Figure S8E**). Par-4 heterozygous knockout mice showed a trend toward obesity, but the differences were not statistically significant (**Figure S1H**). Moreover, steady-state plasma levels of triglycerides (TGs) were lower in Par-4 homozygous knockout mice relative to Par-4^+/+^ mice (**Figure S1I**), consistent with increased storage of lipids in their adipocytes resulting in increased fat mass. Together, these findings suggest that loss of Par-4 leads to fat accumulation, adipocyte hypertrophy, and obesity that is secondarily associated with hepatic steatosis and insulin resistance.

### Par-4 Expression Is Lower in Obese Humans

**Figure 2:**
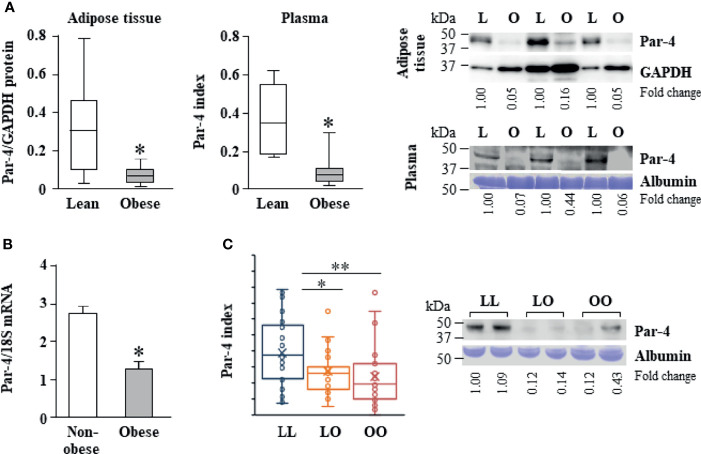
Reduced Par-4 expression in obese humans. **(A)** Reduced Par-4 protein levels in the adipose tissue and plasma of obese individuals. Abdominal adipose tissue protein extracts or plasma from lean and obese age-matched males (n=10) was subjected to western blot analysis for Par-4. The Par-4 levels in adipose tissue were normalized to GAPDH levels. Par-4 levels in the plasma were normalized to albumin levels in Coomassie blue gels. Par-4 index was calculated as a ratio of Par-4 to albumin in each sample. Box and whiskers plots for Par-4 in lean or obese subjects and western blots representative of lower expression of Par-4 in obese (O) versus lean (L) individuals are shown. **(B)** Reduced Par-4 mRNA levels in adipose tissue of obese individuals. Abdominal adipose tissue from obese and age- and gender-matched non-obese human subjects (n=10 in each group) was subjected to qPCR analysis for Par-4 and 18S rRNA. Par-4 levels were normalized to 18S rRNA levels. **(C)** Reduced Par-4 protein levels in the plasma of lean individuals that became obese after 16.5 ± 1.5 years follow-up. Plasma from lean individuals that remained lean (LL, n=37), lean individuals that became obese (LO, n=31) and obese individuals that remained obese (OO, n=34) was subjected to western blot analysis for Par-4. Par-4 levels in the plasma were normalized to albumin levels in Coomassie blue gels (Par-4 index). Box and whiskers plots showing Par-4 and western blots representative of Par-4 levels in LL, LO and OO individuals are shown. *P < 0.05, **P < 0.01 by the Student’s *t*-test with Bonferroni method for multiple comparisons adjustment. Also see **Figures S2, S10**, and **Table S1** for additional information on study participants.

As Par-4 loss in mice resulted in obesity, we determined the levels of Par-4 in visceral adipose tissues from obese (Body Mass Index [BMI]>30 kg/m^2^) and lean (BMI<25 kg/m^2^) individuals in Kentucky. Western blot analysis of adipose tissues and plasma levels of Par-4 indicated that obese human subjects exhibited lower tissue levels, as well as circulating plasma levels of Par-4 relative to lean human subjects (**Figure 2A**). Moreover, quantitative Polymerase Chain Reaction (qPCR) indicated that adipose tissues from obese individuals exhibited lower levels of Par-4 RNA relative to adipose tissues from non-obese individuals in Kentucky (**Figure 2B**), implying Par-4 regulation at the level of gene expression in obese individuals. These findings indicate that lower baseline levels of Par-4 are associated with obesity in human subjects.

These findings on lower Par-4 expression in obese individuals relative to lean individuals prompted us to assess the possible role of Par-4 in development of obesity in humans. Fasting levels of Par-4 in the plasma collected at baseline were analyzed in middle-aged subjects of the Malmö Diet and Cancer Study Cardiovascular Cohort (MDC-CC), a prospective population-based cohort study in Sweden with baseline exam in 1991-1994 and re-examination after an average follow-up time of 16.5 ± 1.5 years (“Minisymposium: The Malmö Diet and Cancer Study. Design, Biological Bank and Biomarker Programme. 23 October 1991, Malmo, Sweden.” 1993; 29, 32). Subjects who were lean at baseline (BMI<25 kg/m^2^) and developed obesity during follow-up (LO) had significantly lower baseline Par-4 protein levels than lean subjects who remained lean (LL) (**Figure 2C**). Lean subjects who remained lean during the follow-up time (LL) showed significantly higher baseline plasma levels of Par-4 compared to individuals who were obese and remained obese (OO) during the same study period (**Figure 2C**). The base population, as well as the methods of tissue collection, processing and storage was exactly the same for the cohort study participants in Sweden presented in **Figure 2C**. We further examined the association between baseline Par-4 levels and development of obesity among all lean individuals at baseline adjusting for age, sex, physical activity and energy intake. We dichotomized baseline Par-4 based on the median (Par-4-low = Par-4 < median; Par-4-high = Par-4 ≥ median) calculated from all lean individuals at baseline to evaluate the ability of Par-4 index to predict obesity. After adjusting for age, sex, physical activity and energy intake, the likelihood of being obesity-resistant was significantly higher for Par-4-high group compared to Par-4-low group (P = 0.031, odds ratio = 5.25, and 95% confidence interval = 1.16 to 23.73 based on a logistic regression model). Collectively, these studies indicate that lower Par-4 in human subjects is associated with an obese phenotype and the development of obesity.

### Par-4 Knockout in Adipocytes, but Not in Hepatocytes, Produces Obese Mice

**Figure 3:**
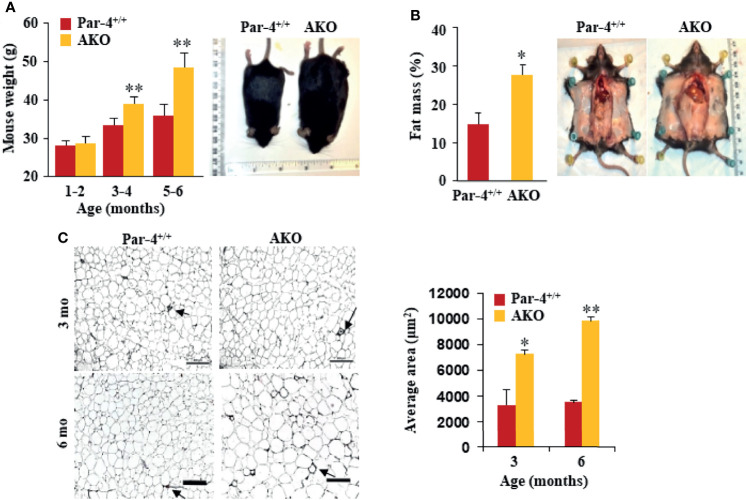
AKO mice show fat mass accumulation and obesity. **(A)** AKO mice are significantly heavier than Par-4^+/+^ mice. Body weights of age-matched male mice (1-6 months, n=6 per group) and representative images of 6-month-old male mice. **(B)** AKO mice show increased fat mass accumulation relative to Par-4^+/+^ mice. Echo-MRI analyses were carried out on 6-month-old male mice (n=5-8); representative images are shown. **(C)** AKO mice show adipocyte hypertrophy relative to Par-4^+/+^ mice. Visceral adipose tissue from 3-month-old or 6-month-old mice (n=3) was subjected to H&E staining and adipocyte cell size was quantified. Visceral fat from Par-4^+/+^ and AKO mice was also subjected to IHC for F4/80 macrophage marker. Arrows point to crown-like structures (more details, see S9E). Mean ± SEM, *P < 0.05, **P < 0.01, by the Student’s *t*-test or with Bonferroni method adjustment for multiple comparisons. Also see **Figure S3**.

Given the importance of the liver and adipose tissue in lipid synthesis and storage, respectively, we sought to determine whether Par-4 loss in these tissues contributed to the obese phenotype noted in whole-body Par-4 knockout mice. We therefore generated Par-4 hepatocyte knockout (HKO) and Par-4 adipocyte knockout (AKO) mice (**Figures S3A, B**). Similar to our findings in Par-4^-/-^ mice, AKO mice showed weight gain on a chow diet relative to Par-4^+/+^ mice, beginning at 3-4 months after birth (**Figure 3A**), and Echo-MRI analyses indicated increased fat accumulation in AKO mice (**Figure 3B**). Moreover, adipocytes from AKO mice were significantly larger than adipocytes from control mice beginning at 3 months (**Figure 3C**). On the other hand, HKO mice did not show weight gain or fat accumulation when compared to Par-4^+/+^ mice (**Figures S3C, D**). These findings indicate that Par-4 loss in adipocytes but not in the hepatocytes is sufficient to produce hypertrophic obesity in mice.

Next, we performed glucose tolerance tests to assess glucose homeostasis before and after onset of obesity. Glucose and insulin levels were similar in 3-month-old AKO and Par-4^+/+^ mice but were remarkably elevated in the AKO mice at 6 months (**Figures S3E, F**). Similar to the findings in Par-4^-/-^ mice, steady-state levels of TGs were lower in AKO mice relative to Par-4^+/+^ mice (**Figure S3G**). These data are indicative of worse glucose tolerance and insulin resistance that occur after the onset of obesity in AKO mice.

### AKO Mice Show Increased Intestinal Triglyceride Absorption

To determine the metabolic and behavioral features associated with the loss of Par-4 in both whole-body and adipocyte-specific knockout mice, we placed Par-4^-/-^, AKO and Par-4^+/+^ mice on a standard chow diet in metabolic chambers for indirect calorimetric measurements. When compared to Par-4^+/+^ mice, neither Par-4^-/-^ nor AKO mice showed any changes in food consumption, physical activity, energy expenditure or respiratory exchange ratio (RER) (**Figures S4A, C**).

**Figure 4:**
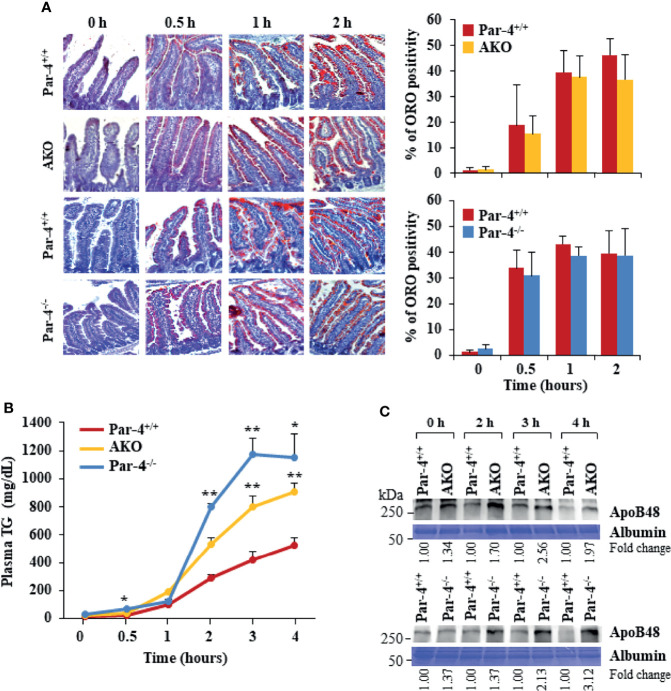
AKO and Par-4 mice show increased intestinal triglyceride absorption after a fat load. **(A)** Intestinal triglyceride accumulation appears similar in Par-4^-/-,^ AKO, and Par-4^+/+^ mice. After overnight fasting, Par-4^-/-^, AKO and Par-4^+/+^ mice (n=3) were subjected to an oral load of olive oil. Representative images of proximal intestines collected at the indicated time intervals after gavage and subjected to ORO staining (left panels). ORO staining was quantified in the enterocytes (right panels). Magnification, 10x. **(B)** Triglyceride absorption is increased in both AKO and Par-4^-/-^ mice. AKO, Par-4^-/-^ and Par-4^+/+^ male mice (n=5-9 per group) that were 6-months old were fasted overnight and injected intraperitoneally with tyloxapol 30 min before olive oil gavage to block lipase activity. Plasma was collected at the indicated time points and subjected to triglyceride analysis. **(C)** Apolipoprotein B48 secretion is increased in both AKO and Par-4^-/-^ mice. Plasma from AKO, Par-4^-/-^ and Par-4^+/+^ male mice was subjected to TG analysis and examined by western blot analysis for ApoB48. ApoB48 levels were normalized to albumin levels and fold change at each time point is shown. Mean ± SEM, *P < 0.05, **P < 0.01 by the Student’s *t*-test or with Bonferroni method adjustment for multiple comparisons. Also see **Figures S4, S11**.

As both Par-4^-/-^ and AKO mice show weight gain, fat accumulation and adipocyte hypertrophy despite an unchanged metabolic chamber profile, we sought to determine whether these mice exhibited increased intestinal uptake that can lead to obesity. Oil-red O (ORO) staining of proximal intestines, performed after feeding mice with an olive oil gavage, indicated there is no detectable difference in the amount of TG that accumulates in enterocytes in Par-4^-/-^, AKO, and Par-4^+/+^ mice (**Figure 4A**). As increased uptake of dietary fat may not result in increased accumulation of fat in enterocytes if the rate of absorption of fat from the enterocytes into the bloodstream is also simultaneously elevated, we tested the expression levels of chylomicrons in the plasma of Par-4^-/-^, AKO, and Par-4^+/+^ mice following tyloxapol pretreatment and oral gavage with olive oil. Chylomicrons are lipoprotein particles consisting of a lipid core of TG, other lipid esters, and the key monomeric protein apolipoprotein B48 (ApoB48) (33, 34). ApoB48 is expressed in an enterocyte-specific manner. Upon ingestion of fat, chylomicrons are produced by the enterocytes, then transported to the lymphatic system and released into the bloodstream. Their presence in the plasma serves as an indicator of TG and ApoB48 secretion from the intestine (35). Both TG (**Figure 4B**) and ApoB48 (**Figures 4C**, **S4D**) levels were elevated in the plasma of Par-4^-/-^ and AKO mice relative to Par-4^+/+^ mice. We used tyloxapol pretreatment to eliminate lipases in the mice and rule out the possibility of impaired TG clearance with reduced TG uptake by peripheral tissues, such as adipose tissues, or impaired recycling of the remnant chylomicron/ApoB48 to the liver. Accordingly, the findings can be interpreted to imply that there is greater absorption from enterocytes into circulation. These results indicate that Par-4 loss is associated with increased secretion of chylomicrons from the enterocytes.

### Par-4^-/-^ and AKO Mice Show Increased Expression of Acylation Stimulating Protein Associated With Fat Storage and Obesity

**Figure 5:**
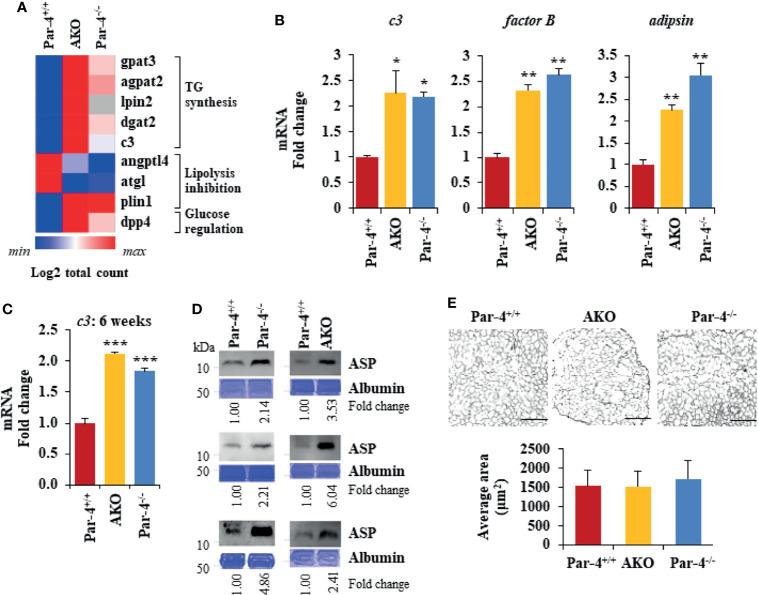
Par-4 loss results in induction of C3/ASP. **(A)** Heatmap of key genes that were up- or downregulated in 11-week old AKO or Par-4^-/-^ vs Par-4^+/+^ mouse adipose tissues. We performed RNA-Seq on AKO, Par-4^-/-^ and Par-4^+/+^ male mouse visceral adipose tissue samples (n=3 per group). The RNA used for RNA-Seq was prepared from 11-week old mice before they were impacted by major alterations in their adipose tissue or obesity. Average gene count for each group was calculated for identifying upregulated and downregulated genes. Heatmaps were generated using Morpheus software https://software.broadinstitute.org/morpheus. **(B)** *c3*, *factor B* and *adipsin* are upregulated in 3-month-old AKO and Par-4^-/-^ mice. Visceral adipose tissues from AKO, Par-4^-/-^ and Par-4^+/+^ male mice were subjected to qPCR analysis for *c3*, *factor B*, *adipsin*, and 18S rRNA. C3 levels were normalized to 18S rRNA and fold change was calculated. **(C)** *c3* RNA is elevated in 6-week-old AKO and Par-4^-/-^ mice. Visceral adipose tissues from AKO, Par-4^-/-^ and Par-4^+/+^ mice were subjected to qPCR analysis for *c3*, and 18S rRNA. C3 levels were normalized to 18S rRNA and fold change was calculated. **(D)** ASP is elevated in 6-10-week-old AKO and Par-4^-/-^ mice. Plasma from AKO, Par-4^-/-^ and Par-4^+/+^ mice was subjected to western blot analysis for ASP. ASP levels were normalized to albumin levels in parallel Coomassie blue gels and fold. **(E)** Adipocyte size is similar at 6 weeks of age. Adipose tissue from 6-week-old mice was subjected to H&E staining (scale bar, 200 μm) and adipocyte cell size was quantified. **(B, C, E)** Mean ± SEM, *P < 0.05, **P < 0.01, ***P < 0.001 by the Student’s t-test with Bonferroni method. Also see **Figures S5**, **S12**.

To determine the underlying changes in gene expression associated with Par-4 loss in Par-4^-/-^ and AKO mice, we performed RNA-Seq analysis on visceral white adipose tissues collected from 11-week-old Par-4^+/+^, Par-4^-/-^ and AKO mice. We identified 513 genes upregulated in both Par-4^-/-^ and AKO adipose tissues and 526 genes downregulated in both Par-4^-/-^ and AKO tissues, and pathway analysis indicated the predominance of genes associated with fatty acid metabolism (**Figures 5A**, **S5A, B**). In particular, key genes associated with TG synthesis and storage were upregulated in both the Par-4 knockout mice (**Figure 5A**). One of the upregulated genes was Complement *c3*. C3 protein, produced by liver, macrophages and adipocytes, generates Acylation Stimulating Protein (ASP) through a series of cleavage processes requiring factor B and adipsin (also known as factor D) produced by adipocytes (36). As ASP is causally associated with obesity (37), C3/ASP was a lead candidate for further analysis as a potential mediator of obesity upon Par-4 loss. Our validation studies indicated that relative to Par-4^+/+^ mice, Par-4^-/-^ and AKO mice showed elevated levels of C3 expression in the adipose tissues of 3-month-old mice (**Figure 5B**). As C3 proteolytic cleavage requires factor B and adipsin to produce ASP, we tested the levels of these factors in the adipose tissues from Par-4 knockout and Par-4^+/+^ mice. Both factor B and adipsin were upregulated in the adipose tissues of 3-month-old Par-4^-/-^ and AKO mice relative to Par-4^+/+^ mice (**Figure 5B**). Importantly, C3 and ASP were upregulated in the plasma of 6-week-old Par-4^-/-^ and AKO mice before the onset of adipocyte hypertrophy relative to Par-4^+/+^ mice (**Figures 5C–E**), implying that C3/ASP were elevated before the onset of obesity at 3 months. Importantly, C3 mRNA levels were also upregulated in Par-4^-/-^ mouse embryonic fibroblasts (MEFs) when compared to Par-4^+/+^ MEFs (**Figure S5C**). Together, these findings indicate an inverse relationship between Par-4 and C3/ASP.

### Obesity in Par-4 Knockout Mice Is Dependent on C3/ASP

**Figure 6:**
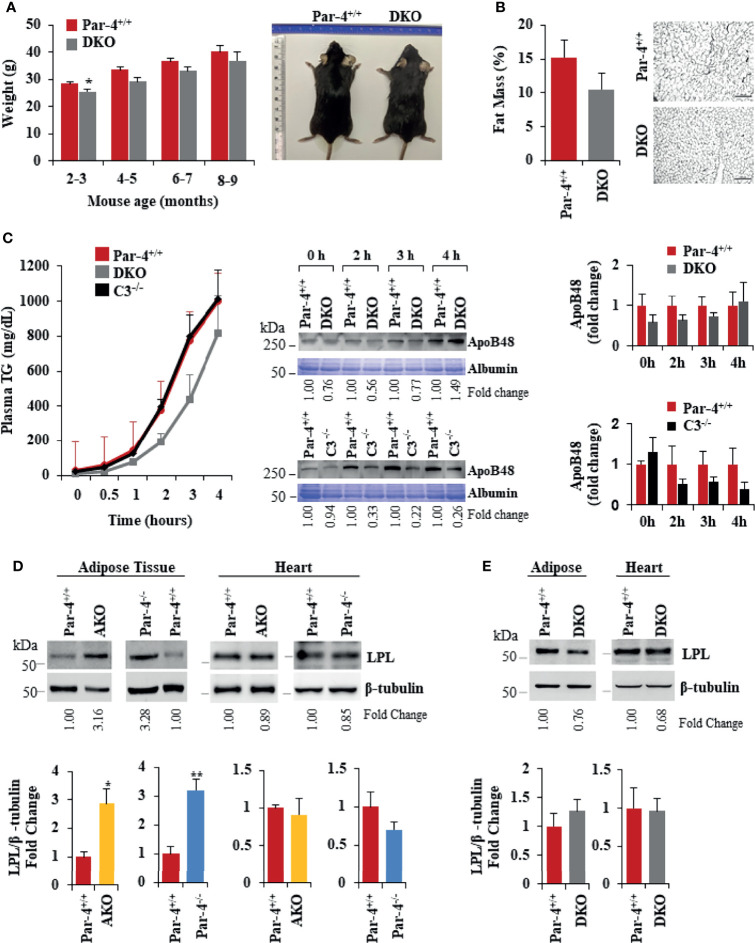
Obesity in Par-4^-/-^ mice is rescued by deletion of C3. (A) Par-4 and C3 double knockout (DKO) mice are not obese. Weight of age-matched DKO (n=13, 10, 8 and 4 for each time point respectively) and Par-4^+/+^ (n=27, 16, 9, 5 for each time point respectively) male mice was determined over the course of 7 months; representative images of Par-4^+/+^ and DKO at 5-6 months are shown. **(B)** DKO mice do not show increased fat accumulation. Six-month-old DKO (n=8) and Par-4^+/+^ (n=7) male mice were examined for fat mass by Echo-MRI and adipocyte hypertrophy. **(C)** Triglyceride absorption is similar in DKO, C3KO and Par-4^+/+^ mice. DKO (n=5-6), C3^-/-^ (n=4) and Par-4^+/+^ (n=11) male mice were fasted overnight and injected intraperitoneally with tyloxapol, 30 min before oral gavage of olive oil. Plasma was collected at the indicated time intervals after fat load, and triglycerides were quantified. Plasma was also subjected to western blot analysis for ApoB48. ApoB48 levels in the plasma were normalized to albumin in corresponding Coomassie blue gels, and fold change at each time point is shown (n=4). **(D)** LPL is upregulated in the adipose tissue of AKO and Par-4^-/-^ mice. LPL protein levels were determined by western blot analysis in visceral adipose tissue and heart from AKO, Par-4^-/-^ and Par-4^+/+^ mice (n=3). **(E)** LPL is not increased in the adipose tissue of DKO mice. LPL protein levels were determined by western blot analysis in visceral adipose tissue and heart from Par-4^+/+^ (n=4) and DKO (n= 4) male mice. LPL levels were normalized to β-tubulin levels and fold change was calculated. Mean ± SEM, *P < 0.05, **P < 0.01, by the Student’s *t*-test. Also see **Figures S6**, **S13**.

To determine whether C3/ASP upregulation was functionally relevant in the obese phenotype of Par-4 knockout mice, we crossed C3^-/-^ mice with Par-4^-/-^ mice and tested the C3/Par-4 double knockout (DKO) mice (**Figure S6A**) for obesity on standard chow diet. DKO mice did not show weight gain, adipocyte hypertrophy or fat mass accumulation relative to Par-4^+/+^ mice (**Figures 6A, B**). Moreover, chylomicron secretion measured by TG and ApoB48 levels in the plasma after olive oil gavage was not elevated in DKO mice relative to Par-4^+/+^ mice (**Figure 6C**).

As elevated levels of ASP promote an increase in lipoprotein lipase (LPL) (38), we determined LPL protein levels in the adipose tissue and heart muscle of Par-4^+/+^, Par-4^-/-^ and AKO mice. LPL is the rate-limiting enzyme that induces the clearance of fatty acids from circulation by hydrolysis of TG-rich lipoproteins and the uptake of derived fatty acids (39). LPL is expressed by the skeletal muscles, including heart muscle, and white adipose tissue. In skeletal muscles, fatty acids are largely oxidized, whereas in the adipose tissue they are esterified and stored as TGs (40). LPL levels were elevated in the adipose tissues but not the heart of Par-4^-/-^ and AKO mice relative to the corresponding tissues of Par-4^+/+^ mice (**Figure 6D**). In contrast, LPL levels remained unchanged in the heart and in the adipose tissues of DKO mice relative to Par-4^+/+^ mice (**Figure 6E**). As LPL in the adipose tissues is associated with clearance of TGs from circulation and storage in adipocytes, these findings are consistent with increased fat storage and hypertrophic obesity in Par-4^-/-^ and AKO mice and reversal of this phenotype in DKO mice.

### C3 Induction Following Par-4 Loss Is Regulated by p53

**Figure 7:**
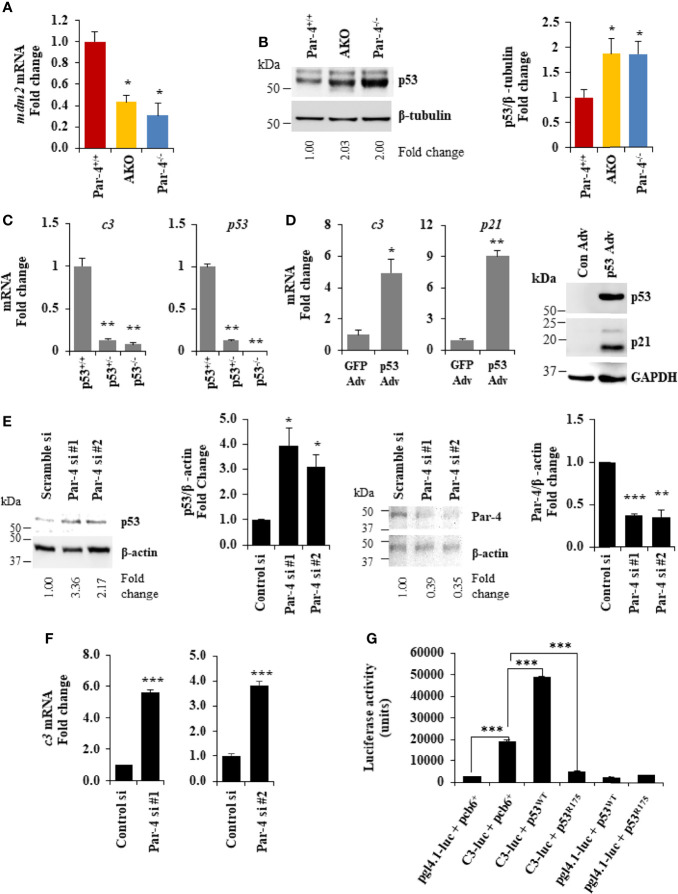
Par-4 regulates C3 *via* p53. **(A)** *mdm2* is downregulated in the adipose tissue of AKO and Par-4^-/-^ mice. RNA from visceral adipose tissues from 3-month-old AKO, Par-4^-/-^ and Par-4^+/+^ mice were subjected to qPCR analysis for *mdm2* and 18S rRNA. *mdm2* levels were normalized to 18S and fold change was calculated. **(B)** p53 is upregulated in the adipose tissue of AKO and Par-4^-/-^ mice. Visceral adipose tissues from 6-week-old AKO, Par-4^-/-^ and Par-4^+/+^ mice (n=4) were subjected to western blot analysis for p53 and β-tubulin. p53 levels were normalized to β-tubulin and fold change is shown. p53 was induced in AKO and Par-4^-/-^. **(C)** C3 is downregulated in the p53-null MEFs. RNA from p53^+/+^, p53^+/-^ and p53^-/-^ MEFs was subjected to qPCR analysis for *c3*, *p53* and 18S rRNA. C3 and p53 levels were normalized to 18S rRNA and fold change was calculated (left panel). p53 status in the three MEFs lines was confirmed (right panel). **(D)** C3 is induced with p53 rescue in p53-null MEFs. p53-null (p53^-/-)^ MEFs were transduced with either control GFP adenovirus (Adv) or p53 adenovirus. RNA was collected 24h after infection and subjected to qPCR analysis for *c3*, *p21* and 18S rRNA (left and middle panel). Whole-cell extracts from the adenovirus infected cells were subjected to western blot analysis for p53 and p21 using GAPDH as a loading control (right panel). **(E)** p53 is upregulated in MEFs. WT MEFs were transfected with either control siRNA or two different Par-4 siRNA (#1 and #2 from Dharmacon; n=3). Whole-cell extracts from transfected cells were collected after 24h of transfection and subjected to western blot analysis for p53 (left panel) and Par-4 (right panel) using β-actin as a loading control. **(F)** *c3* RNA is upregulated in WT MEFs knocked down for Par-4. RNA from WT MEFs transfected with either scramble siRNA or two different Par-4 siRNA (si Par-4 #1, left panel; siPar-4 #2, right panel) was collected. RNA was subjected to RT-qPCR for c3 and 18S. Fold change was calculated using 18S as housekeeping control. **(G)** p53 induces the *c3* promoter. p53^+/+^ MEFs were transiently co-transfected with: (1) C3-promoter reporter (TK-luc containing c3 promoter region; C3-luc), p53^WT^ and β- galactosidase expression construct; (2) C3-luc, p53^R175^ and β-galactosidase construct; (3) pgl4-1 control luciferase construct, p53^WT^ and β-galactosidase construct; and (4) C3-luc, p53^R175^ and β- galactosidase construct. Luciferase activity was determined after 24h and normalized to corresponding β-galactosidase activity. **(A–C, E, F)** Mean ± SEM, *adjusted *P* < 0.05, **P < 0.01, ***P < 0.001 by the Student’s t-test or with Bonferroni method. Mean (Relative luciferase activity units) ± SEM, ***P < 0.001 by the Student’s t-test or with Bonferroni method adjustment for multiple comparisons. Mean ± SEM, *P < 0.05, **P < 0.01 by the Student’s *t*-test or with Bonferroni method adjustment for multiple comparisons. Also see **Figures S7**, **S14**.

As C3 induction plays a functional role in the obese phenotype of Par-4 knockout mice, we determined the mechanism by which Par-4 regulates C3 expression. Par-4 has been previously shown to modulate gene expression by cooperating with other transcription factors as a transcriptional co-repressor (41, 42) or transcriptional co-activator (43). To identify downstream genes that are regulated by Par-4 in adipocytes, we therefore performed ChIP-Seq analysis with the Par-4 antibody using 3T3-L1 cell cultures. These experiments led to the identification of several genes, including *mdm2* encoding the ubiquitin ligase MDM2 as a potential target of Par-4 (**Table S2**). As MDM2 regulates p53 activity, and as p53 induction is associated with obesity (44–46), we validated MDM2 and p53 regulation by Par-4. As seen in **Figure 7A**, *mdm2* expression was downregulated in the adipose tissues of Par-4^-/-^ and AKO mice relative to Par-4^+/+^ mice. Consistently, expression of p53 (**Figure 7B**; blot with secondary antibody only is shown in **Figure S7A**), as well as its authentic target p21 (**Figure S7B**), was upregulated in Par-4^-/-^ and AKO mice relative to Par-4^+/+^ mice. To further examine whether p53 regulates *c3*, we tested the expression of *c3* RNA in MEFs from p53 wild type mice (p53^+/+^), p53 homozygous (p53^-/-^) and heterozygous (p53^+/-^) knockout mice. *c3* expression was downmodulated in p53^-/-^ and p53^+/-^ MEFs relative to p53^+/+^ MEFs (**Figure 7C**). On the other hand, reintroduction of p53 in p53^-/-^ MEFs caused induction of *c3* and *p21* expression (**Figure 7D**). To determine the effects of acute Par-4 knockdown in MEFs, we used two different siRNA duplexes against Par-4. As seen in **Figure 7E** (right panel), downregulation of endogenous Par-4, resulted in inhibition of *mdm2* (**Figure S7C**), induction of p53 (**Figure 7E** , left panel) and *c3* (**Figure 7F**). Finally, we tested whether p53 regulates the expression of the C3 promoter in luciferase-reporter assays. As seen in **Figure 7G**, wild type p53 but not a mutant of p53, induced the *c3* promoter. Together, these findings indicate that Par-4 loss results in loss of *mdm2* that leads to p53 activation, and that p53 induces the expression of *c3* at the promoter level.

### Obesity in AKO Mice Is Rescued by Par-4 Knock-In Into Adipocytes

**Figure 8:**
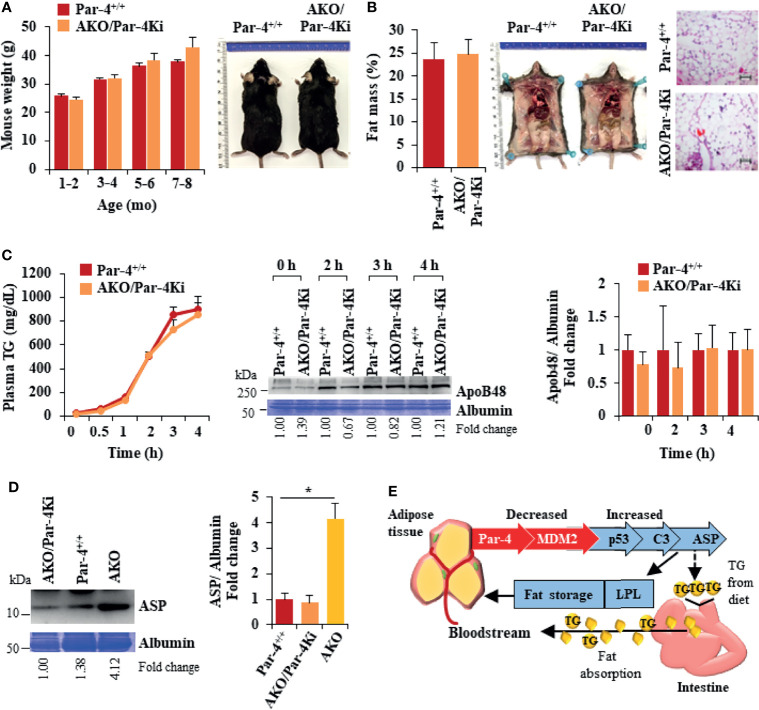
Obesity in AKO mice is rescued by Par-4 knock-in into adipocytes. **(A)** AKO/Par-4Ki mice are not obese. Body weights of age-matched male AKO/Par-4Ki mice (n=9, 17, 11, 6) and control mice (n=6, 24, 17, 25) were collected for nine months. Representative images of 7-month-old male mice are shown. **(B)** Fat accumulation in AKO/Par-4Ki mice is comparable to controls. Echo-MRI analyses were carried out on 5-7-month-old AKO/Par-4Ki (n=7) and control (n=11) male mice; representative images of 7-month-old male mice are shown. **(C)** Triglyceride absorption is similar in AKO/Par-4Ki and Par-4^+/+^ mice. AKO/Par-4Ki (n=8), and Par-4^+/+^ (n=9) mice were fasted overnight and injected intraperitoneally with tyloxapol, 30 min before oral gavage of olive oil. Plasma was collected at the indicated time intervals after fat load, and triglycerides were quantified. Plasma was also subjected to western blot analysis for ApoB48. ApoB48 levels in the plasma were normalized to albumin in corresponding Coomassie blue gels, and fold change at each time point are shown (n=4). **(D)** ASP is not upregulated in AKO/Par-4Ki. Plasma from AKO/Par-4Ki (n=3), AKO (n=3) and Par-4^+/+^ (n=3) male mice was subjected to western blot analysis for ASP. ASP levels in the plasma were normalized to albumin in corresponding Coomassie blue gels, and representative fold change is shown. **(E)** Par-4 loss is associated with induction of C3 and ASP, due to p53 upregulation. Par-4 loss results in downregulation of the ubiquitin ligase MDM2. This leads to p53 activation and induction of complement factor C3. Further induction of factor B and adipsin results in proteolytic cleavage of C3 protein to ASP. Elevated C3/ASP were associated with increased absorption of chylomicrons (TGs and ApoB48) from the intestine into the bloodstream and increased LPL for fat storage in the adipocytes. These latter effects of Par-4 loss were reversed by knocking out C3/ASP in Par-4 knockout mice. Thus, Par-4 loss results in fat accumulation, adipocyte hypertrophy and obesity in a C3/ASP dependent manner. Consistently, our cohort study indicated that low levels of Par-4 may predict future obesity. Mean ± SEM, *P < 0.05 by the Student’s *t*-test with Bonferroni method adjustment for multiple comparisons. Also see **Figures S8**, **S15**.

To confirm that obesity induced by Par-4 loss in adipocytes was primarily associated with Par-4 function and was not a consequence of unrelated downstream events in mice, we tested whether obesity in AKO could be reversed by re-expression of Par-4 in adipocytes. Accordingly, Par-4Ki^tg/tg^ mice were crossed with AKO mice for adipocyte specific re-expression of Par-4 as indicated in Materials and Methods section and **Figures S8A, S8B**. Induction of Par-4 in adipocytes was confirmed by Western blot analysis of adipose tissue (**Figure S8C**). Par-4 re-expressing AKO (AKO/Par-4Ki) mice showed similar weights and fat mass accumulation as Par-4^+/+^ mice (**Figures 8A, B**; also see **Figure S8D, E**). Moreover, TG absorption and ApoB48 levels were similar in AKO/Par-4Ki and Par-4^+/+^ mice (**Figure 8C**). Unlike AKO mice that showed increased levels of ASP and LPL in their plasma relative to Par-4^+/+^ mice, AKO/Par-4Ki showed p53, p21 and ASP, as well as LPL levels similar to those of Par-4^+/+^ mice (**Figures 8D**, **S8F**). AKO/Par-4Ki also showed glucose and insulin levels similar to those of Par-4^+/+^ mice (**Figure S8G**). Together, these findings indicate that re-expression of Par-4 in adipocytes reverses p53 induction, ASP upregulation, LPL elevation, lipid absorption, fat mass accumulation and weight gain, as well as secondary increase in glucose and insulin levels noted in AKO mice.

## Discussion

The present study uncovered an unpredicted physiological function of Par-4. Conventional Par-4 knockout mice were overweight and accumulated significantly higher visceral and subcutaneous adipose tissue relative to control mice on standard chow. Adipocyte hypertrophy began at about three months after birth. Deletion of Par-4 in mouse adipocytes, but not in the mouse hepatocytes, recapitulated the obese phenotype of Par-4 whole-body knockout mice. Hepatic steatosis and hyperinsulinemia occurred as a secondary complication of obesity. Weight gain, fat mass accumulation as well as most other obesity related features of AKO mice were generally similar to that of Par-4^-/-^ mice. Moreover, obesity in AKO mice was reversed by re-expression of Par-4 in adipocytes, implying that Par-4 in adipocytes regulated the obese phenotype in mice. The relevance of these observations in human obesity was substantiated by lower expression of Par-4 in the adipose tissues from obese individuals relative to lean individuals. Most importantly, our cohort study indicated that Par-4 expression in plasma was lower in lean individuals who developed obesity during an average follow-up time of 16.5 ± 1.5 years relative to those who remained lean during this time period. Obese subjects who remained obese during this follow-up time also showed significantly lower levels of Par-4 compared to lean individuals who remained lean. Both intracellular transcriptional regulatory effects and apoptotic functions of extracellular (secreted) Par-4 have been reported in literature (1). Par-4 is secreted by normal fibroblast and epithelial cell cultures, and secreted Par-4 does not induce growth inhibition in normal cells (13), but the tissue types *in vivo* that secrete Par-4 or the role of secreted Par-4 in modulating obesity is not known. Our future studies will test whether Par-4 expression in plasma is functionally involved in regulation of obesity or serves only as a biomarker to predict obesity. In view of our observation that Par-4 was lost prior to the onset of obesity in Par-4^-/-^ and AKO mice, these findings imply that Par-4 loss is a predictor of future obesity.

### Par-4^-/-^ and AKO Mice Display Obesity on Standard Chow

Unlike most genetically engineered mouse models that develop obesity on high fat diet or due to increased food consumption and/or decreased energy expenditure (47–49), our studies indicated that Par-4^-/-^ and AKO mice displayed obesity on standard chow diet. Metabolic and behavioral studies indicated that the Par-4 knockout mice did not show increased food consumption, decreased physical activity, or energy expenditure relative to control mice. Leptin levels were higher and adiponectin levels were lower in Par-4 knockout mice relative to Par-4^+/+^ mice, consistent with the obese phenotype (**Figure S9A**). Lean mass was consistent with the obese phenotype in the Par-4^-/-^ and AKO mice, and with reversal of obesity in the DKO and AKO/Par-4Ki mice (**Figure S9B**). Both male and female Par-4 knockout mice exhibited obesity, implying that the phenotype was not controlled by sex-specific hormonal mechanisms. This phenotype of Par-4 knockout mice is reminiscent of individuals who are unable to control obesity despite controlling their diet.

In studies to determine the underlying cause of obesity in Par-4 knockout mice, we noted that Par-4 loss is associated with increased TG levels in the plasma of mice that were fed olive oil by oral gavage. ORO staining of intestinal segments did not discern elevated levels of fat accumulation in Par-4 knockout and control mice. Increased uptake may result in increased absorption, yet increased uptake may not be discernable by ORO staining due to saturation of the enterocytes with fat. We therefore examined whether absorption of TGs from the intestine into blood circulation was enhanced by Par-4 loss. These experiments used tyloxapol pretreatment, to inhibit lipases, followed by oral gavage with olive oil to determine TG and chylomicron levels in circulation. We noted elevated levels of TG in the plasma of both Par-4^-/-^ and AKO mice relative to Par-4^+/+^ mice that were fed olive oil. ApoB48, which is produced in the intestine and required for chylomicron secretion, was also elevated in circulation of the Par-4^-/-^ and AKO mice following tyloxapol pretreatment and olive oil gavage. As chylomicrons are comprised of TGs and ApoB48, and as ApoB48 is expressed in an enterocyte-specific manner, the presence of elevated TGs and ApoB48 in the plasma serves as an indicator of increased TG and ApoB48 absorption from the intestine (35). The use of tyloxapol pretreatment allowed us to inhibit lipases in the mice, and therefore impaired TG clearance or TG uptake by peripheral tissues, such as adipose tissues, or impaired recycling of the remnant chylomicron/ApoB48 to the liver was ruled out as a possible cause of elevated TGs and ApoB48. Our findings indicating reversal of TG absorption upon Par-4 re-expression in the adipocytes of AKO mice further suggest that elevated levels of adipose tissue fat are associated with increased intestinal TG absorption in Par-4^-/-^ and AKO mice. However, as the obese phenotype in Par-4^-/-^ and AKO mice was robust on chow diet, which is fat-poor and carbohydrate rich diet, it is possible that more carbohydrate is converted to fat for storage. This observation is consistent with elevated expression of genes such as *fasn* and *scd2* (see **Figure S5**) associated with *de novo* lipogenesis. Therefore, the conversion of more carbohydrates into fat and storage in adipocytes, that may be an additional reason for obesity in the chow fed animals, needs to be addressed in the future studies. Similarly, future studies will use isotope-labeled lipids to monitor lipid uptake and oxidation in adipose tissues and other metabolic tissues including heart, muscle and liver. Moreover, it is necessary to compare food intake, energy expenditure, and lipid excretion on chow and high-fat diet in Par-4 knockout and AKO mice relative to Par-4^+/+^ mice.

### Par-4^-/-^ and AKO Mice Display Increased C3/ASP Expression in Adipocytes That Is Causally Linked to Elevated LPL, Fat Storage and Obesity

We reasoned that, as Par-4 knockout mice show similar levels of food intake and energy expenditure as the control mice, increased fat storage in the adipose tissue and obesity must be a consequence of gene alterations in adipocytes that result in uptake of circulating TGs for storage. We therefore performed an unbiased screen for differential gene expression in adipose tissues from Par-4^-/-^ or AKO mice relative to Par-4^+/+^ mice. These studies identified several genes associated with TG synthesis or storage (*gpat3*, *agpat2, lpin2, dgat2, c3)*, inhibition of intracellular lipolysis in adipocytes (*angptl4, atgl*), and regulation of glucose homeostasis (*dpp4*).

In particular, C3 and its proteolytic product ASP are causally associated with fat storage and obesity (37). C3 is proteolytically processed by binding to factor B and the adipokine adipsin specifically in the adipose tissue. Importantly, ASP is upregulated in obesity and is involved in lipid clearance from circulation. The primary effect of ASP is the promotion of fat storage in adipocytes by elevating intracellular diacylglycerol acyltransferase (DGAT) activity (50), which catalyzes the formation of triglycerides from diacylglycerol and acyl-CoA, and GLUT4 translocation, which drives extracellular fatty acid and glucose uptake into cells. ASP also inhibits intracellular lipolysis, and injection of ASP increases fat storage, and neutralization of ASP with an antibody inhibits fat storage (51, 52). As expected, C3 knockout mice show ASP-deficiency, and the protective potential of ASP-deficiency against obesity was confirmed in *ob*/*ob*-C3^-/-^ double knockout mice (53). Together, these observations imply that ASP is a unique factor that promotes obesity and apparently links the complement arm of the immune system to metabolism.

In adipocytes, ASP action is mediated through activation of downstream kinases (54), but the upstream signals responsible for upregulation of the C3/ASP pathway have not been delineated. Our studies indicate that C3 and ASP are expressed at higher levels at 6 weeks (**Figures 5C, D**), before the onset of adipocyte hypertrophy noted after 3 months in Par-4^-/-^ and AKO mice. C3 is produced mainly by hepatocytes and adipose tissue, but C3 is converted to ASP in only the adipose tissue due to adipocyte-specific expression of adipsin. Our hepatocyte Par-4 knockout mice do not show C3 upregulation (**Figure S9C**) and are not obese. These observations implied that Par-4 regulates downstream expression of C3 in a tissue-specific manner and provided the rationale for determining whether C3/ASP elevation is functionally associated with adipocyte hypertrophy, increased visceral fat storage and obesity in Par-4 knockout mice. Remarkably, deletion of C3 in Par-4^-/-^ mice resulted in reversal of adipocyte hypertrophy, fat mass accumulation and obesity. Furthermore, C3-Par-4 double knockout mice failed to show elevated absorption of TGs or ApoB48 from the intestine in response to olive oil gavage, implying a link between ASP associated adipocyte hypertrophy and enhanced TG absorption in mice lacking Par-4. Together with these observations, reversal of the obese phenotype in DKO mice is a most likely consequence of C3 loss in the adipose tissue. Given the possibility that C3 knockout may have yet unidentified pleiotropic effects that “rescue” the Par-4 phenotype through non-specific mechanisms, future studies may confirm the adipocyte-specific effects of C3 by crossing AKO mice with conditional adipocyte knockout C3 mice. To avoid confusion, we clarify that a previous publication in literature is based on protease-activated receptor 4 (also called Par-4) and complement C4a (55), and does not involve prostate apoptosis response-4 (Par-4/PAWR).

ASP elevation results in increased adipocyte LPL essential for storage of fat in adipocytes (37). LPL levels in the adipose tissues, but not the heart, were elevated in Par-4^-/-^ and AKO mice relative to Par-4^+/+^ mice. These findings are consistent with increased fat storage in the visceral adipose tissue of these knockout mice. On the other hand, DKO mice failed to show elevated LPL in the adipose tissue relative to Par-4^+/+^ mice and failed to induce fat storage despite the lack of Par-4, implying that C3/ASP that was elevated in response to Par-4 loss was an essential downstream mediator of adipocyte hypertrophy, fat storage and obesity in Par-4 knockout mice. Together, our findings suggest that Par-4 loss in adipocytes results in obesity that is associated with adipocyte hypertrophy and fat accumulation in visceral adipose tissue caused by increased C3/ASP. Although LPL protein levels are expected to correlate with LPL activity, our future studies will measure LPL enzymatic activity. We hypothesize that high LPL levels and activity in Par-4^-/-^ or AKO adipose tissue act to reduce the TG levels in circulation by increasing their storage. This creates a “sink” for TGs in circulation that stimulates fat absorption. In addition, our experiments in Caco-2 cells suggest that factors secreted by the adipocytes upon Par-4 loss in Par-4^-/-^ and AKO mice may directly or indirectly regulate fat absorption (**Figure S9D**). As we were unable to detect any difference in lipid excretion in these mice, our future studies will examine whether ASP or ASP-regulated downstream factors may promote enterocyte uptake of diet-derived TGs and fatty acids in the context of Par-4 loss.

### C3 Induction Following Par-4 Loss Is Regulated by p53

Par-4 is a transcriptional co-regulator that may either co-repress or co-activate gene transcription events depending on the genetic context (41–43). ChIP-Seq analysis aimed at identifying downstream mediators of C3 induction upon Par-4 loss led to the identification and validation of MDM2 as a potential target of Par-4. We focused on the MDM2-p53 axis as a potential regulator of C3 because: (a) MDM2 is the primary ubiquitin ligase responsible for degradation of the guardian of the genome p53 protein (56), (b) p53 has been previously shown to promote obesity (44, 44, 46), and (c) and several putative p53 consensus binding sites are located in the *c3* gene promoter (EPD and JASPAR databases). Our findings indicated that Par-4 loss results in downregulation of MDM2 and activation of p53 that was also evident from p21/CDKN1A induction in mouse adipose tissues. It is noteworthy that induction of the p21/CDKN1A is associated with adipocyte hypertrophy and obesity in mice (57, 58). Moreover, p53 loss in knockout cells resulted in loss of *c3* gene expression and upregulation of p53 induced *c3* promoter and RNA expression. Together, the MDM2-p53 axis links Par-4 loss to C3 induction and obesity in Par-4 knockout mice.

### Par-4^-/-^ and AKO Mice Display Secondary Complications of Obesity

Our studies indicated that Par-4^-/-^, AKO and Par-4^+/+^ mice show similar growth characteristics, including similar weight gain in the first few months after birth. At 3 months of age, the adipose tissues from these mice did not show obvious macroscopic differences, but the adipose tissue from Par-4^-/-^ and AKO mice show hypertrophy that was obvious microscopically. Unlike adipocyte hyperplasia that is associated with adipocyte proliferation and insulin sensitivity, adipocyte hypertrophy results in secondary complications such as hepatic steatosis and insulin resistance (59, 60). Infiltration of macrophages and other immune cells is often the underlying cause of these secondary complications (61). Consistent with these observations, the adipose tissues from AKO mice showed macrophage infiltration, and began developing hyperinsulinemia at 6 months but not at 3 months after birth (**Figures S3E, S3F, S9E**). Thus, adipocyte hypertrophy associated with fat storage was a primary effect that was followed subsequently by inflammatory and metabolic features that are secondarily associated with obesity, including macrophage infiltration and hyperinsulinemia. Similarly, Par-4^-/-^ mice showed hepatic steatosis and hyperinsulinemia at 6 months but not at 3 months after birth (**Figures S1D–F**). We did not study the insulin resistance phenotype in more detail as high glucose or insulin levels occurred later as a secondary consequence of obesity. The phenotypic and gene expression changes noted above were similar for both male and female mice in all the experiments and were consistently observed in over multiple generations of mice. Together, these features indicate primary hypertrophic obesity in Par-4 knockout mice.

Excess body weight affects the release of inflammatory and pro-tumorigenic proteins that are produced by adipose tissue to promote the growth and metastatic properties of tumors (19–23). The precise relationship between the factors produced by adipocytes and cancer is, however, not fully understood. Moreover, not all obese individuals develop cancer, and although loss of tumor suppressors increase the risk of developing tumors, the timing of tumor development varies among individuals. As Par-4^-/-^ mice exhibit obesity, high glucose and insulin levels at 6 months (this study) and are reported to develop tumors in diverse tissues later as they age (9), our findings provide the groundwork for future studies to elucidate whether specific features associated with loss of Par-4 function in adipocytes not only promote obesity, but also serve as a risk factor for cancer.

In summary, the present study revealed that Par-4 expression is lower in the adipose tissue of obese human subjects relative to lean subjects and may serve as a predictor of future obesity in lean subjects. Importantly, genetic loss of Par-4 results in hypertrophic obesity in both conventional and adipocyte-conditional Par-4 knockout mice on standard chow diet. These effects of Par-4 are functionally linked to C3/ASP upregulation, increased chylomicron secretion and LPL regulation for fat storage in adipocytes (**Figure 8E**). Thus, our findings identify Par-4 as a physiological regulator of lipid metabolism and uncover an adipocyte-intestinal axis that regulates obesity. Because obesity is a predisposing factor for cancer, and because Par-4 loss is also linked to increased tumorigenesis, Par-4 restoration may be explored to overcome obesity and thereby inhibit obesity-associated cancer.

## Data Availability Statement

The data presented in the study are deposited in the NCBI GEO repository accession number GSE159147.

## Ethics Statement

The studies involving human participants were reviewed and approved by University of Kentucky IRB. Written informed consent for participation was not required for this study in accordance with the national legislation and the institutional requirements. The animal study was reviewed and approved by University of Kentucky IACUC.

## Author Contributions

NA and JS performed experiments and wrote the manuscript. SN, RB, NH, SG, TS-B, BZ, and WK performed experiments. DH, CW, YZ, BT, JL, LC, and HW analyzed the data. OM and PK provided reagents. AM, LAC, MN-K, PN, BME, and PK provided expertise and feedback. VR conceived and supervised the project. All authors contributed to the article and approved the submitted version.

## Funding

This work was supported by NIH/NCI grants R01 CA165469, R01 CA187273, and R21 CA179283 (to VR), along with R01 DK071349 and DK080327, and CTSA grant UL1 TR001998 (to PK), and R01 DK112034 (to BE). NA was supported by a scholarship (ID 13137-13-1) from Coordenação de Aperfeiçoamento Superior (CAPES), Brazil. JS was supported by NCI grant T32 CA165990 (to VR).

## Conflict of Interest

Authors TS-B and BT are employed by Loxo Oncology. VR is owner of a start-up company Parcure, LLC, in Lexington, KY, USA.

The remaining authors declare that the research was conducted in the absence of any commercial or financial relationships that could be construed as a potential conflict of interest.

## Publisher’s Note

All claims expressed in this article are solely those of the authors and do not necessarily represent those of their affiliated organizations, or those of the publisher, the editors and the reviewers. Any product that may be evaluated in this article, or claim that may be made by its manufacturer, is not guaranteed or endorsed by the publisher.
