# Supplementary material for: Tumor Suppressor Par-4 Regulates Complement Factor C3 and Obesity
Source: Front Oncol. 2022 Mar 29;12:860446. doi: 10.3389/fonc.2022.860446 (PMC9004617; doi:10.3389/fonc.2022.860446)
Supplement: Supplementary file 1 [file DataSheet_1.pdf]

# *Supplementary Material*

## SUPPLEMENTARY FIGURES

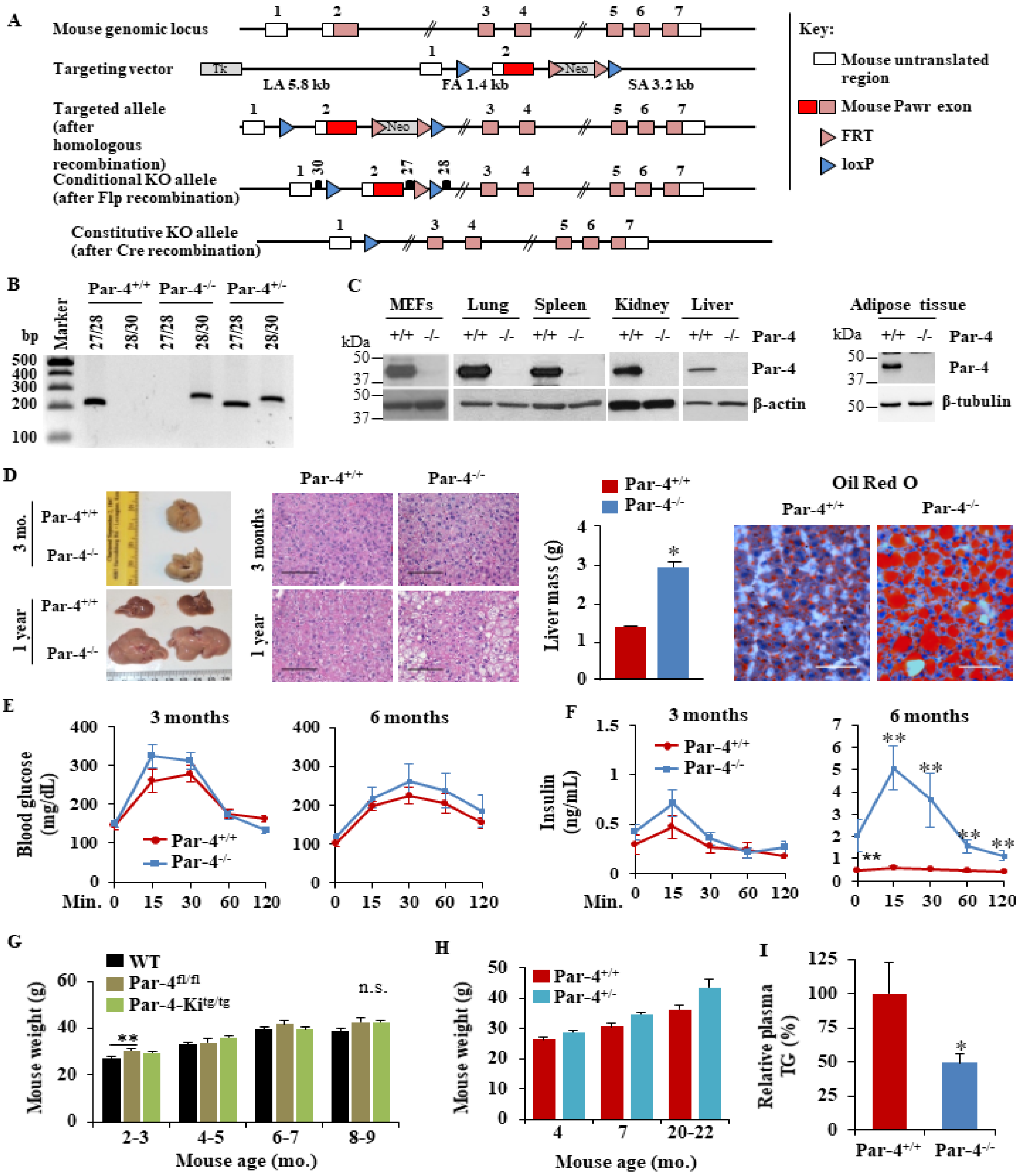


Supplementary Figure S1 (continues on next page)

**Uncut gels or larger portions of cut gels for Supplementary Figure S1C**

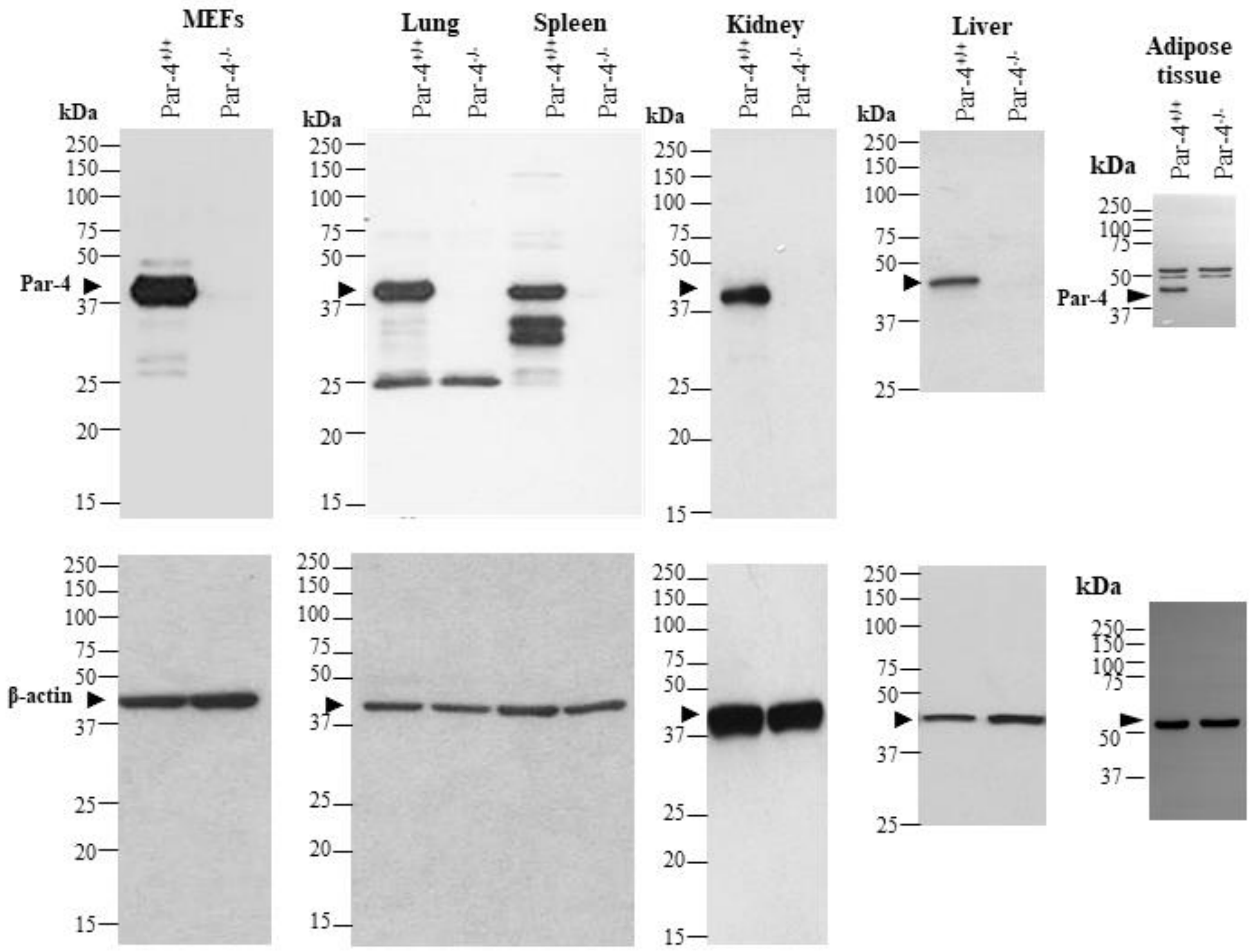


**Supplementary Figure S1 (Continued)**

**Supplementary Figure S1 (Related to Figure 1). Genotype and phenotype of genetically engineered mice. (A) Schematic representation of Par-4/PAWR targeting strategy and generation of Par-4 knockout mice**. The mouse Par-4/PAWR gene contains 7 exons (numbered) with coding (colored) regions or transcribed but non-coding (white) regions connected by introns (heavy lines). The targeting vector contains a neomycin-resistance (neo) cassette flanked by flipase (FRT) recombinase sites (pink triangles) and is placed within introns 2 and 3 of Par-4 with loxP sites (blue triangles) at both ends. This ensured targeting of exon 2, which contains the first methionine (start codon ATG). The targeted allele after homozygous recombination is shown. Conditional floxed/floxed (fl/fl) mice were generated after flipase recombination. The conditional KO mice were crossed with Rosa-26-cre to generate constitutive KO (Par-4$^{-/-}$) mice. The position of primers number 27, 28 and 30 to genotype the KO mice is indicated. The strategy was partially described by Burikhanov et al., 2014. **(B) Genotyping of constitutive KO mice.** DNA from tail clips of Par-4$^{-/-}$, Par-4$^{+/-}$ and Par-4$^{+/+}$ mice were subjected to PCR using optimized primers for the Par-4 transgene, and the resultant DNA fragments were electrophoresed on agarose gels and stained with ethidium bromide. As expected, primer set 27/28, but not primer set 28/30, detected a 213 bp fragment in Par-4$^{+/+}$ (wild type) mice; primer set 28/30, but not primer set 27/28, detected a 248 bp fragment in Par-4$^{-/-}$ mice; and primer set 27/28 and primer set 28/30 detected a 213 bp fragment and 248 bp fragment, respectively, in Par-4$^{+/-}$ mice. **(C) Western blot analysis of proteins from Par-4$^{-/-}$ and Par-4$^{+/+}$ tissues**. Protein extracts of various tissues from Par-4$^{-/-}$ and Par-4$^{+/+}$ mice were subjected to western blot analysis for Par-4 and either β-actin or β-tubulin were used as loading control. **(D) Hepatic steatosis in Par-4$^{-/-}$ mice.** Representative images of whole livers, H&E-stained liver sections, liver volumes (n=4), and Oil Red O-stained sections of 15-month-old mice livers from Par-4$^{-/-}$ and Par-4$^{+/+}$ mice are shown. Scale bar, 50 µm. **(E) Glucose levels in Par-4$^{/-}$ and Par-4$^{+/+}$ mice.** Glucose was orally administered to 3-month-old (n=4) and 6-month-old (n=8) mice maintained on standard chow, and plasma levels of glucose were determined.**(F) Insulin levels in Par-4$^{-/-}$ and Par-4$^{+/+}$ mice.** Glucose was orally administered to 3-month-old (n=4) and 6-month-old (n=8) mice maintained on standard chow, and plasma levels of insulin were determined. **(G) Weight comparison among control mice.** Weight gain of age-matched WT (n=30, 9, 25, 6), Par-4$^{fl/fl}$ (n=17, 17, 6, 7), and Par-4-Ki$^{tg/tg}$ (n=23, 13, 16, 7) control mice are similar across 9 months of follow up. **(H) Weight of age- and sex-matched Par-4$^{+/-}$ female mice relative to Par-4$^{+/+}$ counterparts.** Weight gain in female mice (n=4-8) was determined over the indicated time period. **(I) Plasma triglycerides are decreased in Par-4$^{-/-}$ mice**. Steady-state (no fasting) plasma was collected from Par-4$^{+/+}$ (n=6) and Par-4$^{-/-}$ (n=5) 11-month-old male mice. Plasma triglyceride levels were determined, and percentage decrease is shown. **(D, F)** Mean ± SEM, *adjusted $P < 0.05$, $^{**}P < 0.01$ by the Student's *t*-test or with Bonferroni method; **(G)** Mean + SEM, $^{**}P < 0.01$ by ANOVA with Tukey's HSD tests for pairwise comparison among three groups.

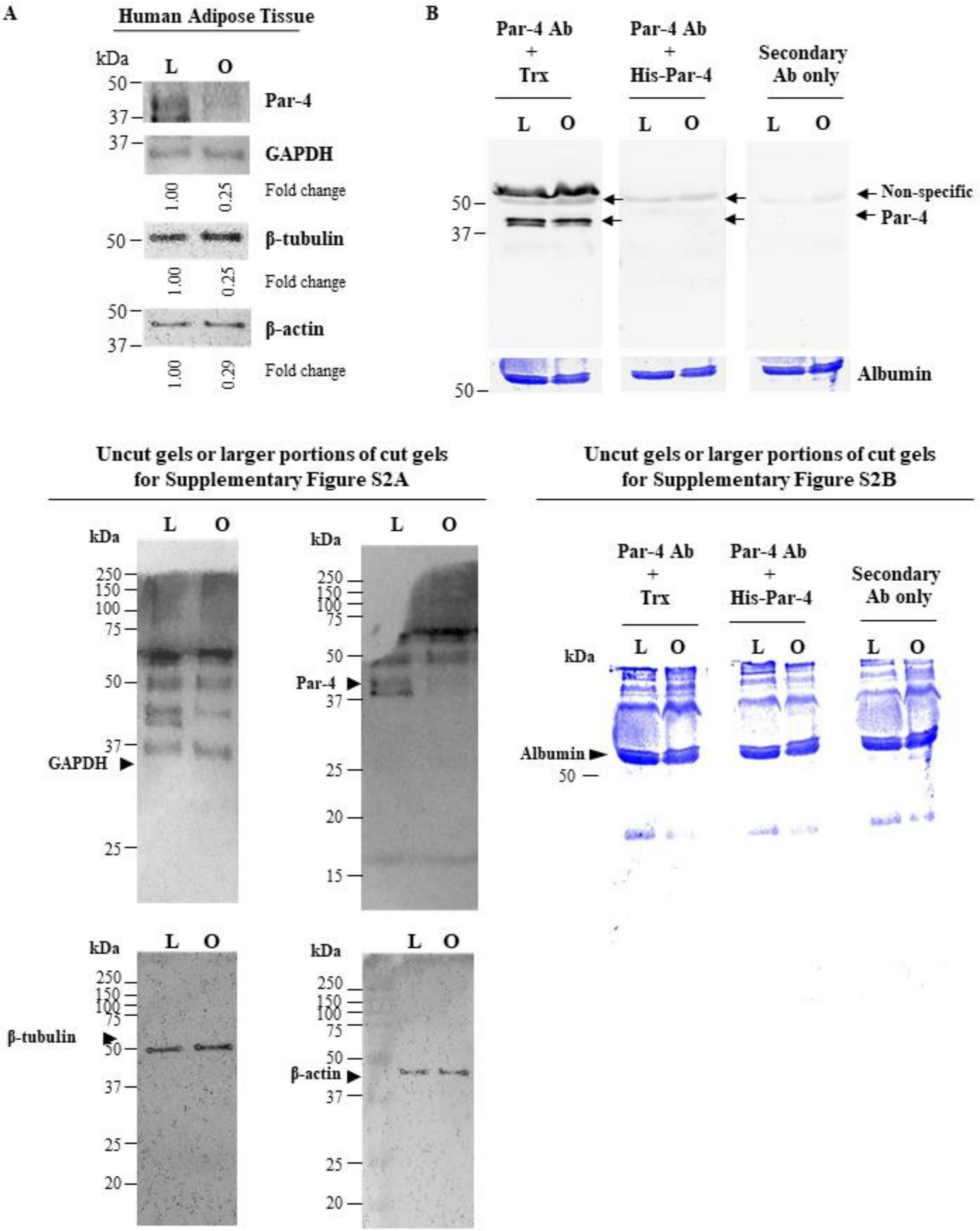


Supplementary Figure S2

**Supplementary Figure S2 (Related to Figure 2). Loading controls and antibody specificity. (A) Western blot analysis of loading controls for adipose tissue.** Visceral adipose tissue protein extracts or plasma from lean and obese age-matched males were subjected to western blot analysis for Par-4. The Par-4 levels in adipose tissue were normalized to either GAPDH, β-tubulin or β-actin levels. As shown, the three loading controls are expressed at comparable levels in the human adipose tissue. **(B) Par-4 antibody is specific for Par-4.** Plasma from lean and obesity individuals with comparable level of circulating Par-4 was subjected to western blot analysis for Par-4. Blots were incubated with either Par-4 antibody preincubated with Trx control protein **(left)**, or Par-4 antibody preincubated with His-Par-4 **(middle)**, or with secondary antibody only without Par-4 primary antibody **(right)**. Preincubation with His-Par-4 protein sequesters the Par-4 antibody, and no bands are observed in the middle blot. Note neutralization of the 40 kDa Par-4 band with purified Par-4 protein indicating specificity of the antibody for the Par-4 protein. The identity of the band above the non-specific band is not known. Circulating concentrations of Par-4 in normal lean individuals are in the 1-5 nM range (data not shown).

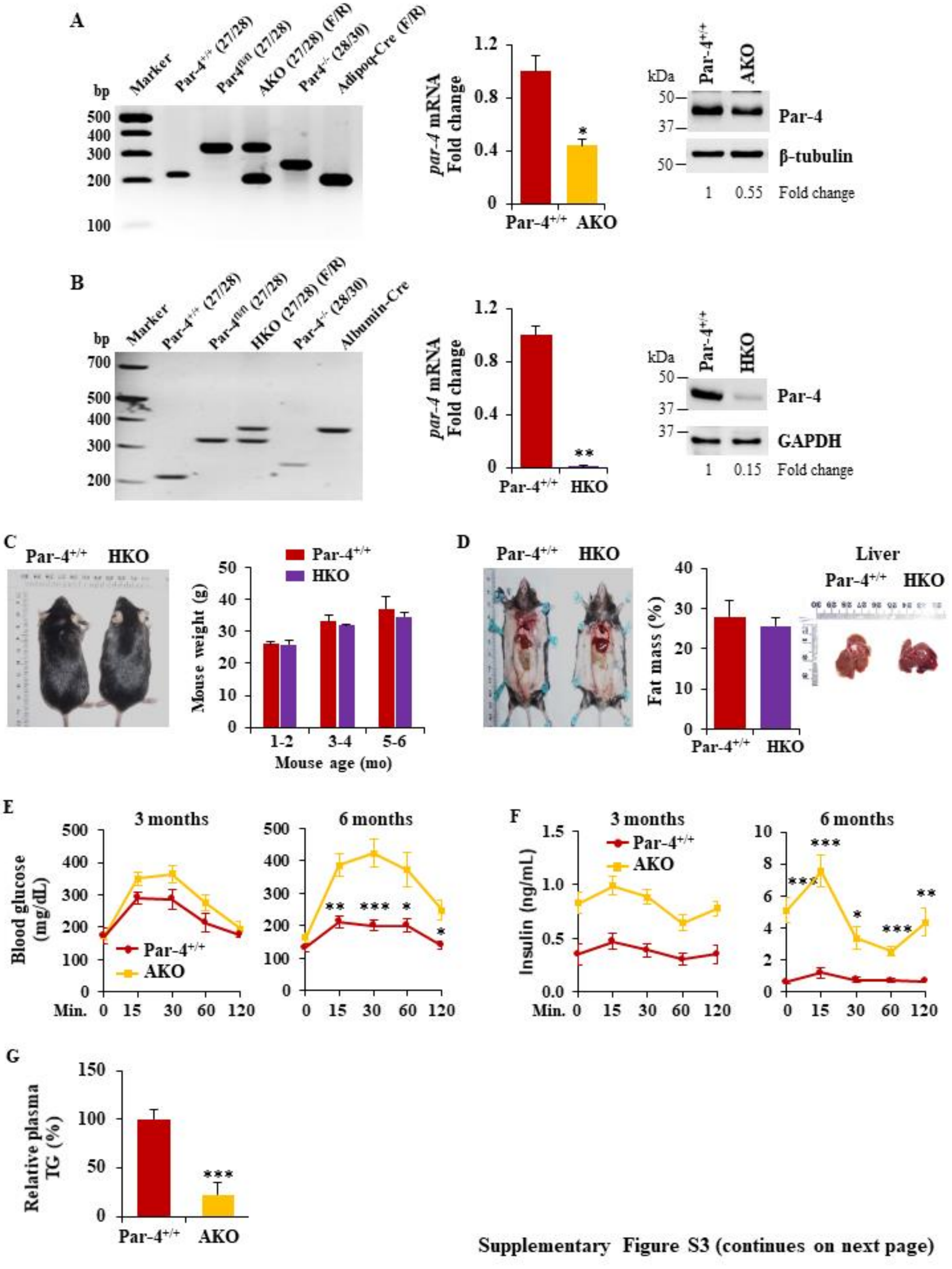


Supplementary Figure S3 (continues on next page)

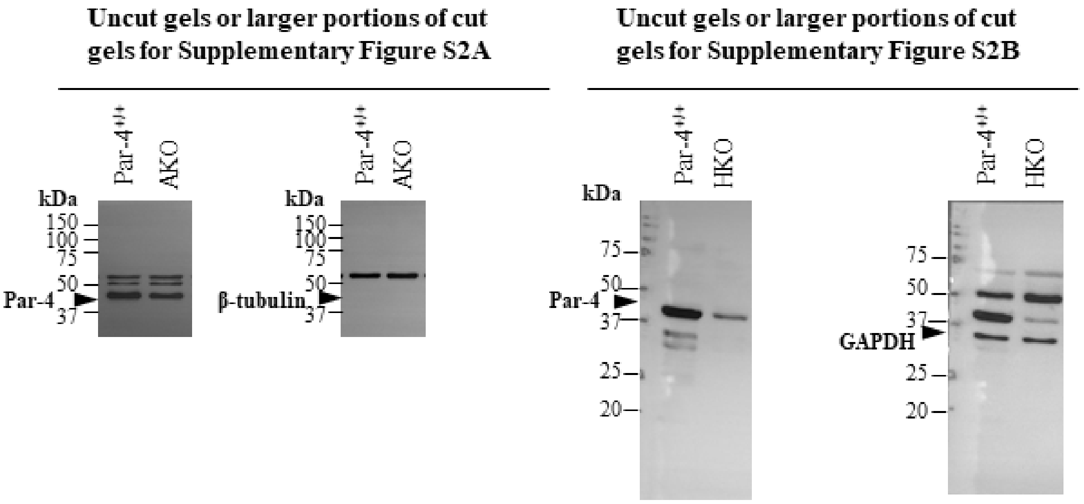


Supplementary Figure S3 (continued)

**Supplementary Figure S3 (Related to Figure 3). Genotype and phenotype of AKO mice and HKO mice. (A) Genotyping of conditional AKO mice**. For conditional knockout (KO) of Par-4 in adipocytes, the fl/fl mice (described in Figure S1) were crossed with Adiponectin-Cre mice (Adipoq-Cre from Jackson Laboratory) to obtain AKO mice. **(Left panel)** DNA from tail clips of Par-$4^{+/+}$, Par-$4^{fl/fl}$, AKO, Par-$4^{-/-}$ and Adiponectin-Cre mice were subjected to PCR using the indicated optimized primers for the Par-4 transgene and the recommended primers for Adiponectin-Cre according to Jackson Laboratory. The resultant DNA fragments were electrophoresed on agarose gels and stained with ethidium bromide. As expected, primer set 27/28 detected a 213 bp fragment in Par-$4^{+/+}$ (wild type) mice and a 326 bp fragment in Par-$4^{fl/fl}$ mice; the Adipoq-Cre forward (F) and reverse (R) primer set detected a 190 bp fragment in Adipoq-Cre mice; primer set 28/30 detected a 248 bp fragment in Par-$4^{-/-}$ mice; and primer set 27/28 and primer set F/R detected a 326 bp fragment and 190 bp fragment, respectively, in AKO mice. **(Middle panel)** RNA from visceral adipose tissue of Par-$4^{+/+}$ and AKO mice was subjected to qPCR analysis for Par-4 and 18S rRNA. Par-4 levels were normalized to 18S rRNA levels. As expected, Par-4 mRNA levels are decreased in visceral white adipose tissue, but not absent, as Par-4 gene is targeted only in adipocytes via the adiponectin promoter, and the stromal vascular fraction in the adipose tissue continues to express Par-4. **(Right panel)** Protein extracts from visceral fat of Par-$4^{+/+}$ and AKO mice were subjected to western blot analysis for Par-4 and GAPDH. Par-4 levels were normalized to GAPDH protein levels. As expected, Par-4 protein levels are decreased in AKO adipose tissue. **(B) Genotyping of conditional HKO mice**. For conditional knockout (KO) of Par-4 in hepatocytes, the fl/fl mice (described in Figure S1) were crossed with Albumin-Cre mice (from Jackson Laboratory) to obtain HKO mice. **(Left panel)** DNA from tail clips of Par-$4^{+/+}$, Par-$4^{fl/fl}$, HKO, Par-$4^{-/-}$ and Albumin-Cre mice were subjected to PCR using the indicated optimized primers for the Par-4 transgene and the recommended primers for Albumin- Cre according to Jackson Laboratory. The resultant DNA fragments were electrophoresed on agarose gels and stained with ethidium bromide. As expected, primer set 27/28 detected a 213 bp fragment in Par-$4^{+/+}$ (wild type) mice and a 326 bp fragment in Par-$4^{fl/fl}$ mice; the Albumin-Cre forward (F) and reverse (R) primer set detected a 350 bp fragment in Albumin-Cre mice; primer set 28/30 detected a 248 bp fragment in Par-$4^{-/-}$ mice; and primer set 27/28 and primer set F/R detected a 326 bp fragment and 350 bp fragment, respectively, in HKO mice. **(Middle panel)** RNA from the liver of Par-$4^{+/+}$ and HKO mice was subjected to qPCR

analysis for Par-4 and 18S rRNA. Par-4 levels were normalized to 18S rRNA levels. As expected, Par-4 mRNA levels were heavily decreased, but not absent, in HKO liver, since the Par-4 gene is targeted only in hepatocytes via the albumin promoter. **(Right panel)** Protein from the liver of Par-$4^{+/+}$ and HKO mice was subjected to western analysis for Par-4 and GAPDH. Par-4 levels were normalized to GAPDH levels. As expected, Par-4 protein levels are decreased in HKO liver. **(C) HKO mice are not obese.** Body weights of age-matched male mice (1-6 months, n=3-10) **(right panel)** and representative images of 9-month-old male mice **(left panel)** on standard chow are shown. Female HKO mice were not obese (n= 3-16; data not shown). **(D) HKO mice show similar fat mass and liver size as Par-$4^{+/+}$ mice.** Echo-MRI analysis was performed on 6-8-month-old HKO (n=7) and Par-$4^{+/+}$ (n=5) mice **(middle panel)**, and representative 9-month-old mouse images **(left panel)** and liver images **(right panel)** are shown. Older mice are shown to indicate that there is no delayed phenotype. **(E) Glucose levels in AKO and Par-$4^{+/+}$ mice.** Glucose was orally administered to 3-month-old (n=4) and 8-month-old (n=11) mice maintained on standard chow, and plasma levels of glucose were determined. **(F) Insulin levels in AKO and Par-$4^{+/+}$ mice.** Glucose was orally administered to 3-month-old (n=4) and 8-month-old (n=8) mice maintained on standard chow and plasma levels of insulin were determined. **(G) Plasma triglycerides are decreased in AKO mice**. Steady-state (no fasting) plasma was collected from Par-$4^{+/+}$ (n=9) and Par-$4^{-/-}$ (n=8) 3-month-old female mice. Plasma triglyceride levels were determined, and percentage decrease is shown. **(D, F, G)** Mean ± SEM, *adjusted $P < 0.05$, **$P < 0.01$, ***$P < 0.001$ by the Student's *t*-test or with Bonferroni method.

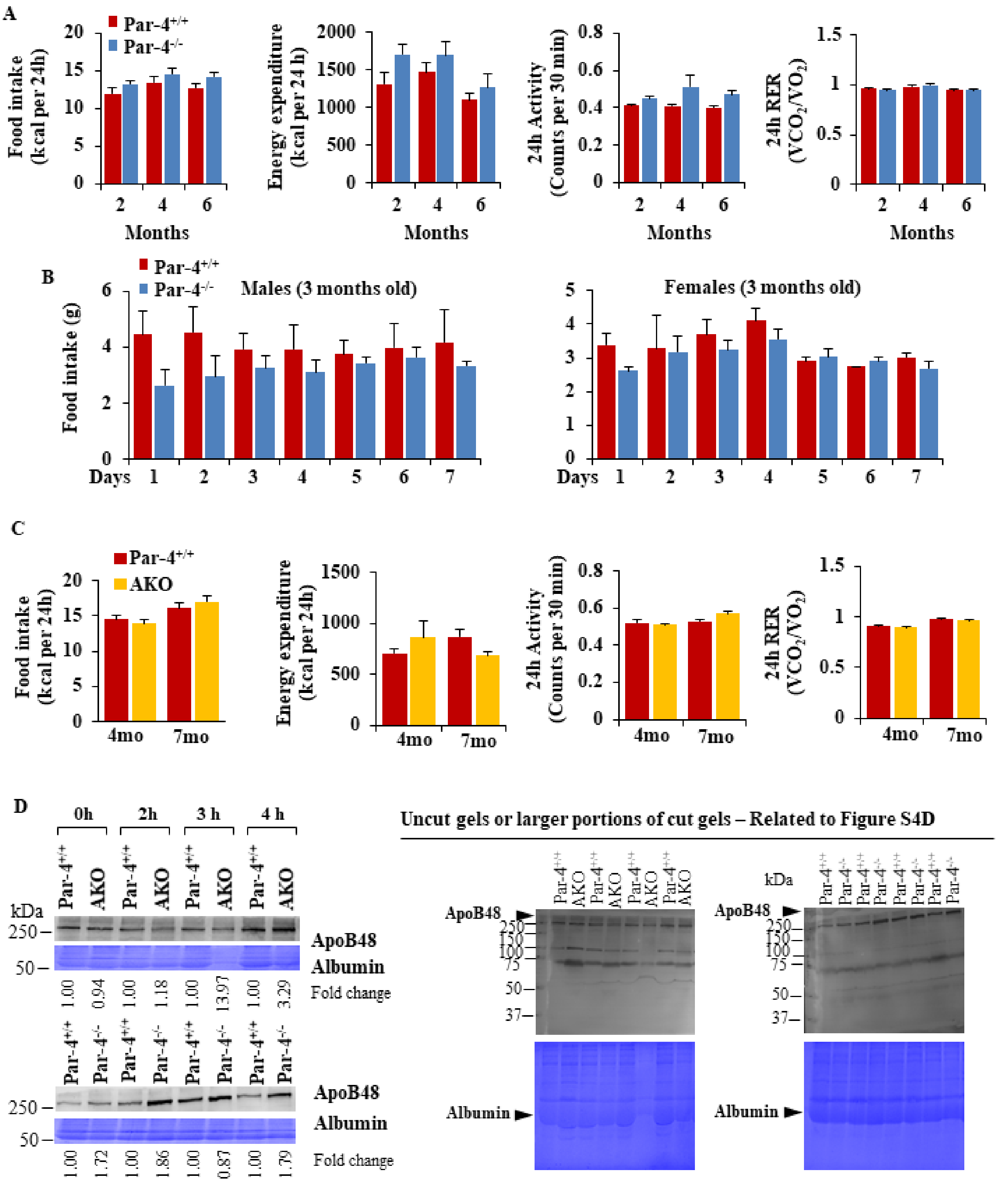


Supplementary Figure S4

**Supplementary Figure S4 (Related to Figure 4). Behavioral and metabolic features of Par-4$^{-/-}$, AKO and Par-4$^{+/+}$ mice on standard chow diet. (A, C) Behavioral features of Par-4$^{-/-}$ and Par-4$^{+/+}$ mice (A), and AKO and Par-4$^{+/+}$ mice (C).** Caloric intake, energy expenditure, physical activity, and respiratory exchange rate of Par-4$^{-/-}$, AKO, and Par-4$^{+/+}$ mice in the indicated age groups were determined in the Center of Biomedical Research Excellence in Obesity and Cardiovascular Diseases metabolic phenotypic core. Results were normalized to corresponding lean mass determined by Echo-MRI analysis. **(B) Feeding is similar in both male and female Par-4$^{-/-}$ mice vs Par-4$^{+/+}$ mice in paired-feeding experiment.** Equal amount of food was provided to both Par-4$^{-/-}$ and Par-4$^{+/+}$ mice and food consumption was measured by weighting the food pellets every day for the course of seven days. **(D) Apolipoprotein B48 secretion is increased in both AKO and Par-4$^{-/-}$ mice.** Plasma from AKO, Par-4$^{-/-}$ and Par-4$^{+/+}$ mice was subjected to TG analysis and examined by western blot analysis for ApoB48. ApoB48 levels were normalized to albumin levels and fold change at each time point is shown.

**(A-C)** Mean ± SEM are shown. $P > 0.05$, by Student's *t*-test. There were no significant differences between either Par-4$^{-/-}$ and Par-4$^{+/+}$ or AKO and Par-4$^{+/+}$ mice in endpoints with adjustment of final lean mass. The P values were calculated based on the linear regression models with mouse group and final lean mass as covariates. There were no differences in diurnal variation across these measures.

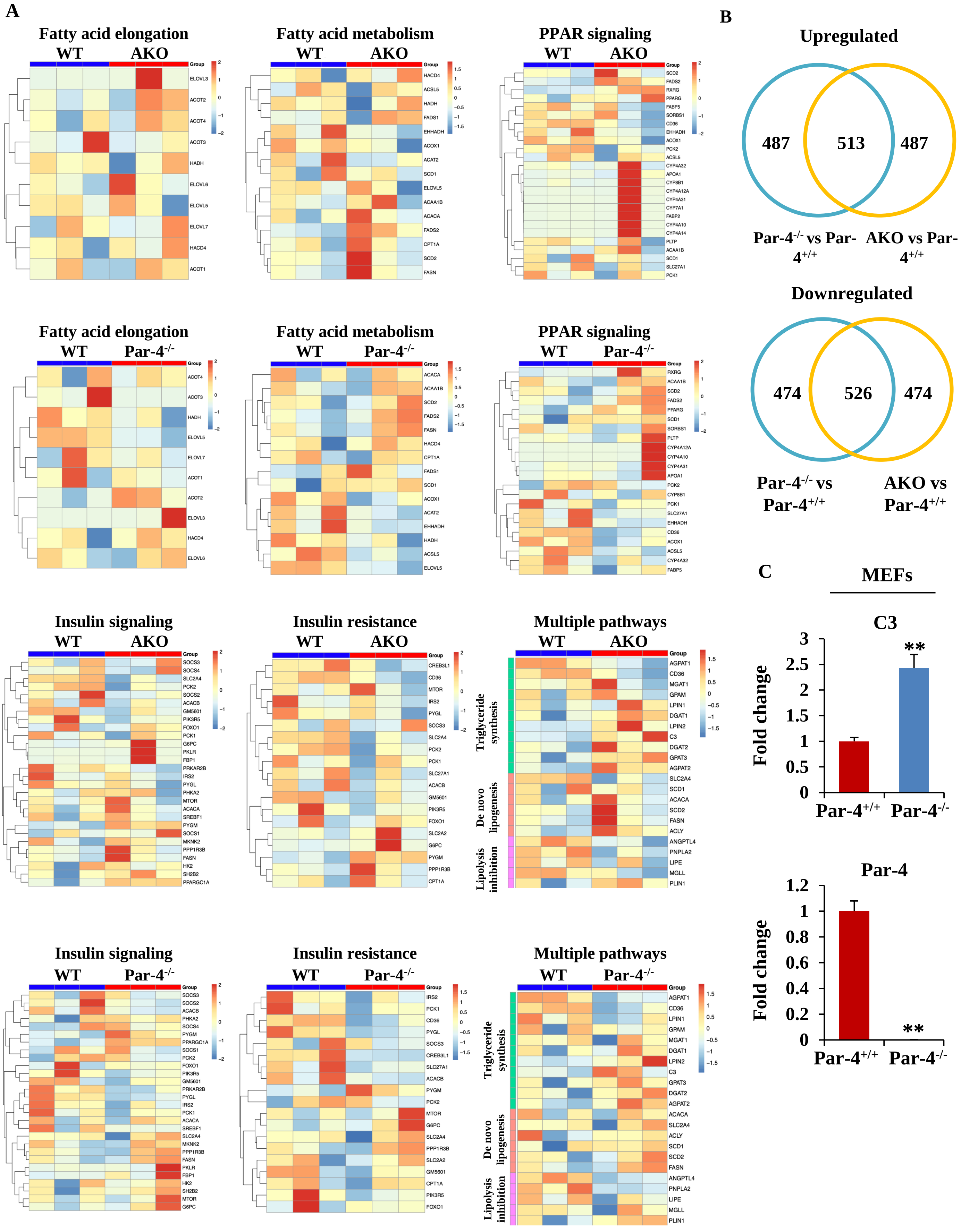


**Supplementary Figure S5 (Enlarged images of S5A presented on the next 12 pages)**

**Supplementary Figure S5A**

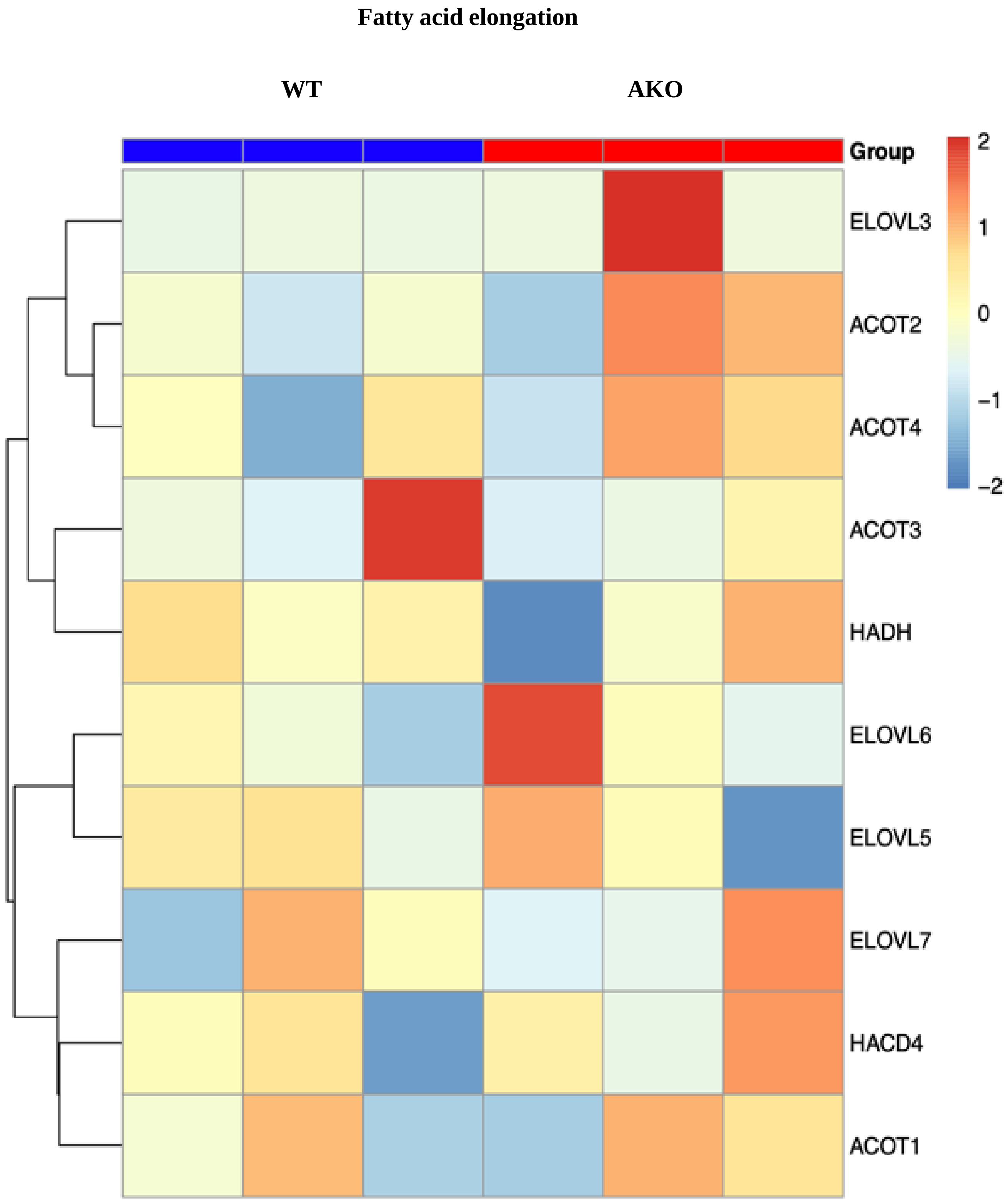


**Supplementary Figure S5A (continues on next page)**

**Supplementary Figure S5A**

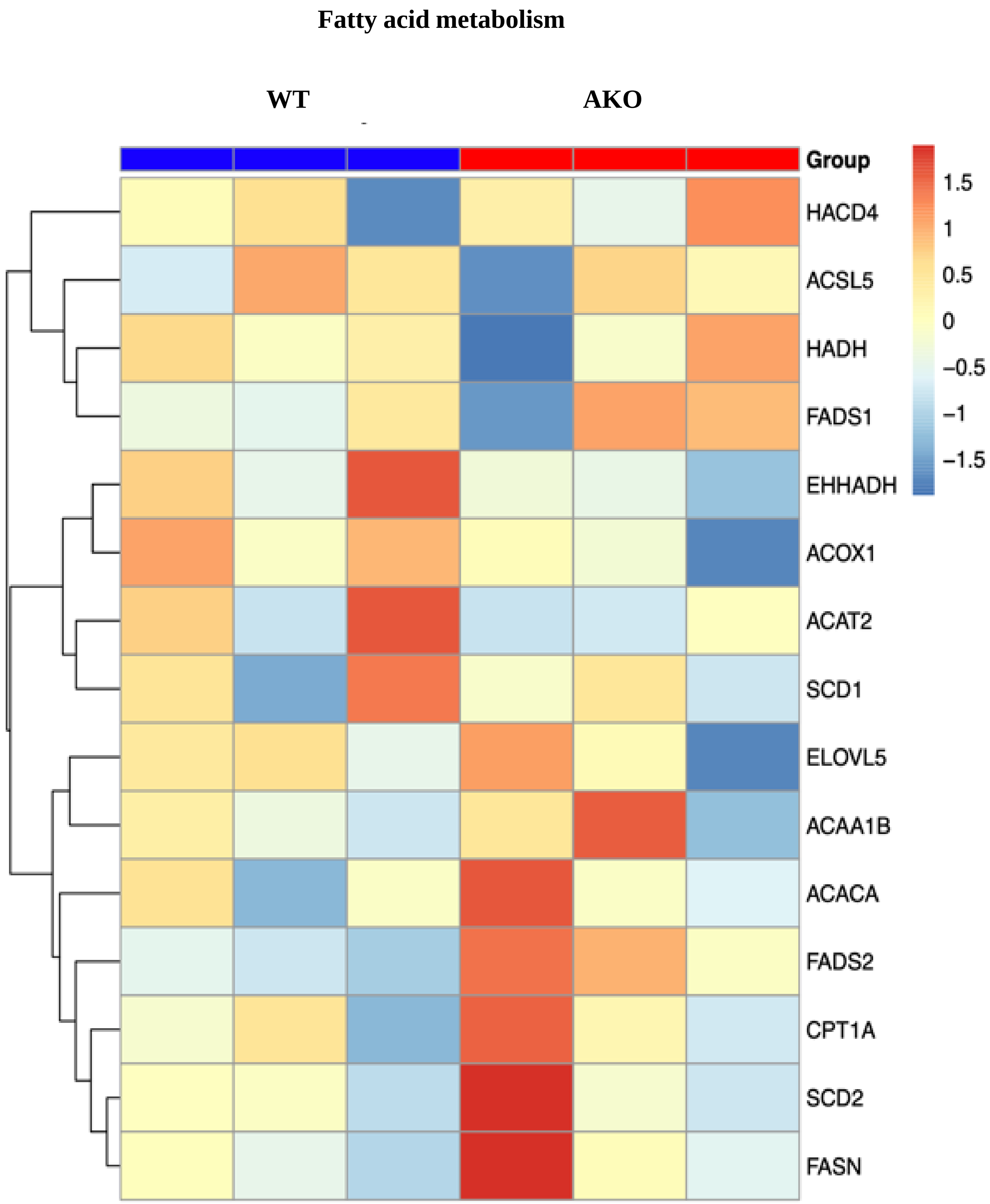


**Supplementary Figure S5A (continues on next page)**

**Supplementary Figure S5A**

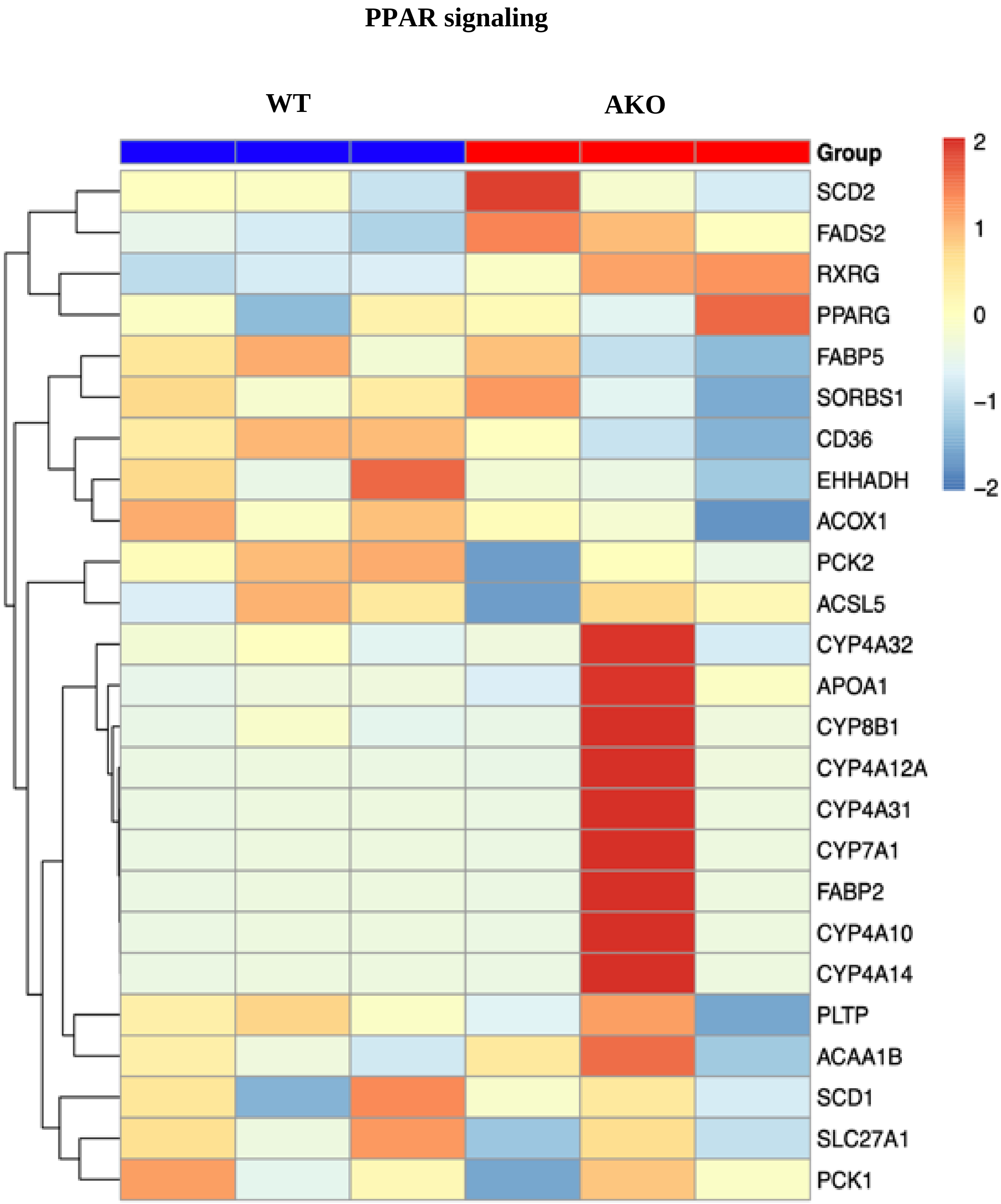


**Supplementary Figure S5A** **(continues on next page)**

**Supplementary Figure S5A**

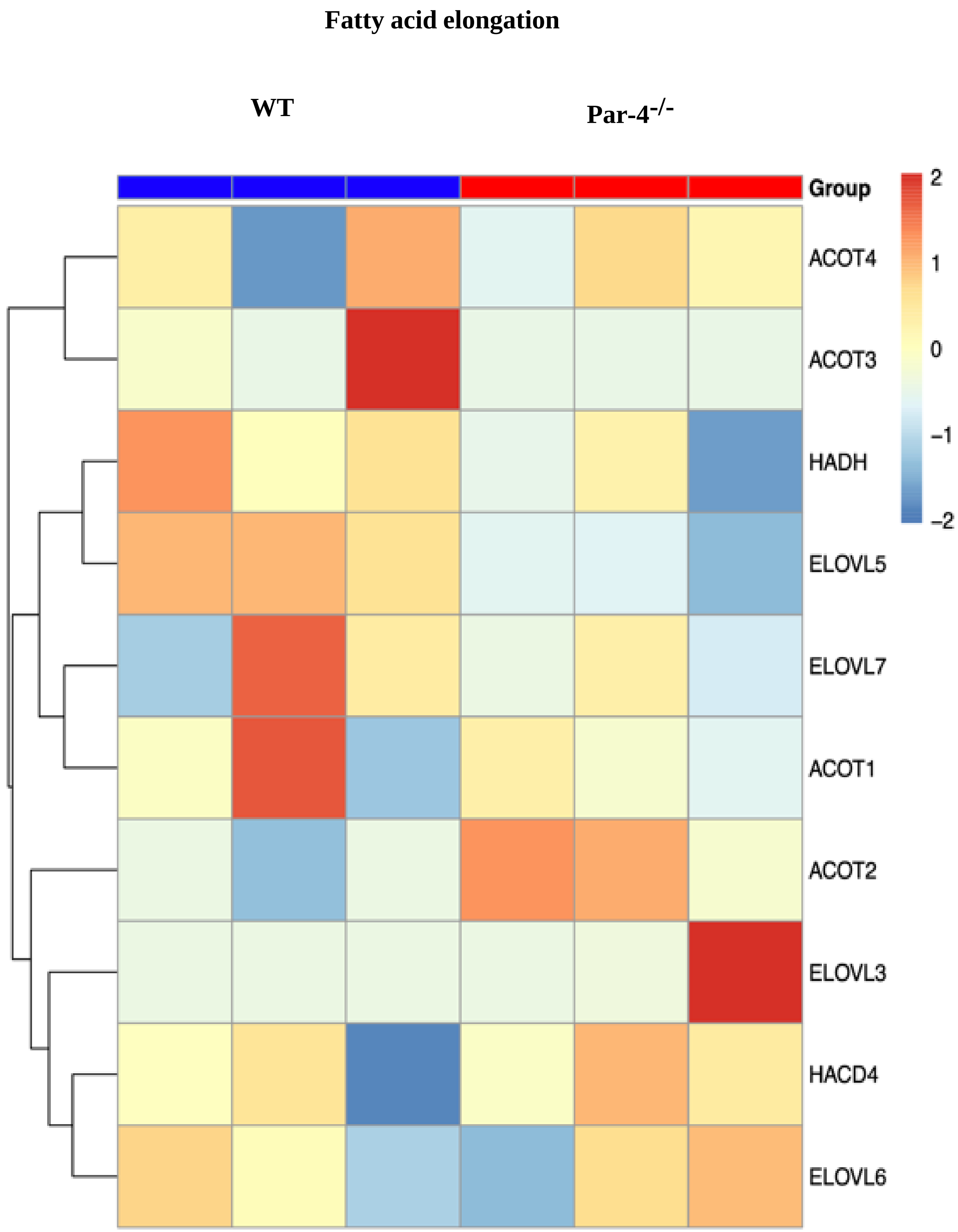


**Supplementary Figure S5A (continues on next page)**

**Supplementary Figure S5A**

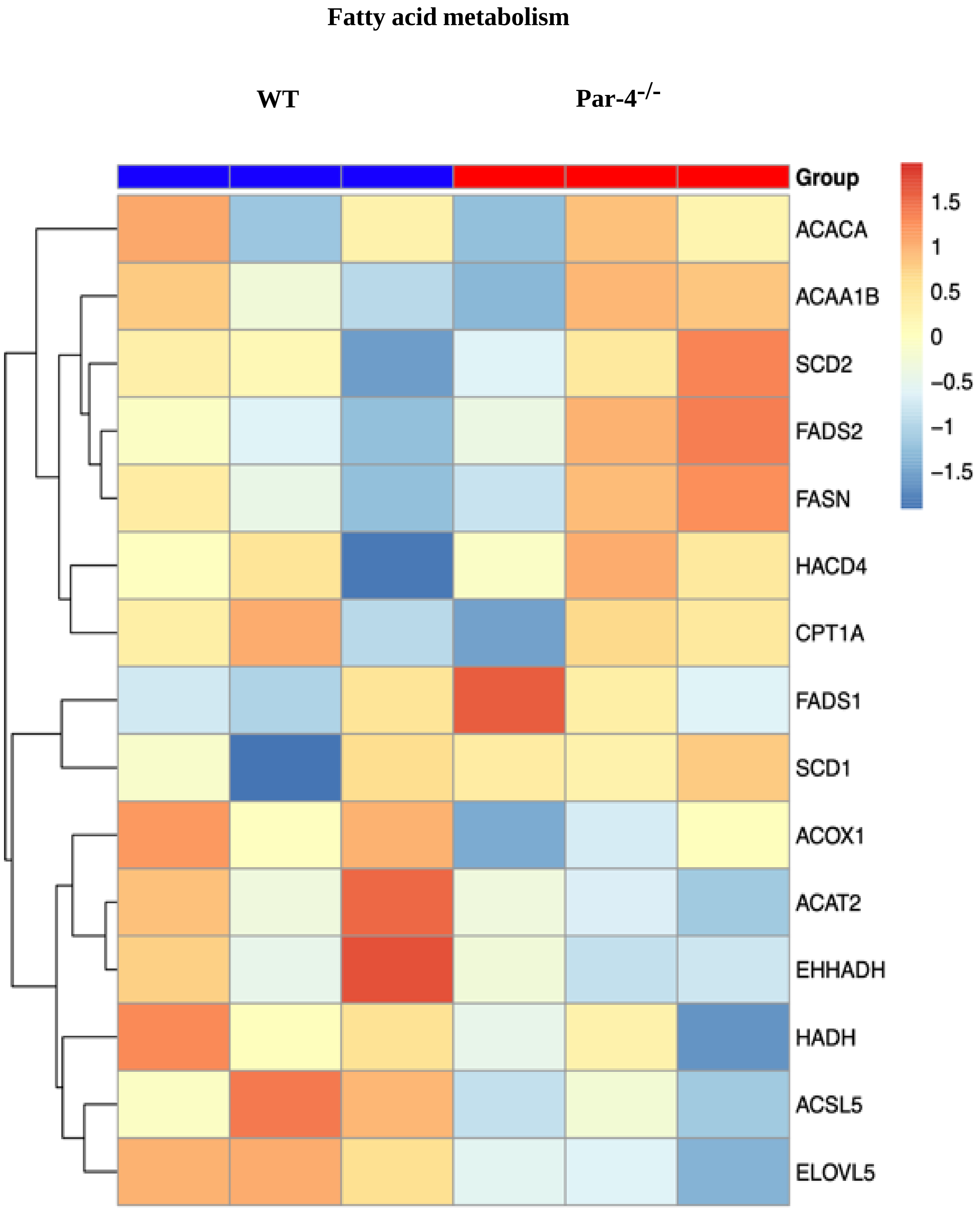


Supplementary Figure S5A (continues on next page)

**Supplementary Figure S5A**

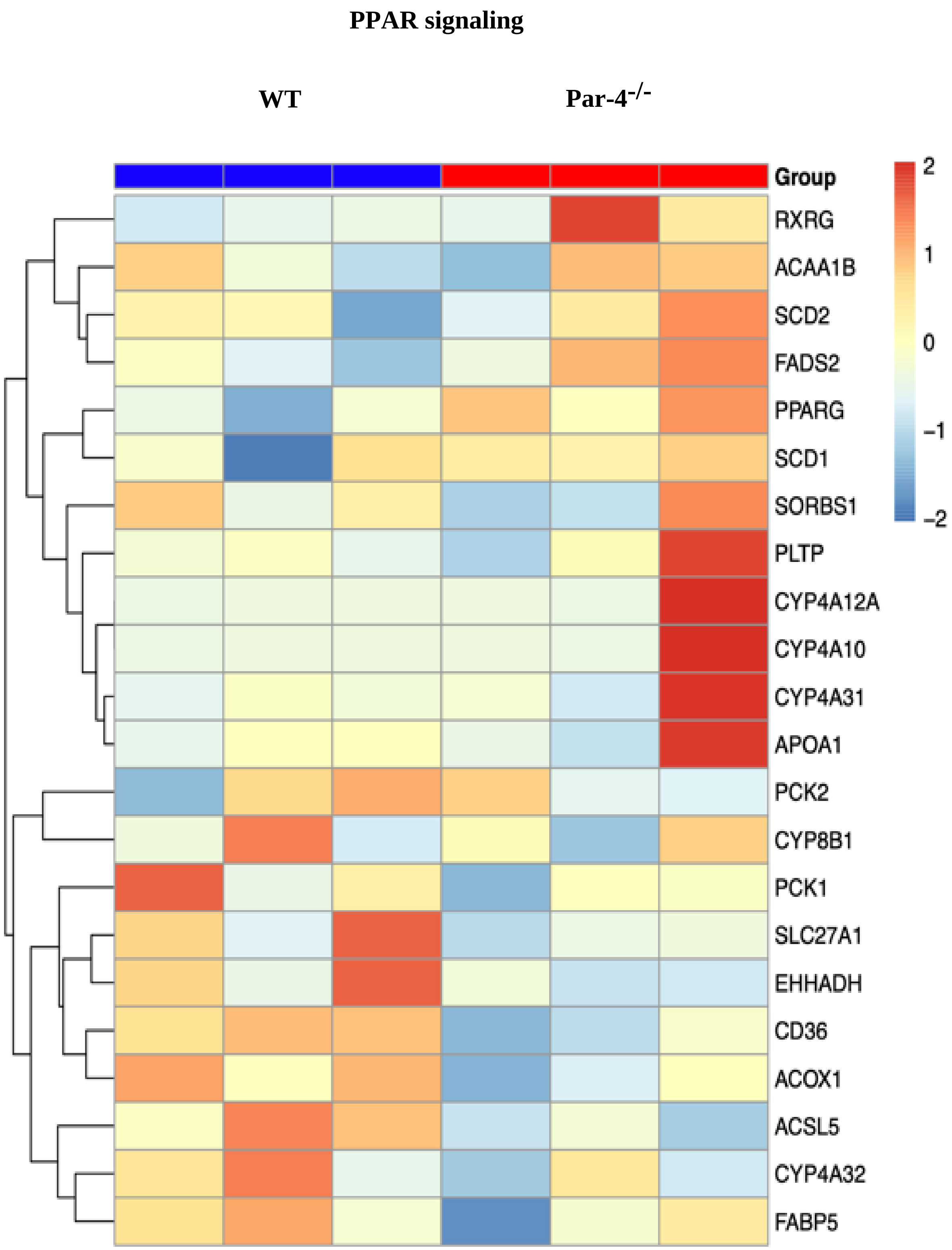


**Supplementary Figure S5A (continues on next page)**

**Supplementary Figure S5A**

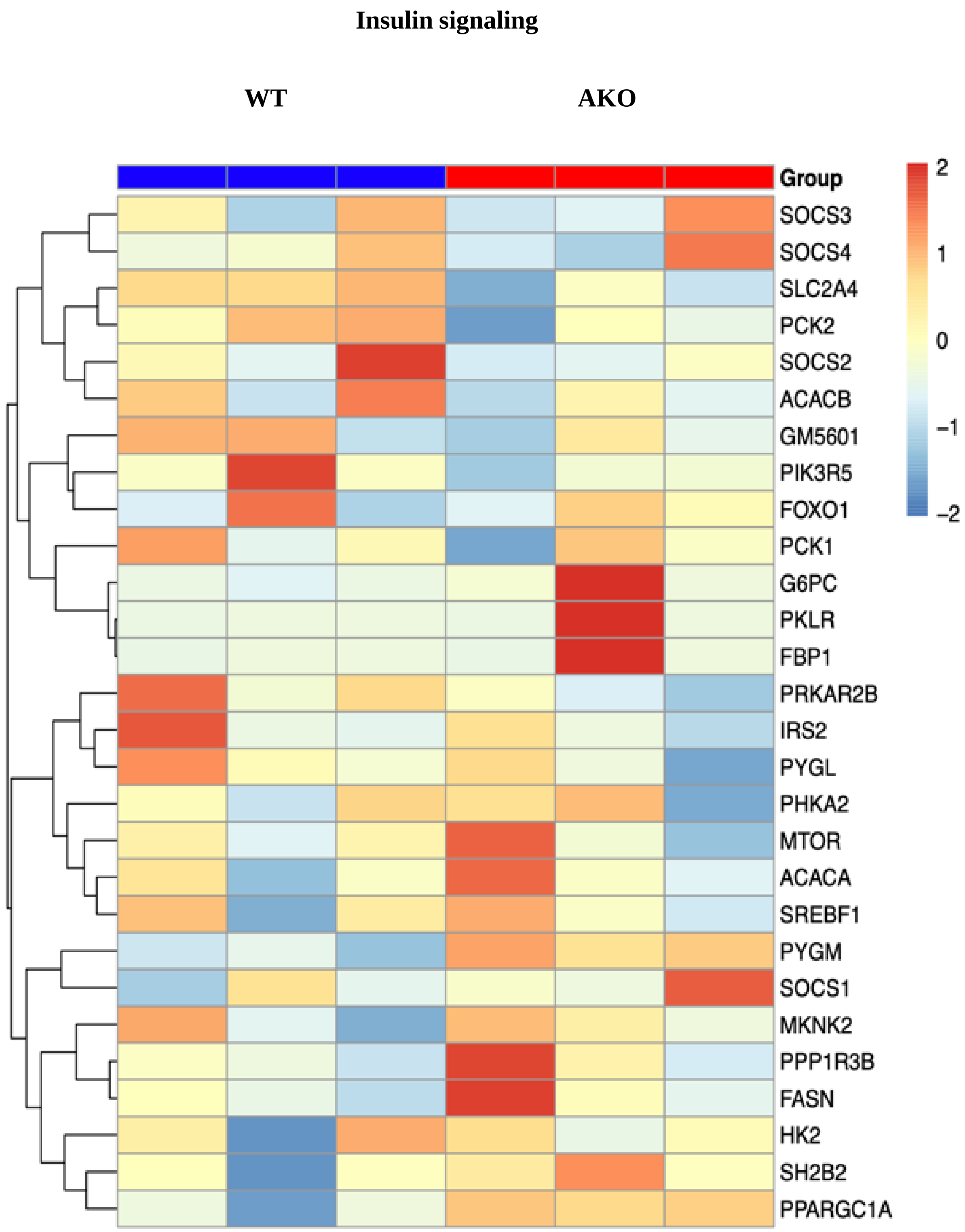


**Supplementary Figure S5A (continues on next page)**

Supplementary Figure S5A

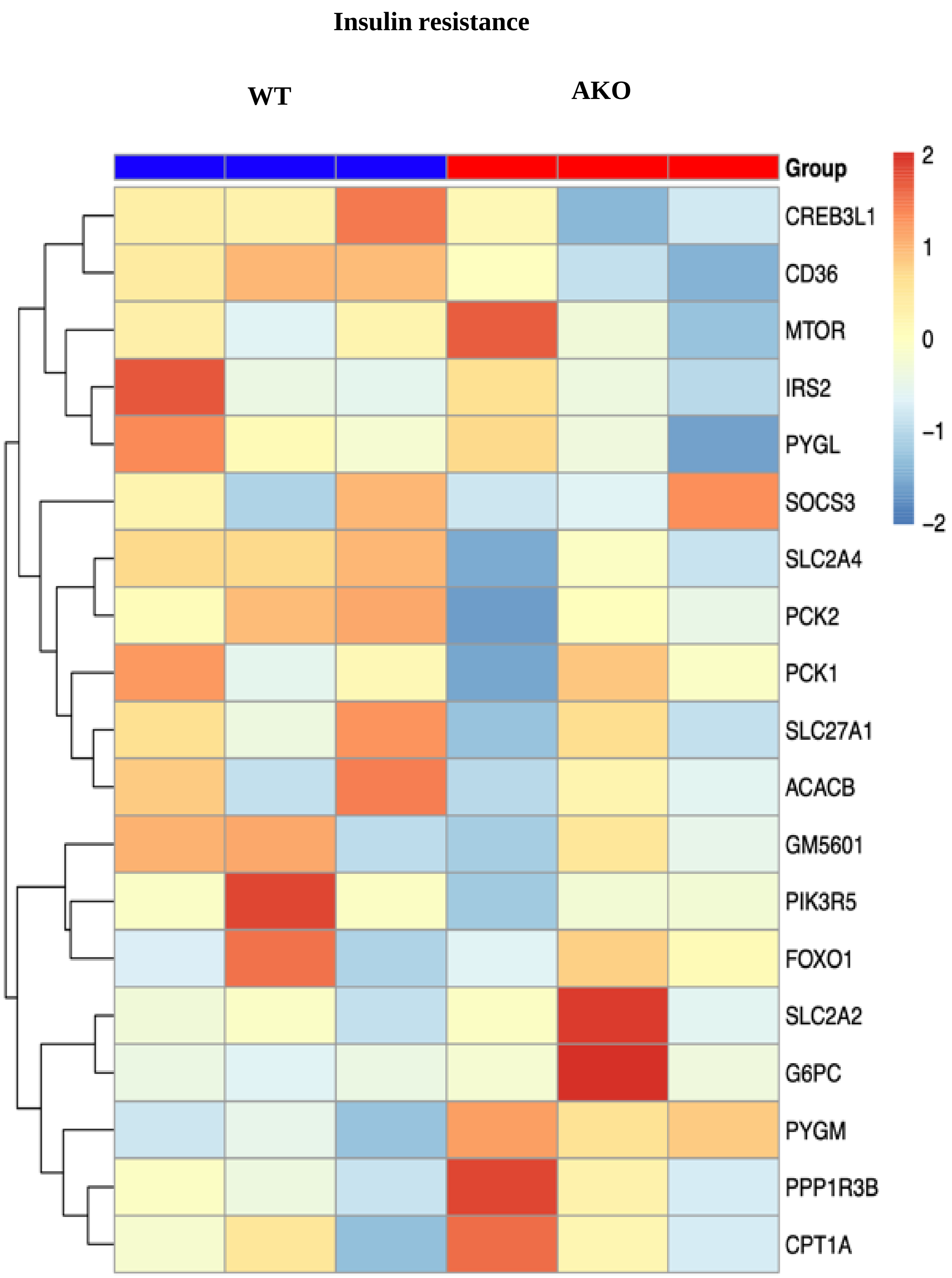


Supplementary Figure S5A (continues on next page)

Supplementary Figure S5A

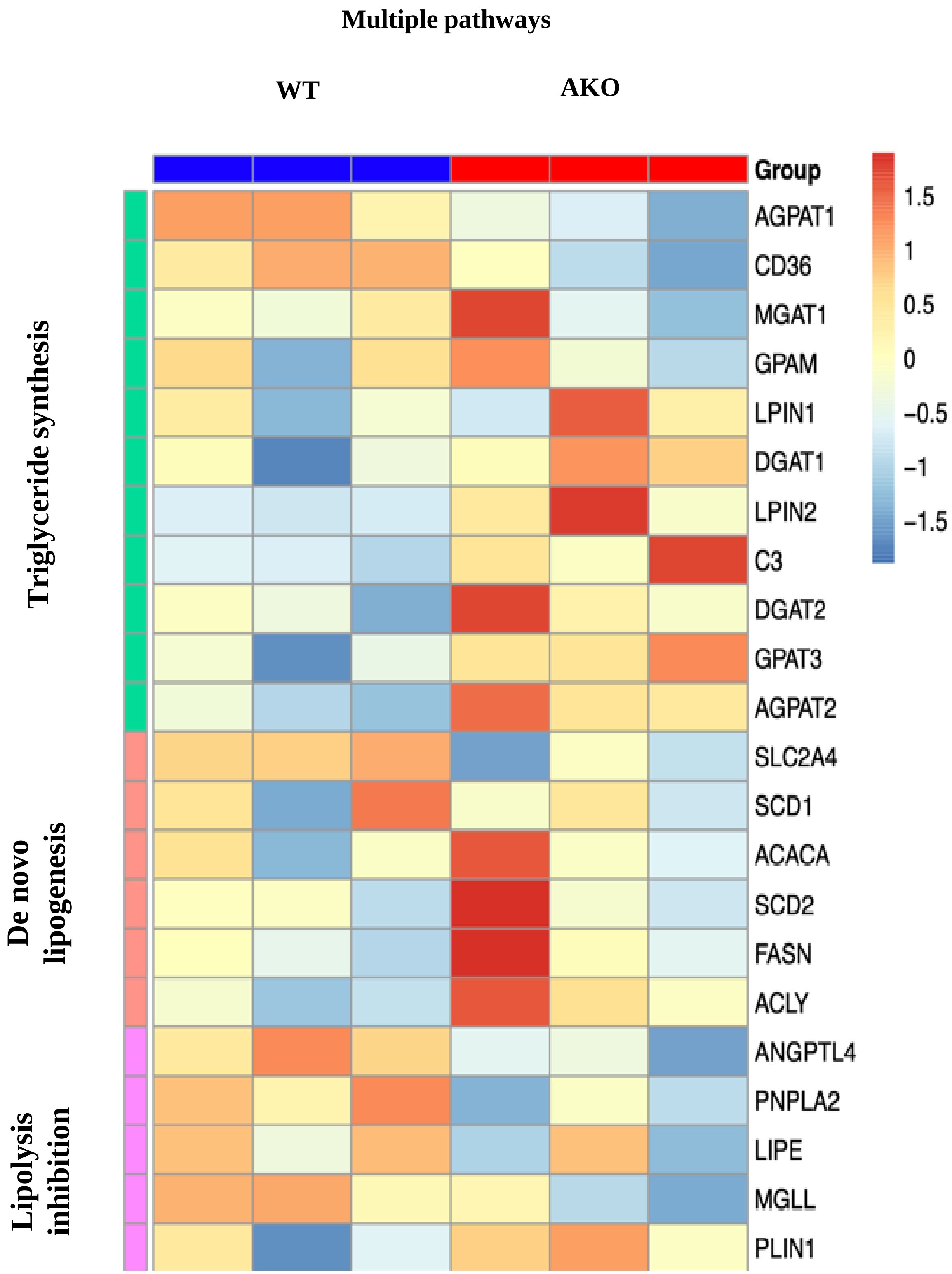


Supplementary Figure S5A (continues on next page)

**Supplementary Figure S5A**

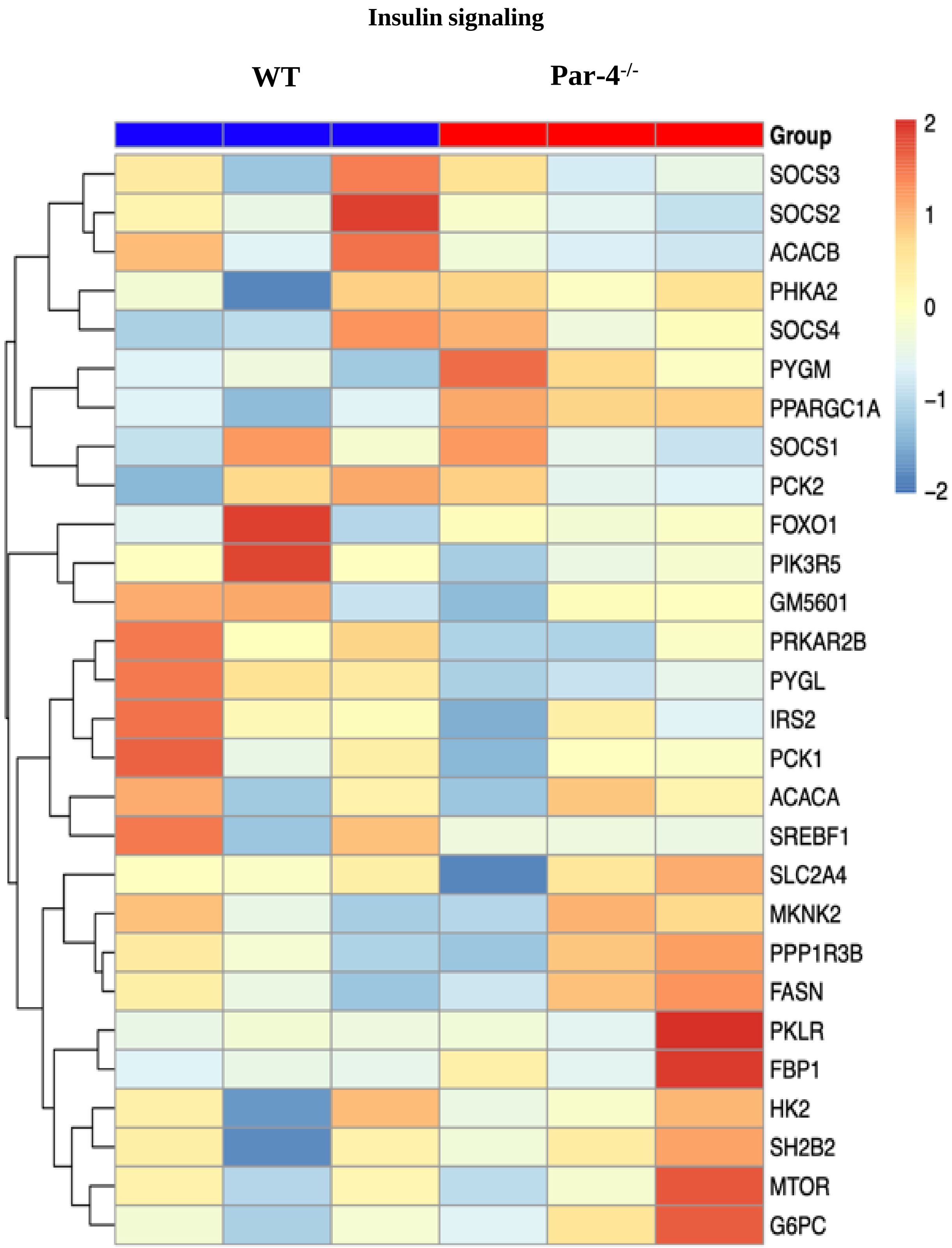


**Supplementary Figure S5A** (continues on next page)

**Supplementary Figure S5A**

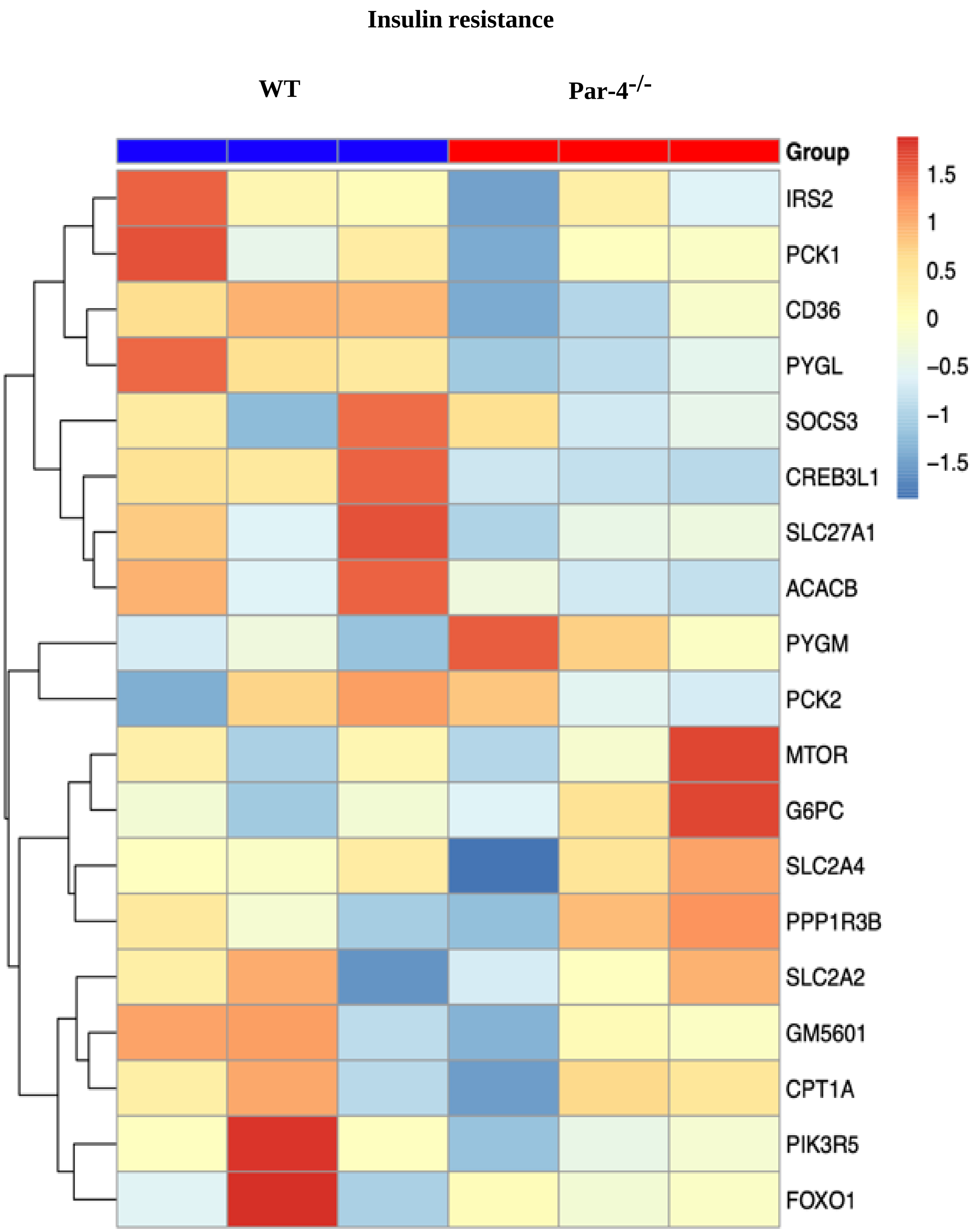


**Supplementary Figure S5A (continues on next page)**

**Supplementary Figure S5A**

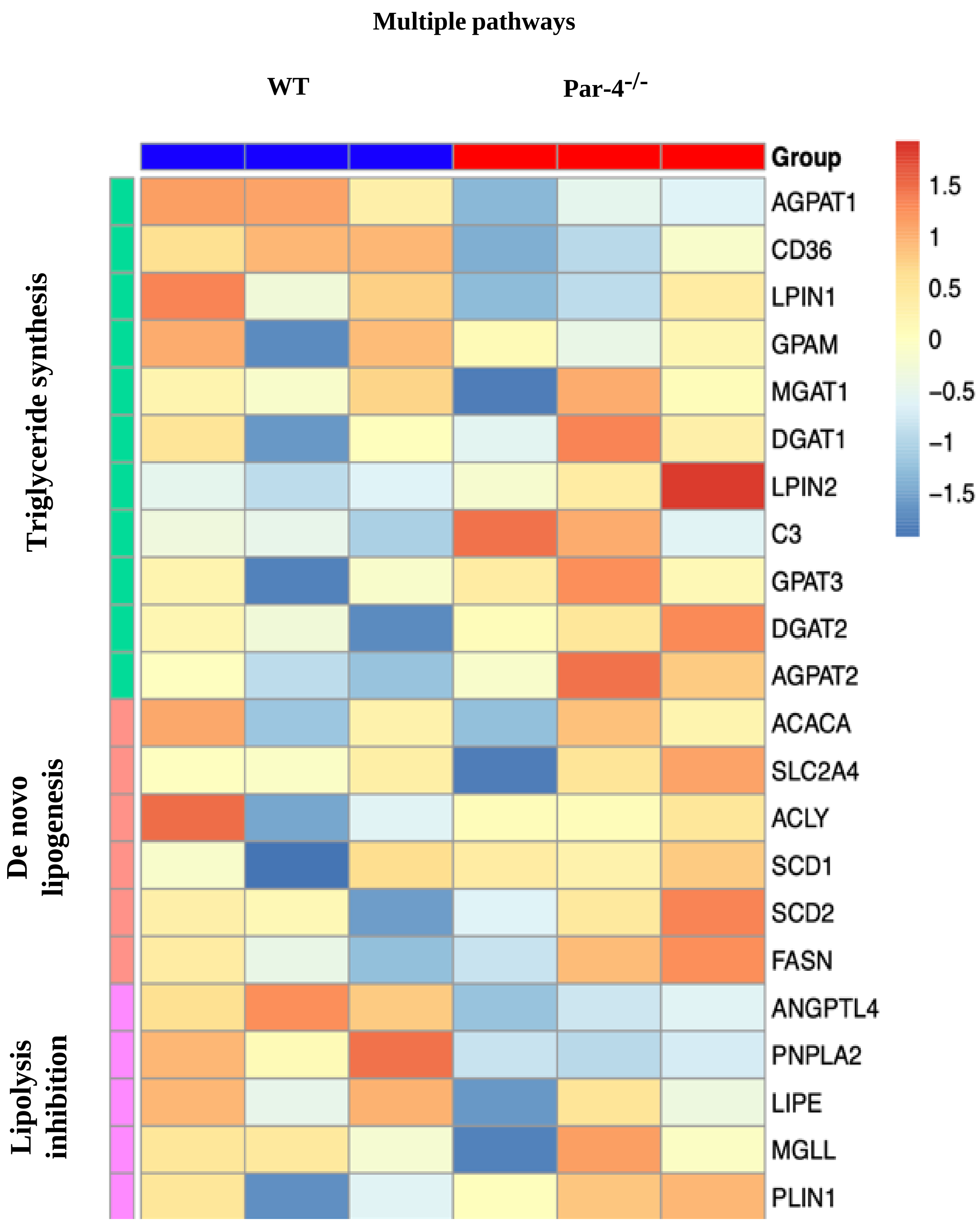

**Supplementary Figure S5 (Related to Figure 5). RNA-Seq analysis of Par-4$^{+/+}$, AKO and Par-4$^{-/-}$ mice and involvement of ASP in obesity phenotype. (A) Multiple pathways analysis.** We performed RNA-Seq on visceral adipose tissue samples from 11-week old AKO, Par-4$^{-/-}$ and Par-4$^{+/+}$ male mice (n=3 per group). The heatmaps of the selected pathways under various group comparisons were generated by the R package "pheatmap" based on the normalized and log2-transformed counts data. The normalization of counts data was performed based on the default normalization algorithm of the R package "DESeq2". In the case of a single pathway presented on a heatmap, the involved genes were clustered based on the Euclidean Cluster Extraction algorithm. When multiple pathways were presented on the same heatmap, the genes involved within each pathway were sorted on the heatmap in ascending order of fold change. Our analysis focused on calculating the average gene expression level for each group. We defined differentially expressed genes as those with log2 fold change >0.5. Given the large-scale nature of the RNA-Seq study, our analysis identified a set of candidate genes for further validation. For example, the c3 gene identified in RNA-Seq was validated by RT-PCR on multiple cohorts of mice. **(B) Genes differentially expressed in Par-4$^{-/-}$ and AKO relative to Par-4$^{+/+}$ mouse adipose tissues.** Venn diagram showing number of overlapping and distinct genes that were up- or down-regulated in Par-4$^{-/-}$ and AKO mice relative to Par-4$^{+/+}$ mice (n=3). Results imply that more than 50% of genes were common in the adipose tissue of Par-4$^{-/-}$ and AKO mice. **(C) C3 is upregulated in Par-4$^{-/-}$ MEFs.** RNA samples from Par- 4$^{+/+}$ and Par-4$^{-/-}$ MEFs were subjected to qPCR analysis for C3 **(top panel)**, Par-4 **(bottom panel)** and 18S rRNA. C3 and Par-4 levels were normalized to 18S rRNA and fold change was calculated. Mean ± SEM, $^{**}P < 0.01$ by the Student's *t*-test. Except for p21/*WAF1*, the other well-known p53 regulated genes (such as *PIG3*, *PUMA*, *NOXA*, *BAX*, *TIGAR*) were not altered in Par-4$^{-/-}$ or AKO tissues as judged by RNA-Seq or Western blot analysis of adipose tissue.

**References:**

1. R package "pheatmap": Raivo Kolde (2019). pheatmap: Pretty Heatmaps. R package version 1.0.12. https://CRAN.R-project.org/package=pheatmap

2. R package "DESeq2": Love MI, Huber W, Anders S (2014). Moderated estimation of fold change and dispersion for RNA-seq data with DESeq2. Genome Biology, 15, 550. doi: 10.1186/s13059-014-0550-8.

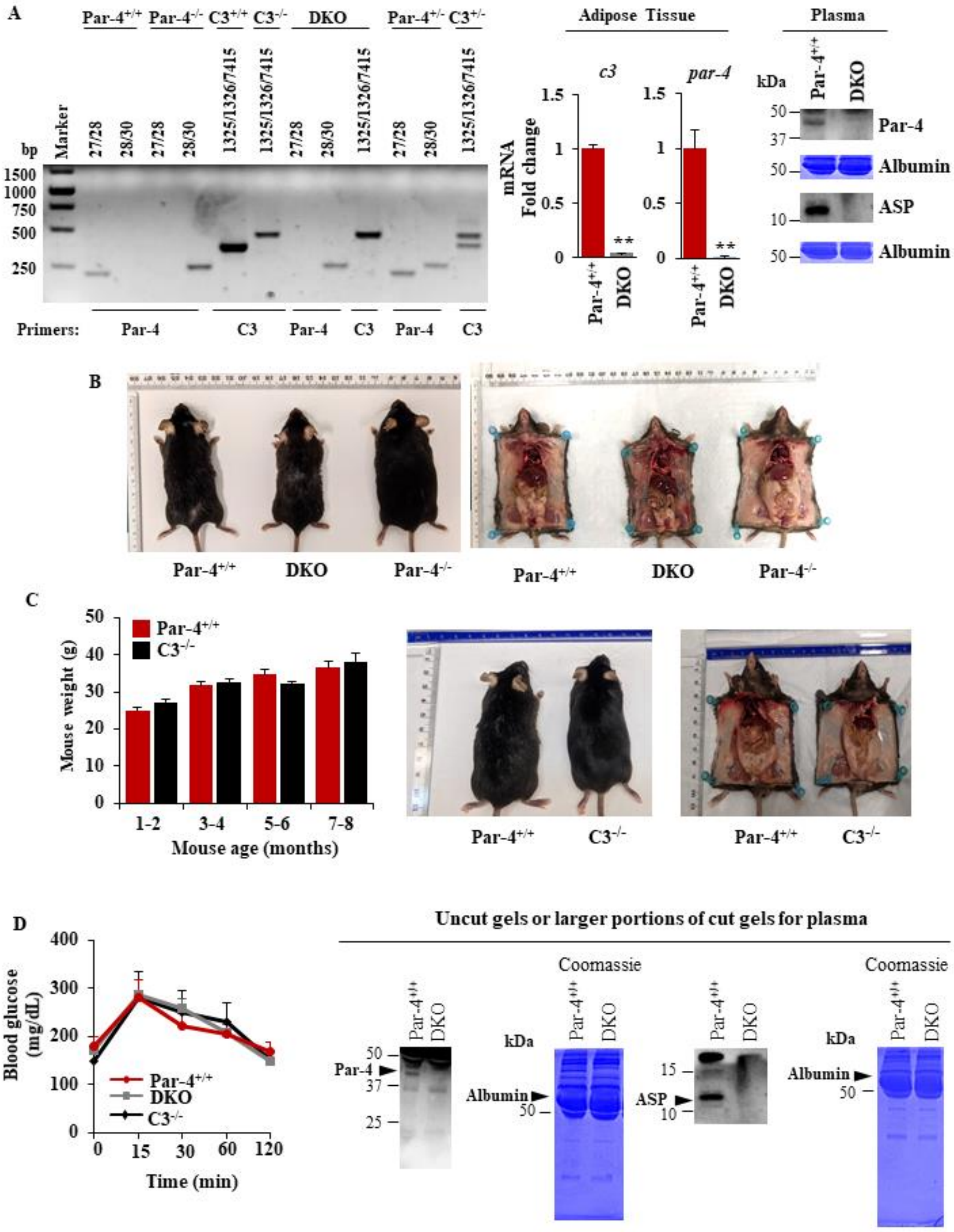


Supplementary Figure S6

**Supplementary Figure S6 (Related to Figure 6). Genotyping and phenotyping of Par-4/C3 double-knockout (DKO) mice. (A)** For whole-body double-knockout of Par-4 and C3 (DKO), the Par-$4^{+/-}$ (described in Figure S1) were crossed with $C3^{-/-}$ mice (from Jackson Laboratory) to obtain Par-4/C3 DKO mice. **(Left panel)** DNA from tail clips of Par-$4^{+/+,}$ Par-$4^{-/-}$, C3 $^{+/+}$, $C3^{-/-}$, Par-$4^{-/-}$/$C3^{-/-}$ (DKO), Par-$4^{+/-}$ and $C3^{+/-}$ mice were subjected to PCR using the indicated optimized primers for the Par-4 transgene and the recommended primers for C3 according to Jackson Laboratory. The resultant DNA fragments were electrophoresed on agarose gels and stained with ethidium bromide. As expected, primer set 27/28 detected a 213 bp fragment in Par-$4^{+/+}$ (wild type); primer set 28/30 detected a 248 bp fragment in Par-$4^{-/-}$ mice; and primer set 1325, 1326, 7415 detected a 350 bp fragment in $C3^{+/+}$ and a 500 bp $C3^{-/-}$. DKO mice show the 248bp fragment with 28/30 primer set and 500bp fragment with 1325, 1326, 7415 primer set. Par-$4^{+/-}$ mice show fragments with both 27/28 and 28/30 primer sets and $C3^{+/-}$ mice show two fragments with 1325, 1326, 7415 set. **(Middle panel)** RNA from visceral adipose tissue of Par-$4^{+/+}$ and DKO mice was subjected to qPCR analysis for C3, Par-4 and 18S rRNA. C3 and Par-4 levels were normalized to 18S rRNA levels. As expected, C3 and Par-4 RNA levels are absent in visceral white adipose tissue of DKO mice. **(Right panel)** Plasma from Par-$4^{+/+}$ and DKO mice was subjected to western blot analysis for Par-4 and ASP. Albumin levels were determined in parallel Coomassie Blue gels. **(B) Fat accumulation in DKO mice.** Overall body size and fat accumulation are similar in control and DKO mice. Par-$4^{-/-}$ mouse is heavier and shows increased fat accumulation when compared to either control or DKO mice (photo depicts 5-6-month-old mice). **(C) $C3^{-/-}$ mice are not obese.** Weight of age- and gender-matched $C3^{-/-}$ (n = 5, 8, 5 and 4 for each time point, respectively) and Par-$4^{+/+}$ (n = 7, 8, 5 and 4 for each time point, respectively) mice was determined over the course of 7 months. Representative images of 8-month-old male mice are shown **(middle and right panels). (D) Glucose levels in DKO, $C3^{-/-}$ and Par-$4^{+/+}$ mice.** Glucose was orally administered to 6-month DKO (n=4), $C3^{-/-}$ (n=4) and Par-$4^{+/+}$ (n=6) mice maintained on standard chow, and plasma levels of glucose were determined. **(a)** Mean ± SEM, $^{**}P < 0.01$ by the Student's *t*-test.

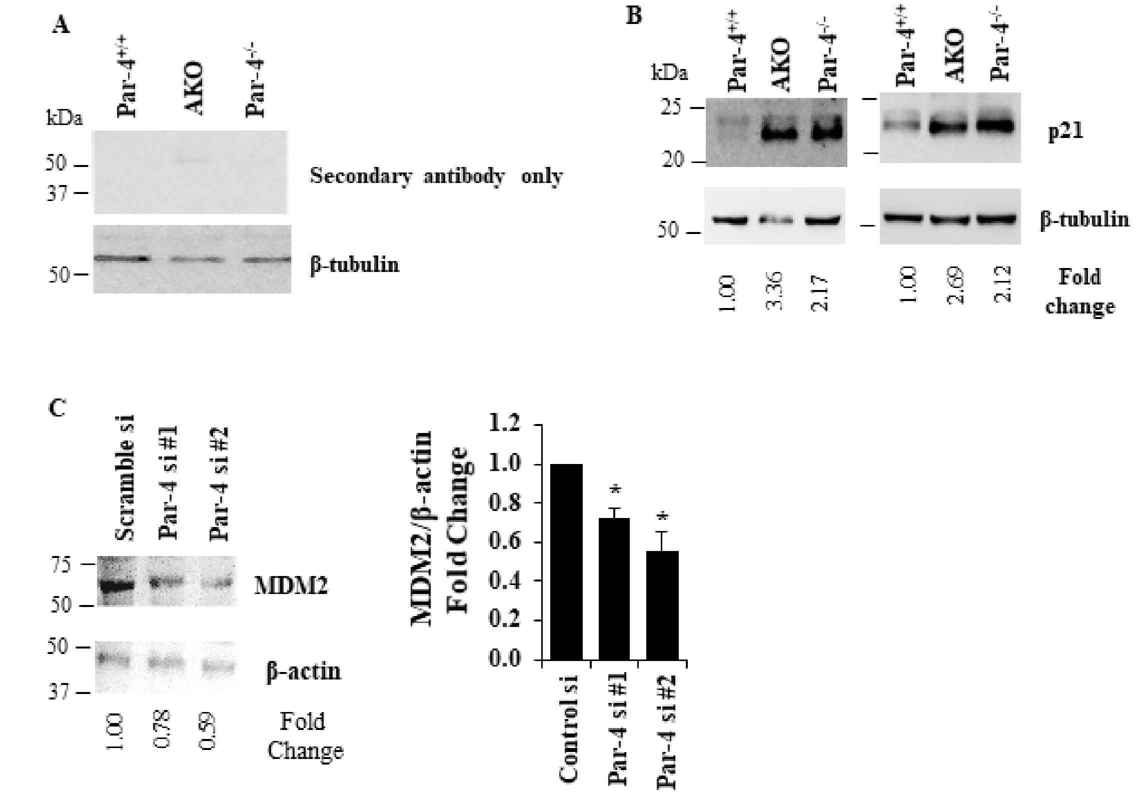


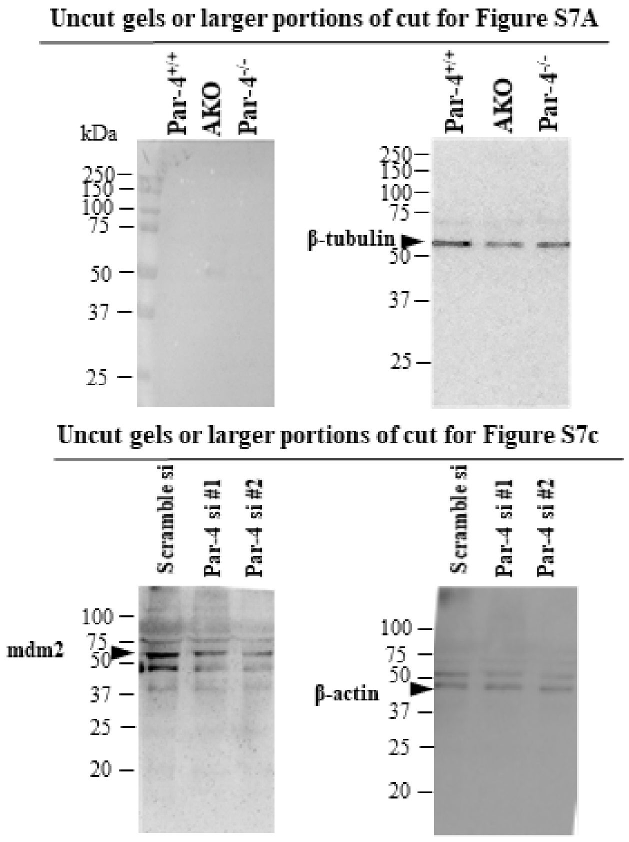


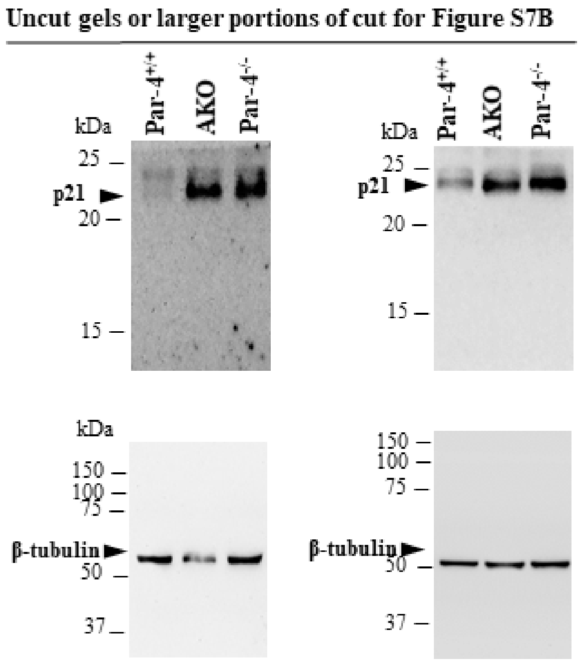


Supplementary Figure S7

**Figure S7. Par-4 regulates p53. (A) p53 mouse antibody 1C12 is specific for p53.** Whole tissue extracts from visceral adipose tissue from AKO, Par-4$^{-/-}$ and Par-4$^{+/+}$ mice were subjected to SDS-PAGE, transferred to PVDF membranes, and the blots were incubated solely with goat anti-mouse secondary antibody to validate specificity of the p53 primary antibody used in Fig 7B. As control, β-tubulin was used to demonstrate the presence of protein on the gel. **(B) p21 is upregulated in the adipose tissue of AKO and Par-4$^{-/-}$ mice**. Visceral adipose tissues from AKO, Par-4$^{-/-}$ and Par-4$^{+/+}$ mice were subjected to western blot analysis for p21 and β-tubulin. p21 levels were normalized to β-tubulin and fold change is shown in 3-month-old **(left panel)** and 6-week-old **(right panel)** mice**. (C) Mdm2 is downregulated in MEFs.** WT MEFs were transfected with either control siRNA or two different Par-4 siRNAs (#1 and #2 from Dharmacon). Whole-cell extracts from transfected cells were collected after 24h of transfection and subjected to western blot analysis for Mdm2 using β-actin as a loading control. **(C)** Mean ± SEM, *adjusted $P < 0.05$ by the Student's *t*-test with Bonferroni method.

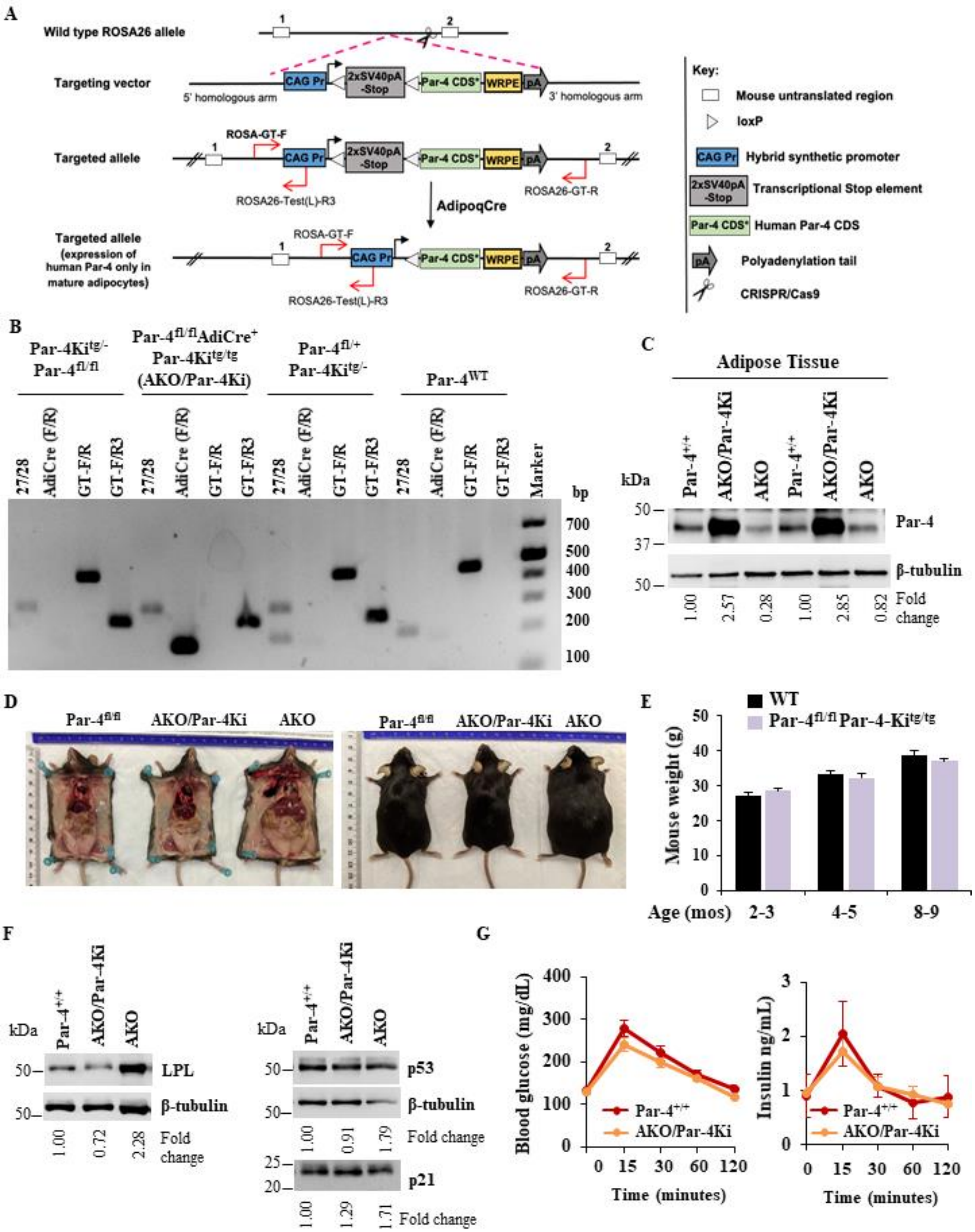


Supplementary Figure S8 (continues on the next page)

**Uncut gels or larger portions of cut gels for Supplementary Figure S8C**

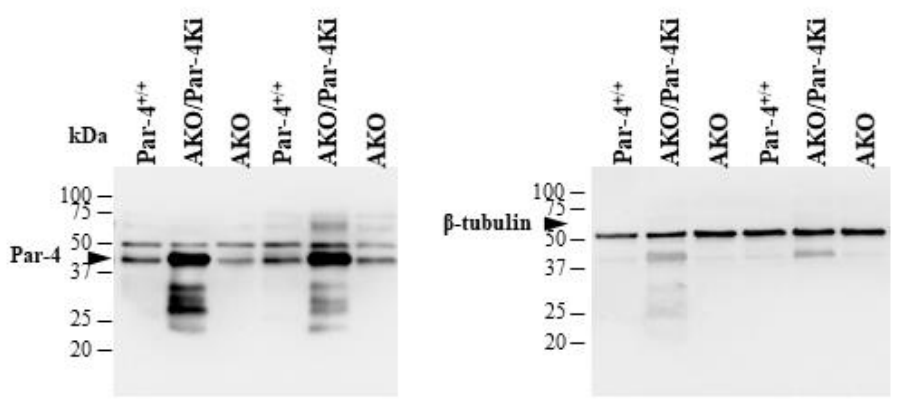


**Uncut gels or larger portions of cut gels for Supplementary Figure S8F**

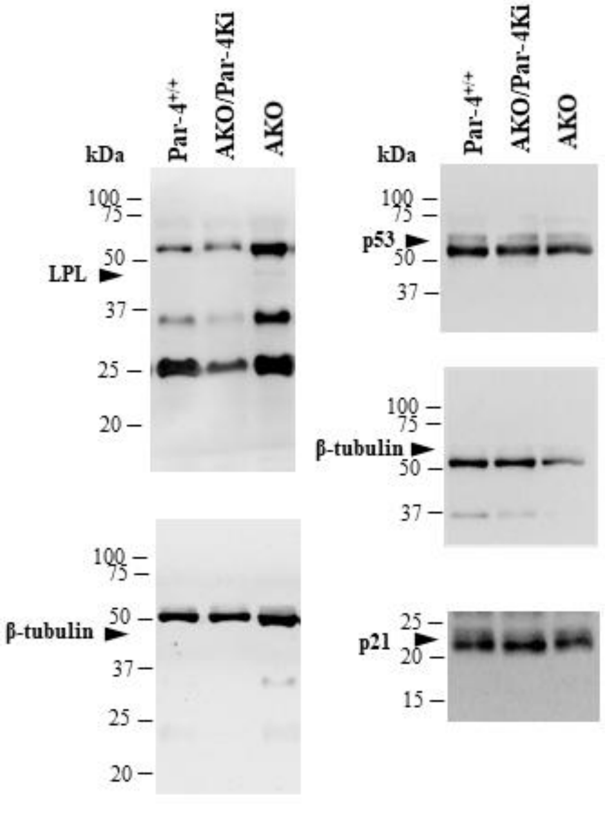


**Supplementary Figure S8 (Continued)**

**Supplementary Figure S8 (Related to Figure 8). Genotype and phenotype of AKO/Par-4Ki mice. (A) Strategy for generation of AKO/Par-4Ki mice.** A targeting vector was inserted into the ROSA26 allele using CRISPR/Cas9. The targeting vector consisted of a CAG hybrid promoter (a CMV enhancer fused with the chicken beta-actin promoter), a loxed SV40 stop sequence, the human Par-4 CDS, the WPRE (Woodchuck Posttranscriptional Regulatory Element) and a polyadenylation tail. Presence of the transgene (tg) was confirmed using the primer pairs: ROSA-GT-F/ROSA-Test(L)-R3. Primer pair

ROSA-GT-F/ROSA-GT-R identified the WT sequence. When knock-in transgenic mice (Par-4Ki$^{tg/tg}$) are crossed with Adipoq mice (AKO in our system), the Stop sequence is removed, and the expression of human Par-4 is driven in mature adipocytes. **(B) Genotyping of conditional AKO/ Par-4Ki mice.** DNA from tail clips of Par-4$^{fl/fl}$ Par-4Ki$^{tg/-}$AdiCre$^{-}$, Par-4$^{fl/fl}$ Par-4Ki$^{tg/tg}$ AdiCre$^{+}$ (AKO/Par-4Ki), Par-4$^{fl/+}$ Par-4Ki$^{tg/-}$AdiCre$^{-}$ and Par-4$^{WT}$ mice were subjected to PCR using the indicated optimized primers for the knock-in, the Par-4 transgenes, and the recommended primers for Adiponectin-Cre according to Jackson Laboratory. The resultant DNA fragments were electrophoresed on agarose gels and stained with ethidium bromide. As expected, primer set 27/28 detected a 326 bp fragment in Par-4$^{fl/fl}$ mice, 213 bp fragment in Par-4$^{+/+}$ (wild type) mice; the Adipoq-Cre forward (F) and reverse (R) primer set detected a 190 bp fragment in Adipoq-Cre mice. Primer GT-F/R3 identified a single fragment for the transgene at 278bp; primer pair GT-F/R detected a 469bp fragment for the wild-type allele. **(C) Par-4 expression in AKO/Par-4Ki mice.** Protein extracts from visceral fat of Par-4$^{+/+}$, AKO/Par-4Ki and AKO mice were subjected to western blot analysis for Par-4 and β-tubulin. Par-4 levels were normalized to β-tubulin protein levels. As expected, total Par-4 protein levels are increased in AKO/Par-4Ki adipose tissue compared to Par-4$^{+/+}$ control and AKO mice. **(D) Fat accumulation in AKO/Par-4Ki mice.** Overall body size and fat accumulation are similar in control and AKO/Par-4Ki mice. AKO mice are heavier and show increased fat accumulation when compared to either control or AKO/Par-4Ki mice (photo depicts 7–11-month-old mice) **(E) Weight progression of control mice**. Weight gain of age-matched WT (n=30, 9, 6), Par-4$^{fl/fl}$ Par-4-Ki$^{tg/tg}$ (n=12, 8, 7) control mice is similar across 9 months of follow up. **(F) Reversal of increased expression of p53, p21 and LPL levels upon re-expression of Par-4.** Expression of p53, p21, and LPL in the adipose tissue of Par-4$^{+/+}$, AKO/Par-4Ki and AKO mice was examined by western blot analysis. **(G) Glucose levels and insulin levels in AKO/Par-4Ki and Par-4$^{+/+}$ mice.** Glucose was orally administered at the indicated time points to 10-month-old Par-4$^{+/+}$ (n=9) and AKO/Par-4Ki (n=8) female mice maintained on standard chow. Plasma levels of glucose were determined. Plasma levels of insulin at different time points were determined in 10-month-old Par-4$^{+/+}$ (n=3) and AKO/Par-4Ki (n=4) female mice after oral glucose administration. **(E, G)** Mean ± SEM, adjusted $P > 0.05$ by the Student's *t*-test with Bonferroni method.

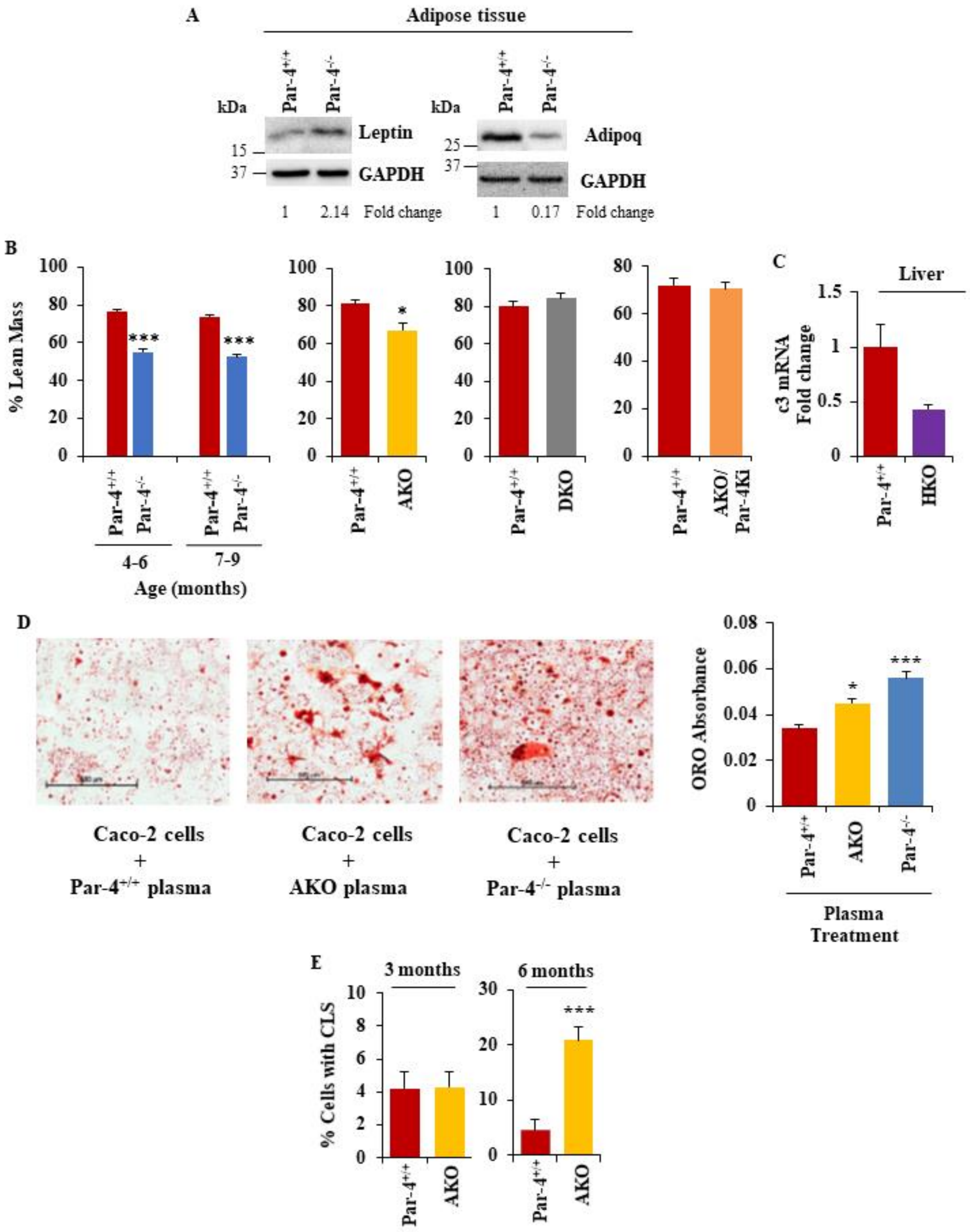


Supplementary Figure S9 (continues on the next page)

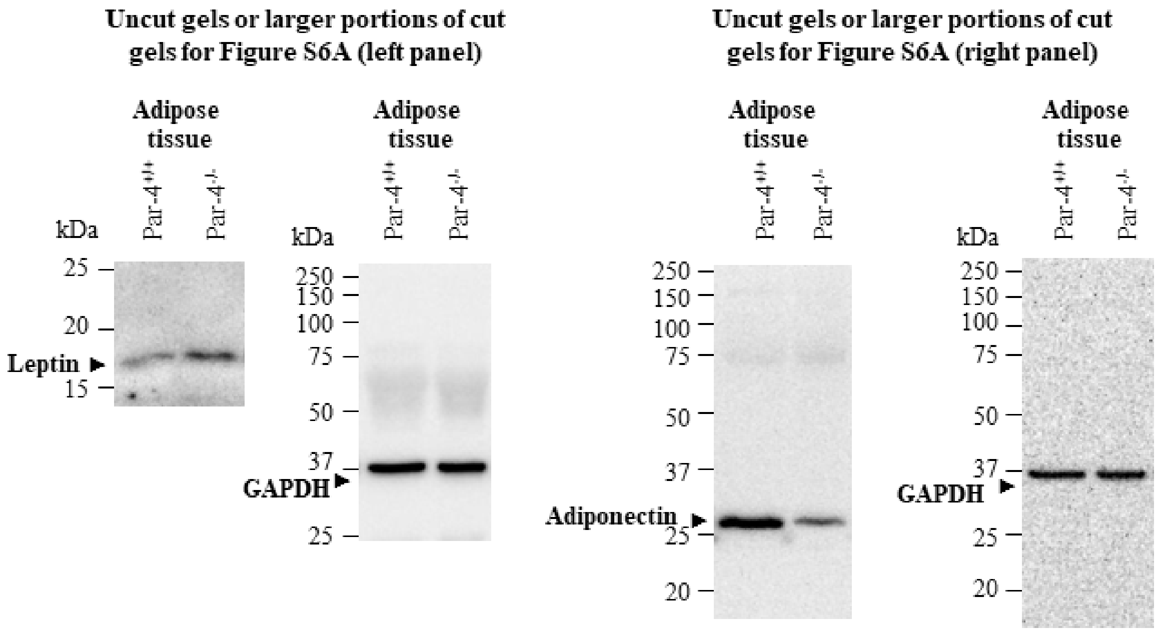


Supplementary Figure S9 (Continued)

**Supplementary Figure S9. Leptin, adiponectin, and lean mass levels in Par-4 knockout mice, C3 levels in HKO mice, and crown-like structures in AKO mice. (A) Leptin and adiponectin levels in Par-4$^{-/-}$ mice.** Lysates from visceral adipose tissues of Par-4$^{+/+}$ and Par-4$^{-/-}$ mice were prepared and subjected to western blot analysis for leptin and adiponectin. Note leptin was increased in the fat of Par-4$^{-/-}$ mice **(left panel)** and adiponectin was decreased in the fat of Par-4$^{-/-}$ mice **(right panel). (B) Lean mass in Par-4$^{+/+}$, Par-4$^{-/-}$, AKO, DKO, and AKO/Par-4Ki mice.** Lean mass percentages are shown for the same mice for which percent fat mass is shown in Figures 1B, 3B, 6B and 8B. **(C) C3 levels are not upregulated in the liver of HKO mice.** RNA samples from Par-4$^{+/+}$ and HKO livers (n=3) were subjected to qPCR analysis for C3 and 18S rRNA. C3 levels were normalized to 18S rRNA and fold change was calculated. **(D) AKO and Par-4$^{-/-}$ plasma promote triglyceride absorption.** Caco2 cells were treated with olive oil in the presence of 10% plasma from either Par-4$^{+/+}$, AKO or Par-4$^{-/-}$ mice for 24 h. After treatment, cells were fixed and stained with ORO. Slides was analyzed under the microscope. Scale bar = 500 μm. **(E) Quantification of macrophage infiltration in visceral fat of 6-month-old AKO mice.** Visceral fat from Par-4$^{+/+}$ and AKO mice that was subjected to IHC for F4/80 macrophage marker (Figure 3C), was analyzed under the microscope and crown-like structures (Figure 3C, arrows) were quantified and expressed as a percentage of the total number of adipocytes. **(B, C, D, E)** Mean ± SEM, **P* < 0.05, ****P* < 0.001 by the Student’s *t*-test or with Bonferroni correction.

Uncut gels or larger portions of cut gels for Figure 2A

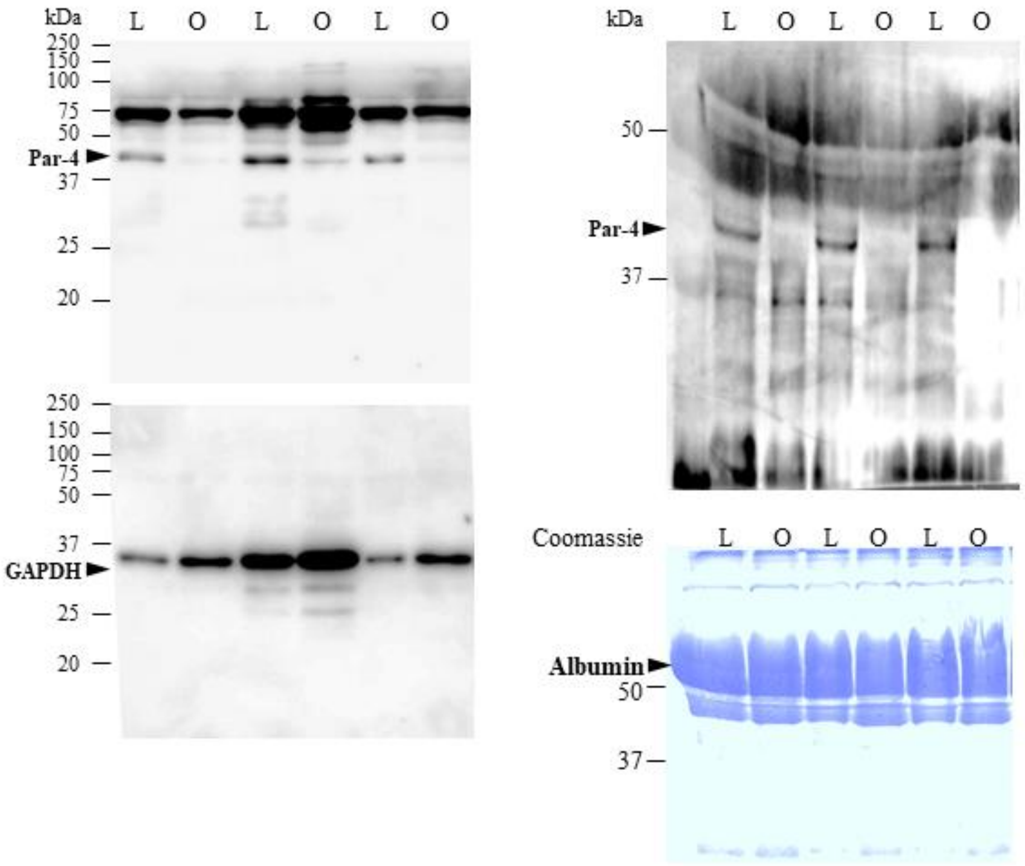


Uncut gels or larger portions of cut gels for Figure 2C

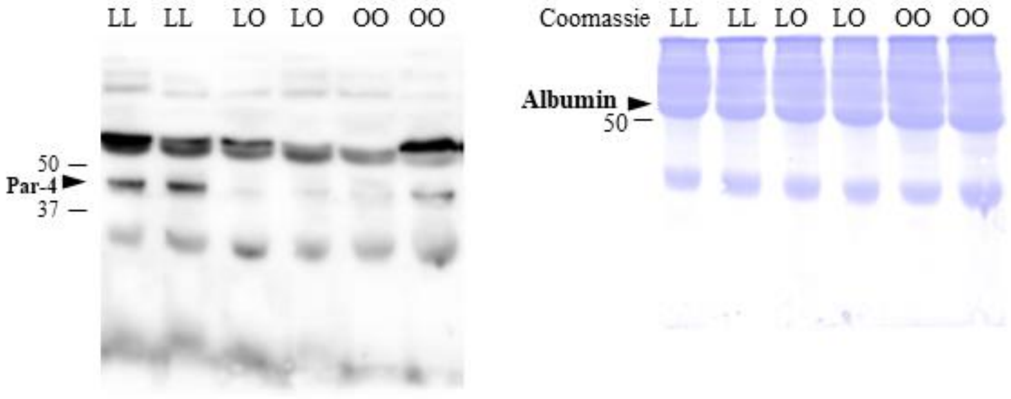


**Supplementary Figure S10. Uncut gels or larger portions of cut gels related to Figure 2A and 2C.**

**Uncut gels or larger portions of cut gels – Related to Figure 4C**

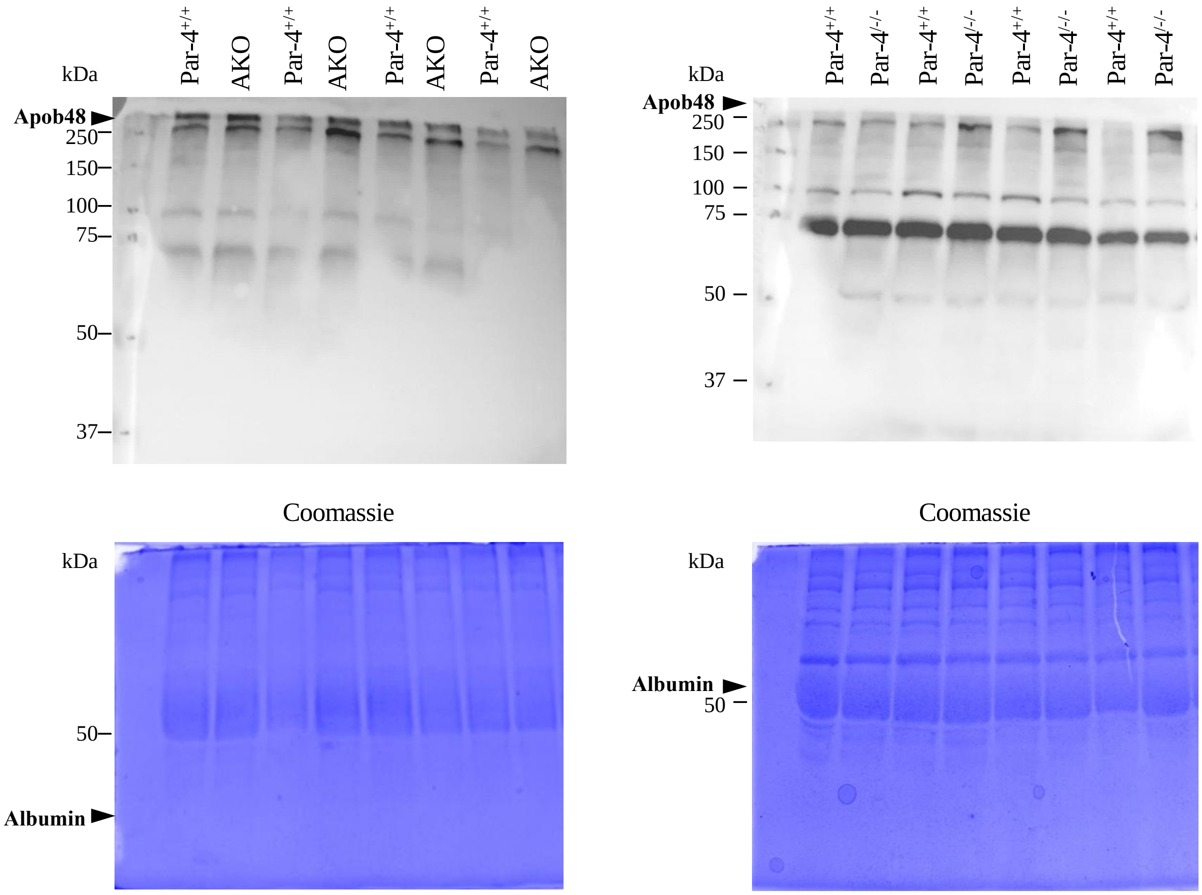


**Supplementary Figure S11. Uncut gels or larger portions of cut gels related to Figure 4C.**

**Uncut gels or larger portions of cut gels for Figure 5D**

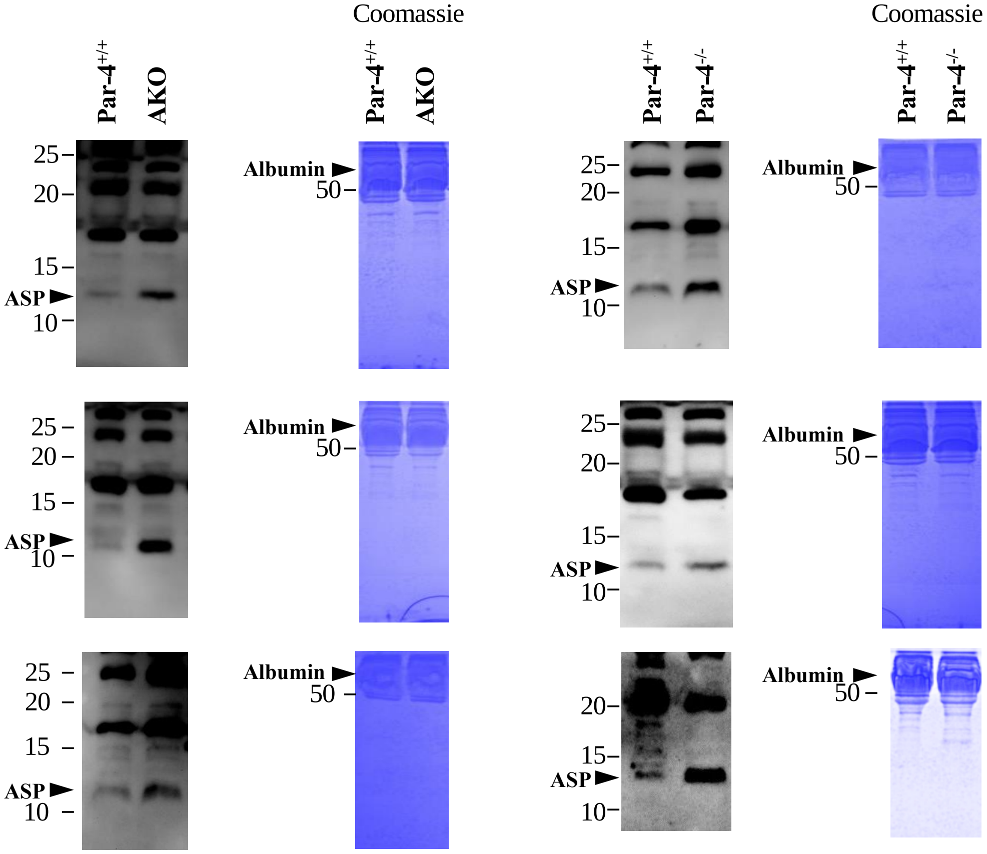


**Figure S12. Uncut gels or larger portions of cut gels related to Figure 5D.**

**Uncut gels or larger portions of cut gels for Figure 6C**

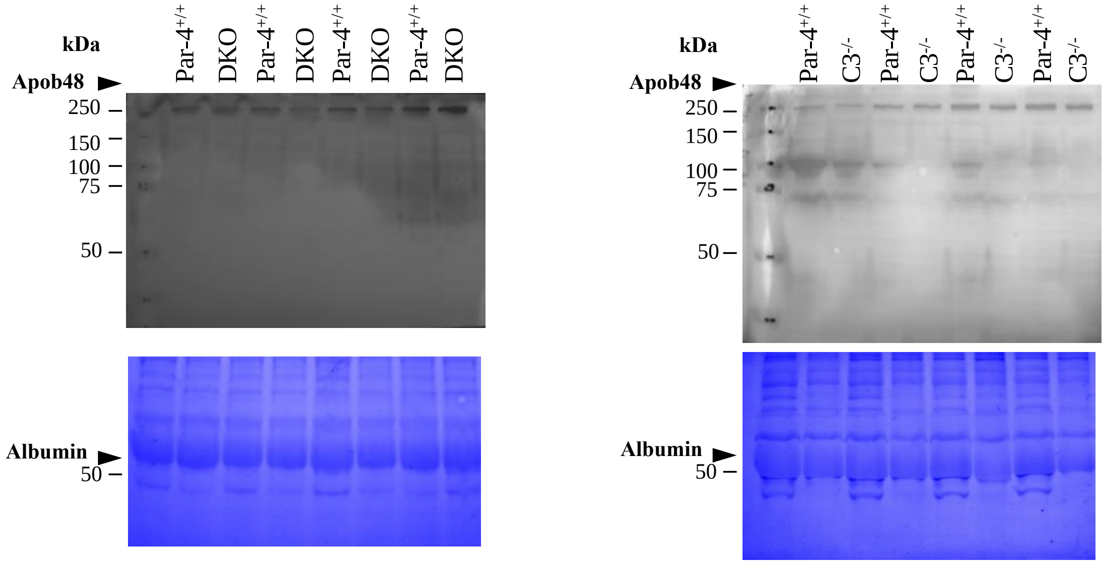


**Uncut gels or larger portions of cut for Figure 6D**

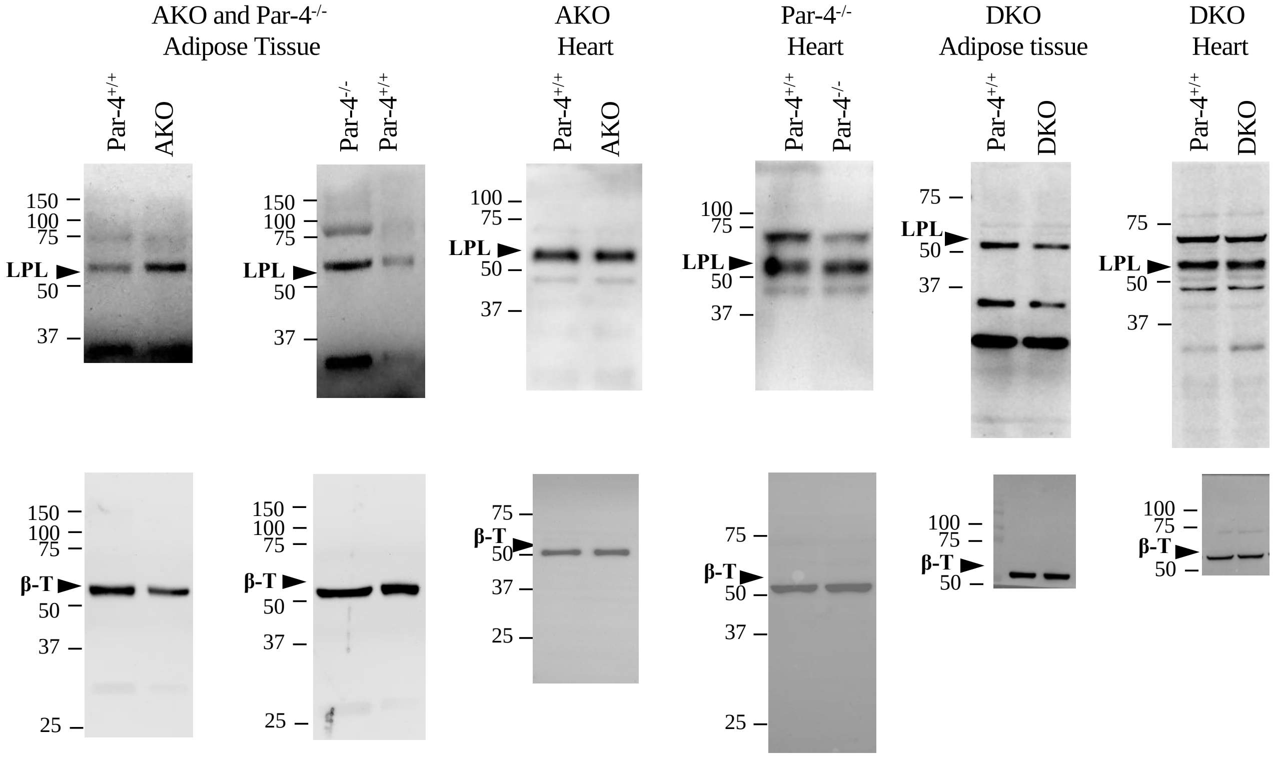


**Figure S13. Uncut gels or larger portions of cut gels related to Figure 6C and 6D.**
**Note: β-T = β-Tubulin**

**Uncut gels or larger portions of cut gels for Figure 7A**

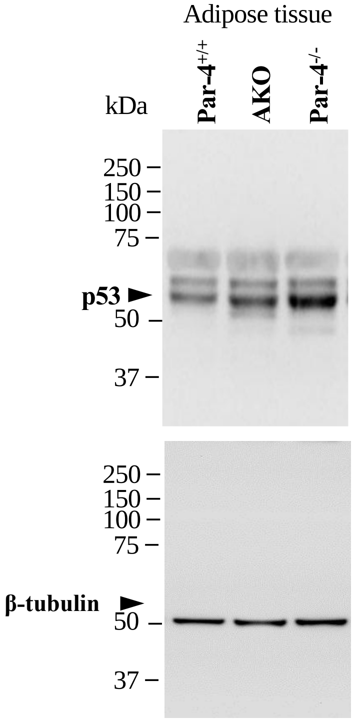


**Uncut gels or larger portions of cut gels for Figure 7C**

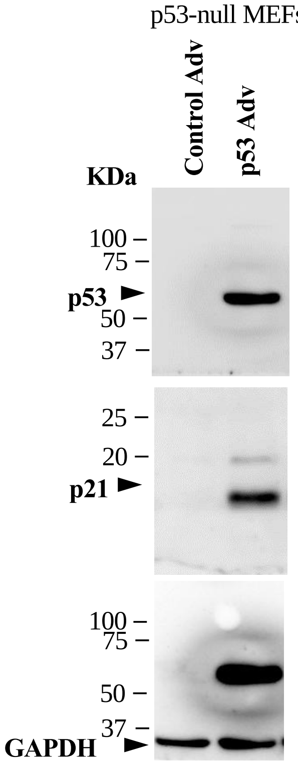


**Uncut gels or larger portions of cut gels for Figure 7E**

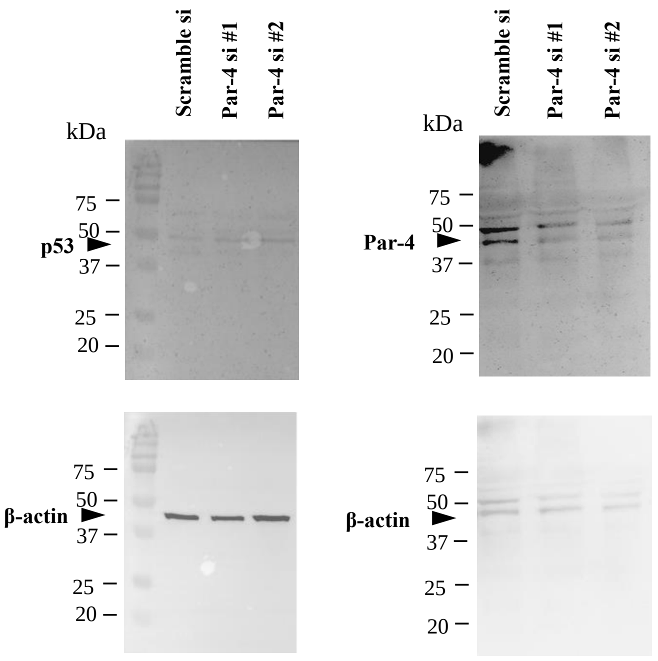


**Figure S14. Uncut gels or larger portions of cut gels related to Figure 7A, 7C and 7E.**

**Uncut gels or larger portions of cut for Figure 8C**

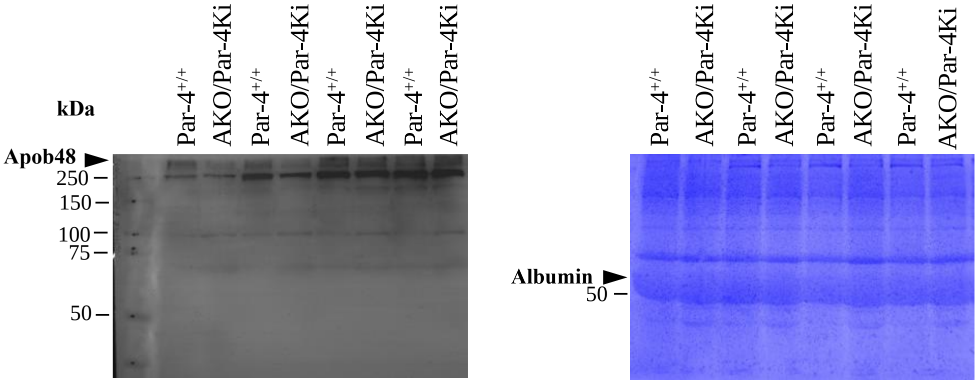


**Uncut gels or larger portions of cut for Figure 8D**

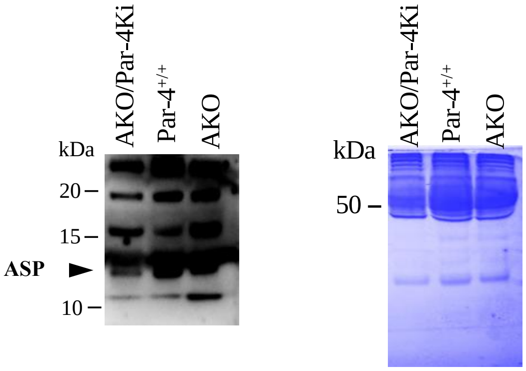


**Figure S15. Uncut gels or larger portions of cut gels related to Figure 8C and 8D.**

**Table S1. Additional information on study participants.**

| Characteristics | No. of patients (%) |
|---|---|
| **Age** | |
| **Median (SD; range)** | 53.5 (4; 46.1-66.9) |
| **Systolic Blood Pressure** | |
| **Median (SD; range)** | 138 (19.6; 98-192) |
| **Caloric Intake** | |
| **Median (SD; range)** | 2142.3 (633.8; 1147.11-3812.98) |
| **Missing** | 4(3.9) |
| **Glucose** | |
| **Median (SD; range)** | 4.9 (1.3; 3.9-12.9) |
| **Cholesterol** | |
| **Median (SD; range)** | 6.1 (1; 4.04-8.79) |
| **Tryglicerides** | |
| **Median (SD; range)** | 1.1 (0.7; 0.52-3.6) |
| **HDL** | |
| **Median (SD; range)** | 1.4 (0.4; 0.66-2.91) |
| **LDL** | |
| **Median (SD; range)** | 4.1 (0.9; 2.6-6.6) |
| **Insulin** | |
| **Median (SD; range)** | 6 (9; 2.9-66) |
| **Missing data** | 1(1) |
| **CRP** | |
| **Median (SD; range)** | 0.2 (0.3; 0.01-1.22) |
| **Missing data** | 2(2) |
| **BMI** | |
| **Median (SD; range)** | 23.7 (6.4; 18.25-41.71) |
| **Par-4 index** | |
| **Median (SD; range)** | 0.5 (0.4; 0-1.58) |
| **Sex** | |
| **f** | 63 (61.8) |
| **m** | 39 (38.2) |
| **Hypertension** | |
| **No** | 42 (41.2) |
| **Yes** | 60 (58.8) |
| **Diabetes** | |
| **No** | 89 (87.3) |
| **Yes** | 13 (12.7) |

| | |
|---|---|
| **Quartiles of Physical Activity from Entire Background Population** | |
| **1** | 20 (19.6) |
| **2** | 16 (15.7) |
| **3** | 24 (23.5) |
| **4** | 26 (25.5) |
| **Missing data** | 16 (15.7) |
| **Smoking** | |
| **No** | 72 (70.6) |
| **Yes** | 30 (29.4) |
| **Stroke** | |
| **No** | 102 (100) |
| **Yes** | 0 |
| **Coronary Event** | |
| **No** | 100 (98) |
| **Yes** | 2 (2) |
| **Hypertension Medication** | |
| **No** | 83 (81.4) |
| **Yes** | 19(18.6) |
| **Lipid Lowering Medication** | |
| **No** | 101 (99) |
| **Yes** | 1 (1) |
| **Diabetes Medication** | |
| **No** | 98(96.1) |
| **Missing data** | 4(3.9) |

Yes = Present and No = Absent for the following: Hypertension, Diabetes, Smoking, Coronary Event, Hypertension Medication, Lipid Lowering Medication, and Diabetes Medication.

Age is shown in years; Systolic Blood Pressure in mmHg; Caloric Intake in kcal/day; Glucose, Cholesterol, Triglycerides, HDL and LDL in mmol/L.

**Table S2. Genes identified by ChIP-Seq in 3T3-L1 cells after ChIP with Par-4 antibody.**

| Gene Name | Nearest Ensembl | Annotation | Distance to TSS |
|---|---|---|---|
| Nup107 | ENSMUSG00000052798 | intron (NM_134010, intron 1 of 27) | 2023 |
| Nup107 | ENSMUSG00000052798 | intron (NM_134010, intron 5 of 27) | 9779 |
| Nup107 | ENSMUSG00000052798 | intron (NM_134010, intron 1 of 27) | 1420 |
| Nup107 | ENSMUSG00000052798 | intron (NM_134010, intron 5 of 27) | 8390 |
| Nup107 | ENSMUSG00000052798 | intron (NM_134010, intron 12 of 27) | 18349 |
| Nup107 | ENSMUSG00000052798 | intron (NM_134010, intron 1 of 27) | 2107 |
| Mdm2 | ENSMUSG00000020184 | intron (NM_010786, intron 1 of 11) | 315 |
| Mdm2 | ENSMUSG00000020184 | intron (NM_027468, intron 7 of 8) | 33832 |
| Mdm2 | ENSMUSG00000020184 | exon (NM_001288586, exon 8 of 11) | 15792 |
| Mdm2 | ENSMUSG00000020184 | exon (NM_001288586, exon 9 of 11) | 18025 |
| Mdm2 | ENSMUSG00000020184 | intron (NM_027468, intron 7 of 8) | 32991 |
| Mdm2 | ENSMUSG00000020184 | Intergenic | -6024 |
| Mdm2 | ENSMUSG00000020184 | promoter-TSS (NM_001288586) | 56 |
| Ifngas1 | ENSMUSG00000112230 | Intergenic | -54479 |
| Ifngas1 | ENSMUSG00000112230 | Intergenic | -54545 |
| Ifngas1 | ENSMUSG00000112230 | Intergenic | -65684 |
| Ifngas1 | ENSMUSG00000112230 | Intergenic | -66608 |
| Ifngas1 | ENSMUSG00000112230 | Intergenic | -55478 |
| Lin7a | ENSMUSG00000019906 | Intergenic | -58610 |
| Lin7a | ENSMUSG00000019906 | Intergenic | -39562 |
| Lin7a | ENSMUSG00000019906 | Intergenic | -58142 |
| Lin7a | ENSMUSG00000019906 | Intergenic | -47052 |
| Lin7a | ENSMUSG00000019906 | Intergenic | -16022 |
| Lin7a | ENSMUSG00000019906 | Intergenic | -23265 |
| Lin7a | ENSMUSG00000019906 | Intergenic | -23009 |
| Lin7a | ENSMUSG00000019906 | Intergenic | -32214 |
| Lin7a | ENSMUSG00000019906 | Intergenic | -27612 |
| Lin7a | ENSMUSG00000019906 | Intergenic | -57111 |
| Lin7a | ENSMUSG00000019906 | Intergenic | -52589 |
| Lin7a | ENSMUSG00000019906 | Intergenic | -58625 |
| Il1rapl1 | ENSMUSG00000052372 | intron (NM_001160403, intron 5 of 10) | 877274 |
| Il1rapl1 | ENSMUSG00000052372 | intron (NM_001160403, intron 5 of 10) | 877273 |
| Myf5 | ENSMUSG00000000435 | Intergenic | 36245 |
| Myf5 | ENSMUSG00000000435 | Intergenic | 36147 |
